# Behavior change interventions to promote adoption of e-bike shared mobility in a rural area: evidence from a mixed-method field trial

**DOI:** 10.3389/fpsyg.2025.1569176

**Published:** 2025-05-20

**Authors:** Mark Wilson, Lorraine Whitmarsh

**Affiliations:** ^1^Department of Psychology, University of Bath, Bath, United Kingdom; ^2^Centre for Climate Change and Social Transformations (CAST), University of Bath, Bath, United Kingdom

**Keywords:** behavior change intervention, mode shift, e-bike shared mobility, low-carbon travel, climate mitigation, local authority partnership

## Abstract

Encouraging a shift to sustainable travel modes is essential for achieving net zero goals. This mixed-method study investigates the adoption of e-bike shared mobility in a rural context. Partnering with Cornwall Council and the shared e-bike provider Beryl, the study trialed two behavior change interventions to encourage people to use active modes; 151 residents and 14 Council staff took part. The two interventions were: (1) free Beryl bike credits, so people gain experience of using the e-bike share scheme on a trial basis, and (2) the ‘Pen portraits’ visioning tool, which uses evidence-based narratives to motivate people to consider how they could reduce car use in their daily lives. The effectiveness of the interventions was assessed over a four-week period through comparison to a control condition. During the study, uptake of e-bike share increased from 7 to 31% for residents, and from 29 to 71% for Council staff. Commuting and leisure or exercise were the most common journey purposes, although the bikes were also used as a component of multimodal travel. Beryl bikes encouraged mode shift for short journeys (1–2 miles), with 28% of e-bike share journeys substituting private car use, resulting in estimated carbon emission savings of 96–626 g CO_2_ per journey. Relative to the control group, more people in each of the three intervention groups used a Beryl bike (Control group = 21% of residents used Beryl, compared to: the visioning tool = 31%; Beryl bike credits = 37%; and Beryl bike credits plus the visioning tool = 36%). However, these differences are not statistically significant. Participants reported strong agreement that e-bike share provides a range of practical benefits such as reduced concern about bike maintenance and theft, as well as co-benefits such as providing exercise and improving mental health. The article concludes with a discussion of the practical and analytical challenges for conducting behavior change interventions in real-world settings. These findings are relevant for local authorities who are interested in tools and behavioral approaches for engaging with the public on low-carbon travel.

## 1 Introduction

Swift decarbonization is not only a technical challenge, but also a societal challenge that requires a fundamental shift away from high-carbon ways of living, and this positions people at the center of climate action (Moore et al., 2021; Verfuerth et al., 2024). In terms of engaging people to adopt low-carbon behaviors, local authorities have a key role to play in delivering contextually relevant infrastructural and regulatory interventions (Bulkeley et al., 2011). Local authorities also have an organizational role, with the capacity to introduce pro-environmental targets and working practices among their workforce, and so leading by example for other large employers. Local authorities are therefore increasingly interested in tools and behavioral approaches for mainstreaming climate action, engaging with the public, and maximizing impact with limited resources (Mees et al., 2019; Braunholtz-Speight et al., 2023).

Encouraging people to use sustainable modes of travel is one focus area for local authorities, not only because of the high emission intensity of private car use, but also the negative health impacts associated with air pollution (Allen et al., 2023; UK Health Security Agency, 2023). Municipal strategies include ‘push’ measures which discourage car use (e.g., traffic congestion charges), as well as ‘pull’ measures to encourage greater use of low-carbon modes (e.g., subsidized bus tickets) (Whitmarsh et al., 2021; Powell and James, 2023). One of these ‘pull’ strategies is providing shared mobility options, such as car clubs and public bike share schemes, which reduce the need for private vehicle use and ownership. The use of bike share, and e-bike share in particular, has seen a rapid increase in many countries in recent years. For example, the number of bike/e-bike share users in the UK has more than doubled since 2021; there are now over 2 million active users and e-bike share comprises 59% of all bike share journeys (CoMoUK, 2024). Considering this growing uptake, it is important for local authorities to understand how and why e-bike share is used in different contexts, and how effective it is in reducing carbon emissions.

The literature on bike/e-bike share focuses on users’ journey purposes and identifying mode shift opportunities. One study finds a large proportion of dockless e-bike trips are for commuting and that the availability of the e-bikes and public transport services are key determinants of demand. The distances traveled by e-bike are comparable with the distances for public transport and taxi journeys (Guidon et al., 2019). Another study found awareness of e-bike share does not necessarily influence commuting behavior (Handy and Fitch, 2022). In terms of mode shift, one study finds e-bike share primarily substitutes public transport rather than car, and e-bikes are often used for the first or last mile of journeys (Bieliński et al., 2021). In contrast, two studies identify high car substitution rates of 37 and 28% (CoMoUK, 2022; Fukushige et al., 2023). Some authors find mode shift varies depending on the journey distance or purpose; for trips of less than one mile, shared e-bike is more likely to substitute walking, whereas for longer journeys or non-commute trips, shared e-bike is more likely to substitute car use (Fukushige et al., 2021). Multiple studies find significant carbon emission savings from using private e-bikes, relative to other modes (Winslott Hiselius and Svensson, 2017; Bucher et al., 2019; McQueen et al., 2020; Philips et al., 2022; Brand et al., 2022). Other authors focus specifically on e-bike share schemes and find emission reduction of up to 75% (Zhou et al., 2023), or 108–120 g CO_2_ km (Li et al., 2023).

Several studies identify the predictors of bike/e-bike share adoption. Some authors highlight cost savings and convenience relative to using a car, a desire for physical exercise, and reduced concern around bike theft as key motivations (CoMoUK, 2022; Teixeira et al., 2023; Bartling, 2023). Perceived ease of use, perceived usefulness, and the positive opinions of others also encourage uptake (Li et al., 2022), in line with technology adoption theory (Straub, 2009). Other key factors include a high population density and the proximity of parking bays to public transport hubs, sports centers, and bike trails (He et al., 2019). The assisted power to cycle up steep hills, travel longer distances, and overcome health difficulties or low fitness levels is important for some users, which suggests e-bike share may play a role in making active travel more inclusive (Bieliński et al., 2021; CoMoUK, 2022). Investigating user profiles, students are typical early adopters but e-bike share is increasingly used by educated middle-aged workers (Julio and Monzon, 2022). E-bike share schemes encourage active travel among people aged 55 years or older (Fukushige et al., 2021). Men are more likely to use bike share than women (Barbour et al., 2019; CoMoUK, 2022), although e-bike sharing motivates women to make trips who would otherwise use a car (Fukushige et al., 2021). Some authors find road safety concerns and inconvenience are important barriers to adoption (Fishman et al., 2014).

A limited number of studies trialed interventions to encourage mode shift and their findings are promising. The loan of an e-bike for 2 weeks resulted in participants’ habitual association with car use weakening significantly, both for participants who bought an e-bike after the trial and those who did not (Moser et al., 2018). A similar study found car use for commuting decreased from 88% before an eight-week e-bike loan to 63% 3 months later. E-bike use increased from 2 to 18% in the same period (Ton and Duives, 2021). A third study found the loan of an e-bike for 2–4 weeks did not influence those who regularly use a conventional bike, but it was effective at reducing car use amongst drivers (Fyhri et al., 2017). A more recent study, conducted in Cornwall, found 89% of participants reduced their car travel and 29% subsequently purchased an e-bike, following a 3-month loan of an e-bike (Shergold et al., 2023).

The broader behavior change evidence base highlights several important considerations for intervention design. Combining interventions (e.g., information and incentives) tends to be more effective than component interventions (Poortinga and Whitaker, 2018) because of the multiple drivers of behavior (including capability, opportunity and motivational factors detailed in the COM-B model; Michie et al., 2011). Importantly, information alone tends to be relatively ineffective for motivating pro-environmental behaviors (Nisa et al., 2019). Interventions which apply a co-benefits framing, for instance emphasizing potential health and environmental benefits, can support behavior change (Wolstenholme et al., 2020). Interventions to encourage car-reduction are most effective when they remove barriers to using alternative travel modes (e.g., cost, inconvenience) and are co-produced with communities to ensure a tailored approach that addresses local needs and increases perceived procedural fairness (Whitmarsh and Frost, 2024). However, the evidence base on car-reduction interventions remains methodologically weak, with few robust experimental field trials (Graham-Rowe et al., 2011; Okraszewska et al., 2024). Moreover, few behavior change studies quantify the environmental impact of the intervention. Finally, theoretical literature on technology adoption explores the interplay of individual, technological, and societal factors that dictate how rapidly a technology will spread throughout society. The decision to adopt a new technology is rarely based solely on economic or pragmatic considerations, but encompasses social norms, values, and personal factors (Straub, 2009; Whittle and Whitmarsh, 2022).

In summary, there is extensive literature on the benefits and predictors of bike share adoption and e-bike ownership (Jones et al., 2016; de Haas et al., 2022; Winters et al., 2019; Biehl et al., 2019). The literature on e-bike share is emerging; when this study was conducted, the only previous studies in the UK context were a case study by Devon County Council (Thomas and Devon County Council, 2019) and an annual survey conducted by the charity Collaborative Mobility UK (CoMoUK, 2022). Reflecting the roll out of e-bike share schemes primarily in densely populated areas, previous studies have explored e-bike share adoption in large cities such as Shanghai, Sacramento, and Gdańsk (Zhou et al., 2023; Fukushige et al., 2023; Bieliński et al., 2021). The contribution of this study is twofold: (1) to investigate the uptake of e-bike shared mobility in rural settings, where travel behaviors and the context of adoption differ from cities, and (2) to test behavioral science approaches which local authorities can use to motivate modal shift.

This article reports the findings of an intervention study to encourage adoption of active modes of travel (i.e., walking, wheeling, or cycling). The study was conducted in Cornwall, a predominantly rural county in the southwest of the UK, and was co-designed with Cornwall Council, a unitary local authority. In 2022/23, Cornwall became one of the first rural counties in the UK to introduce an e-bike share scheme, through a partnership between the Council and the shared e-bike provider, Beryl. The Council were interested in ways to motivate uptake of Beryl bikes and two interventions were trialed with Cornwall residents and Council staff, measuring behavior change across a four-week period. The effectiveness of the interventions was assessed through comparison to a control condition. There were four aims of this study:

1. To measure participants’ perceptions of e-bike share.
2. To understand how e-bike share is used in rural settings by characterizing users and investigating journey purpose, frequency, distance, and multimodal travel.
3. To quantify the potential mode shift emission reduction, from private car to e-bike share.
4. To determine whether the two behavior change interventions were effective in encouraging uptake of e-bike share and other sustainable modes.

## 2 Methodology

This is a mixed-method article; qualitative findings from an initial scoping study were used to inform the objectives and design of the behavior change intervention study.

### 2.1 Study 1—Focus groups

Online focus groups were conducted between December 2022 and January 2023 to explore Cornwall residents’ (*n* = 26) views on the feasibility of five low-carbon travel modes (public transport, active travel, car-sharing, electric vehicles, multi-modal travel) in the context of four frequent journeys (commuting to their place of work or study, shopping or accessing local services, leisure or visiting family and friends, and the school run, if applicable). Perceptions of the relative advantages and disadvantages of these travel modes were explored, along with the potential barriers and enablers of using each mode. The role of habits, key life events, social norms, and the expectations of household members on participants’ travel behaviors were also investigated. Finally, participants ranked five ‘push’ measures to reduce travel-related carbon emissions (e.g., increased car parking charges in town centers), as well as five ‘pull’ measures (e.g., cheaper public transport). The anticipated effects of these measures on their travel behaviors were discussed. The focus group protocol can be found in Supplementary material.

The study was promoted via Council communication channels such as their website and resident newsletters. Participants were recruited from five demographics to represent varying travel experiences and needs in Cornwall: urban residents; rural residents; young adults aged 16–22; people from low-income households; and people with a long-term health condition or disability. Table 1 shows those with a disability were underrepresented in the final sample, but the sampling criteria were fulfilled for the remaining groups (i.e., a minimum of five participants per group). Participation was incentivized through a £20 gift voucher for each respondent. Council staff were not recruited for Study 1, as their views on sustainable travel had been collected in a previous study (see: Player et al., 2022; Toy et al., 2023).

**Table 1:** Sociodemographic characteristics of the focus group participants (residents, *n* = 26).

Sociodemographic characteristic	Frequency	%
Gender		
Female	15	57.7
Male	11	42.3
Have a longstanding health condition or disability	1	3.8
Are a young adult aged 16–22	5	19.2
Live in an urban area (i.e., suburbs or center of a large town or city)^a^	10	38.5
Live in a rural area (i.e., rural town or village)	16	61.5
Live in a low-income household^b^	5	19.2
Own or have regular access to a car	22	84.6
Have an undergraduate or postgraduate degree	16	61.5
Employment status		
Employed (full- or part-time) or self-employed	14	53.8
Retired	6	23.1
Student	5	19.2
Unemployed	1	3.8

a UK Office for National Statistics definitions of ‘urban’ and ‘rural’ area were used, based on Census 2021 data.

b A combined household income of less than £26,000 per year, before tax deductions.

The focus groups were recorded in Microsoft Teams and transcribed verbatim, anonymized, and then coded using deductive and inductive approaches to identify key themes in participants’ responses (i.e., *a priori* codes based on the focus group questions, as well as emergent codes to reflect unanticipated themes). Thematic Analysis was used to identify, analyze, and report patterns (themes) within the qualitative data (Braun and Clarke, 2006). The themes were coded using Lumivero NVivo software. In terms of the researcher’s positionality, I stated that I did not work for Cornwall Council, but was working with the Council to better understand the views of people who live in Cornwall. I encouraged the participants to express their opinions freely and emphasized there were no right or wrong answers.

### 2.2 Study 2—Behavior change intervention

**Figure 1:**
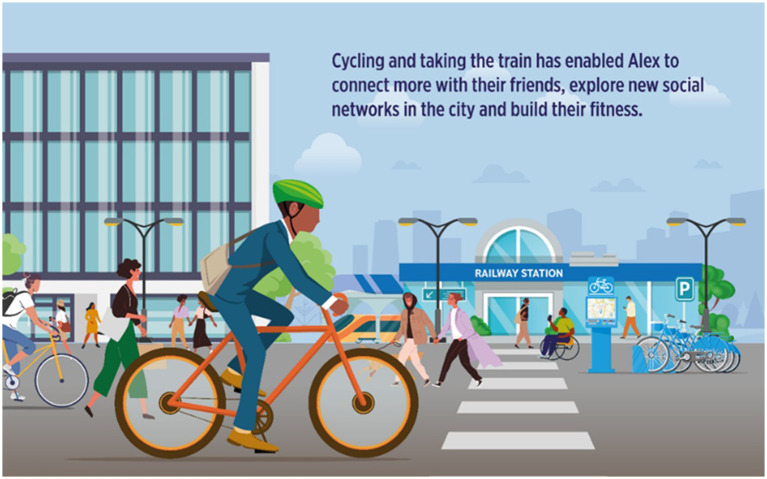
Pen portrait 2—a young adult living in an urban area in Cornwall.

The intervention study was conducted between May and July 2023. Two interventions were trialed to investigate their impact on the perceptions and travel behaviors of residents and Council employees. These interventions were selected by the researchers and Council partner based on the findings of Study 1 and on relevant empirical evidence and theory (particularly technology adoption theory and the COM-B model) that pointed to the importance of addressing individual, societal and technological factors:

1. Free credits to use Beryl bikes for 1 month. This intervention removed cost as an initial barrier and gave people direct experience of using shared e-bikes on a trial basis. This ‘trial period’ approach is consistent with previous intervention studies which provided short-term e-bike loans to encourage the subsequent purchase of an e-bike (Fyhri et al., 2017; Moser et al., 2018; Ton and Duives, 2021).
2. The ‘Pen portraits’ visioning tool (Prosser et al., 2022), adapted for the Cornish context. This tool presented stories of six evidence-based characters who have successfully reduced their car use and the study participants selected the character they most identify with. The intervention encouraged people to consider how they could reduce car use in their daily lives and highlighted potential co-benefits and positive lifestyle outcomes. Figure 1 is an example of a pen portrait; the image is accompanied by a one-page narrative about the depicted character. The visioning tool is available in Supplementary material.

As with the focus groups, residents were recruited via Council communication channels. The participants were not given any information about the interventions prior to the study, but the recruitment material highlighted four inclusion criteria: (1) to have an interest in active modes of travel (to ensure participants would be physically fit enough to take part); (2) to own a smartphone (which is required to unlock and use a Beryl bike); (3) to live, work or study in one of the six towns in Cornwall where Beryl bikes are available; and (4) be aged 16 or over. Participation was incentivized through a £25 gift voucher for each resident who completed the study. Council staff were recruited through Beryl communication channels when they registered for an internal Council promotion of free Beryl bike credits. Staff participation in the study was incentivized through entry into a prize draw.

A 2 × 2 factorial design was used, whereby residents (*n* = 200) were randomly allocated to one of four interventions groups: (A) a control group; (B) the visioning tool only; (C) Beryl bike credits only; and (D) Beryl bike credits plus the visioning tool. Of the 200 residents who started the study, 151 completed all of the data collection activities listed below and were included in the final data set. Table 2 shows the number of participants in each group that completed the study. Council employees who registered for the free Beryl bike credits parallel intervention group C, but they were not randomly allocated to an intervention group. Of the 27 Council employees who chose to participate, 14 completed the study.

**Table 2:** Study design—the four intervention groups.

	Visioning tool: NO	Visioning tool: YES
e-bike credits: NO	A. control group (*n* = 44)	B. visioning tool only (*n* = 36)
e-bike credits: YES	C. e-bike credits only (*n* = 38) Parallel study with Council staff (*n* = 14)	D. e-bike credits + visioning tool (*n* = 33)

The participants were asked to complete the following data collection activities:

1. A pre-intervention survey to measure current travel behaviors, perceptions of active travel and e-bike share, and sociodemographic characteristics. This survey highlighted the availability of Beryl bikes in their local area.
2. A weekly travel diary for four consecutive weeks to measure Beryl bike journey frequency, distance, purpose, and mode shift. Aggregated, observed travel data from Beryl, the company which operates the shared e-bikes, provided further insights on journey frequency and distance.
3. A post-intervention survey to measure any changes in perceptions or travel behaviors, and users’ satisfaction with Beryl bikes.

These data collection tools (see Supplementary material) were developed by adapting questions from previous travel behavior studies (CoMoUK, 2022; Wilson and Whitmarsh, 2023) or, where precedent questions were unavailable, bespoke questions were designed to explore themes of interest.

For research aim 1, participants’ perceptions of 15 attributes of Beryl bikes were measured to understand which aspects of e-bike share may appeal to them. The attributes were selected by incorporating findings from the focus groups and duplicating some of the attributes measured in the CoMoUK survey (2022). Participants were asked to indicate their level of agreement, on a five-point Likert scale, that using Beryl bikes would help them personally by providing these benefits. Their perceptions were measured in the pre- and post-intervention surveys to explore whether direct experience of using the bikes may influence their views.

For research aim 2, participants were presented with nine potential barriers in the post-intervention survey and asked which are the three main barriers to people using Beryl bikes in Cornwall. Participants also provided qualitative feedback on using e-bike share. This data was combined with travel diary responses on journey purpose, distance and mode shift to understand how e-bike share is used in rural settings.

Research aim 3 was to quantify the environmental impact of shared e-bike journeys replacing car journeys. This was achieved through a Life Cycle Assessment synthesis, an established approach for estimating the emission reduction associated with the adoption of a low-carbon behavior (Ivanova et al., 2020; Wilson et al., 2020). Quantitative estimates of the emission intensity of (1) car journeys and (2) e-bike or shared e-bike journeys were compiled from existing Life Cycle Assessment studies. These estimates produced a range of grams of CO_2_ per km (or grams of CO_2_-equivalent per km) for the two travel modes. Empirical data collected during this study, on participants’ shared e-bike journey frequency, distance, and mode shift, was situated within these emission intensity ranges. The emission reduction of shared e-bike substitution of car journeys was calculated *per person per journey*, and *per person per year*. The full calculations are presented in Supplementary material.

For research aim 4, the success of the interventions was determined by a dichotomous dependent variable: to have used OR not used a Beryl bike during the study period. Participants who received the visioning tool intervention (groups B and D) were asked three additional questions about the relevance of the tool to their lives and the potential impact of the tool on their travel behavior. A range of between-group and within-group statistical tests were used to measure differences in behaviors and attitudes: independent-samples t-test, Welch t-test, paired samples t-test, Chi-square test of homogeneity, and Fisher’s exact test. The statistical analysis was conducted using IBM SPSS software.

Table 3 is an overview of the intervention participants’ sociodemographic characteristics. Compared to Census 2021 data, this sample of residents is broadly representative of Cornwall’s population for age, ethnicity, low-income groups, and health condition or disability. However, it differs for gender, education, employment status, household composition and location (women, people with an undergraduate or postgraduate degree, those who are economically active, families with dependent children, and those who live in an urban area are all overrepresented in this sample).^1^ In terms of available travel options, the majority of residents in this study (86.1%) own a vehicle and almost half (48.3%) have two or more vehicles in their household. Similarly, most Council staff (92.9%) own a vehicle and over three quarters (76.9%) have two or more vehicles in their household. The study samples therefore have higher levels of vehicle ownership than the England average (78%), as well as higher levels of multiple vehicle households than the England average (33%) (Department for Transport 2022). This likely reflects a greater dependency on cars in rural areas. Owning a bicycle is another factor which could influence an individual’s uptake of e-bike share. Most Council staff (85.7%) and just over half of residents (53.6%) own a bicycle or e-bike that is in good working order.

**Table 3:** Sociodemographic characteristics of intervention study participants.

Sociodemographic characteristic	Residents (*n* = 151)		Council staff (*n* = 14)	
	Frequency	%	Frequency	%
Gender				
Female	90	59.6	8	57.1
Male	56	37.1	5	35.7
Non-binary	3	2.0	1	7.1
Ethnicity				
White British/White Cornish	138	91.4	14	100.0
Asian/Asian British	3	2.0	-	-
Mixed/Multiple ethnic groups	1	0.7	-	-
Other ethnic group	5	3.3	-	-
Have a longstanding health condition or disability	25	16.6	2	14.3
Have children (under 18) living at home	52	34.4	9	69.2
Live in an urban area^a^	72	47.7	7	50.0
Have an undergraduate or postgraduate degree	94	62.3	10	71.4
Employment status				
Employed (full- or part-time) or self-employed	116	76.8	14	100.0
Retired	17	11.3	-	-
Looking after family/ home	7	4.6	-	-
Student	4	2.6	-	-
Unemployed	1	0.7	-	-
Live in a low-income household^b^	35	23.2	1	7.1
Own a bike or e-bike in good working order	81	53.6	12	85.7
Own or have regular access to a car	130	86.1	13	92.9
Mean age (in years)	46.5 years		41.1 years	

a UK Office for National Statistics definitions of ‘urban’ and ‘rural’ area were used, based on Census 2021 data.

b A combined household income of less than £26,000 per year, before tax deductions.

## 3 Results

### 3.1 Study 1—Focus groups

The focus groups explored residents’ perceptions on a wide range of travel-related themes and only the findings that are most relevant to the design of the intervention study are presented here. Several participants described their dependency on cars because they live in rural areas and must travel long distances to get to work or access local services (*n* = 15): *“My work is 35 miles away, so unfortunately that is a no go on public transport.”* Additional determinants of car use include convenience (*n* = 6) and the need to transport shopping or passengers (*n* = 5): *“I’ll collect all my clients in my car…and take them to the activity.”* Many did not consider public transport viable due to a lack of services or poor connectivity in their area (*n* = 16): *“The last bus back is 20 [minutes] to 6 at night…so you can’t go out in the evening using public transport at all.”* A further barrier was the infrequency and unreliability of services (*n* = 14): *“The other thing is waiting for buses that never come.”* Some use multimodal travel to avoid traffic congestion in towns and save money on parking fees (*n* = 9): *“It costs a fortune…to park. So I will drive…park my car, then cycle the rest of the way.”* In terms of policies to reduce car travel, participants reported a strong preference for ‘pull’ measures which make sustainable alternatives easier or cheaper, rather than push measures which restrict car use or make it more expensive.

#### 3.1.1 Benefits of active modes of travel

Most participants expressed an interest in using active travel more frequently and described multiple benefits. The most frequently mentioned was providing exercise and improving physical health (*n* = 7): *“If I don’t go cycling at least twice a week, my knees go all rubbish, you know? So, as an older person, it keeps me in tip-top condition.”* Improving wellbeing and mental health was equally important (*n* = 7): *“What do I enjoy about cycling now? It’s meditative…it’s for an enjoyment aspect. I enjoy the action of cycling.”* A related benefit was that using active modes provides an opportunity to be outside and experience being in nature, particularly for those whose job involves being indoors for much of the time (*n* = 5): *“It’s more pleasurable, I would say. If it’s not raining, you know, you get the air, you just feel more alive, don’t you? You can appreciate your surroundings, it’s a nice feeling.”* Some participants identified cost savings, relative to other travel modes (*n* = 4): *“It’s great that you get to work for free and you get fit while you’re doing it.”* Other reported benefits include socializing by cycling as a group (*n* = 2) and reducing carbon footprint from avoiding car use (*n* = 2). Journey purpose and distance were important determinants of travel mode choice. For short journeys, many participants preferred to use active modes (*n* = 11): *“If it’s less than three miles, I prefer walking. That’s how I like to get around.”*

#### 3.1.2 Barriers to using active modes of travel

Despite these perceived benefits, residents identified multiple barriers to using active modes in Cornwall, especially for cycling. The most prevalent concern was safety when sharing the road with vehicles, particularly on narrow country roads (*n* = 19): *“The two miles between my house and Saltash where the cycle lane starts, it’s just deadly. So it’s not something that I’m even considering doing.”* A related barrier was the lack of cycle lanes and walking paths in rural areas (*n* = 15): *“There’s just a lack of infrastructure until you get somewhere close to a kind of urban area, then you have cycle paths and things like that. And even then, it’s pretty limited.”* Where cycles lanes are available, some participants described the routes as poorly maintained (*n* = 4) or badly designed; for example, ending unexpectedly at a busy junction (*n* = 5). In the preference ranking exercise, investment in active travel infrastructure was the second most popular ‘pull’ policy, after investment in public transport.

Participants also highlighted other barriers such as wet and windy weather during the winter months (*n* = 9) and the topography in Cornwall which is characterized by steep hills (*n* = 7): *“I think it’s too steep to cycle around here. Yeah, I don’t think I’ve got my bike out since, it’s exhausting!”* A lack of secure bike storage in town centers and workplaces was another challenge (*n* = 6): *“There aren’t many specific bike racks that I see, so we’ll just lock them to a fence or something.”* This barrier was particularly emphasized by those who own expensive bicycles and were concerned about theft. Other barriers included limited carrying capacity on bikes (*n* = 3), a lack of showering facilities at their place of work (*n* = 2) and experiencing air pollution when cycling (*n* = 2). Many of the barriers described above correspond with the findings of a previous study which investigated perceptions among the Council workforce (Player et al., 2022; Toy et al., 2023).

#### 3.1.3 Perceptions of e-bikes and e-bike share

One notable theme to emerge during the focus groups was residents’ views on e-bikes and how they counteract some of the physical barriers to using active modes. Six participants owned an e-bike and described how using one reduces journey time and mitigates the difficulty of cycling up steep hills (*n* = 6): *“Over the hills are too much for an ordinary bicycle. Electric bike makes all the difference.”* One respondent described how using his e-bike provides more personal comfort, as it enables him to arrive at work without being sweaty and fatigued. Participants were also generally positive about e-bike share schemes (*n* = 4): *“The e-bikes around cities, yeah, I think it’s a good idea. Obviously, it’s not relevant for us living in more rural communities. There’s only been two or three places I should think in Cornwall that it would work.”* That e-bike share was considered suitable only for urban areas relates primarily to road safety concerns, but also the view that the financial investment required to launch the scheme was not justifiable for smaller towns where most amenities are within walking distance (*n* = 2).

#### 3.1.4 Intervention design

When co-designing the behavior change intervention, Cornwall Council had a particular interest in encouraging and enabling more people to use active modes of travel, due to its potential to reduce carbon emissions as well as provide the health and wellbeing benefits described by the focus group participants. Addressing the most important barriers, road safety concerns and a lack of active travel infrastructure, requires long-term regional planning and significant fiscal investment, and so were beyond the scope of this pilot study. The decision to focus on the recently introduced e-bike share scheme was due to its potential to overcome two of the other barriers; shared e-bikes provide assisted power to cycle up steep hills, and they remove apprehension about the lack of secure bike storage. Moreover, using e-bike share aligns with the participants’ positive perceptions of using active modes, particularly for shorter journeys. Given the rural context, the Council were also interested in whether people would combine e-bike share with other travel modes for longer journeys.

### 3.2 Study 2—Intervention study

#### 3.2.1 Perceptions of e-bike share

**Figure 2:**
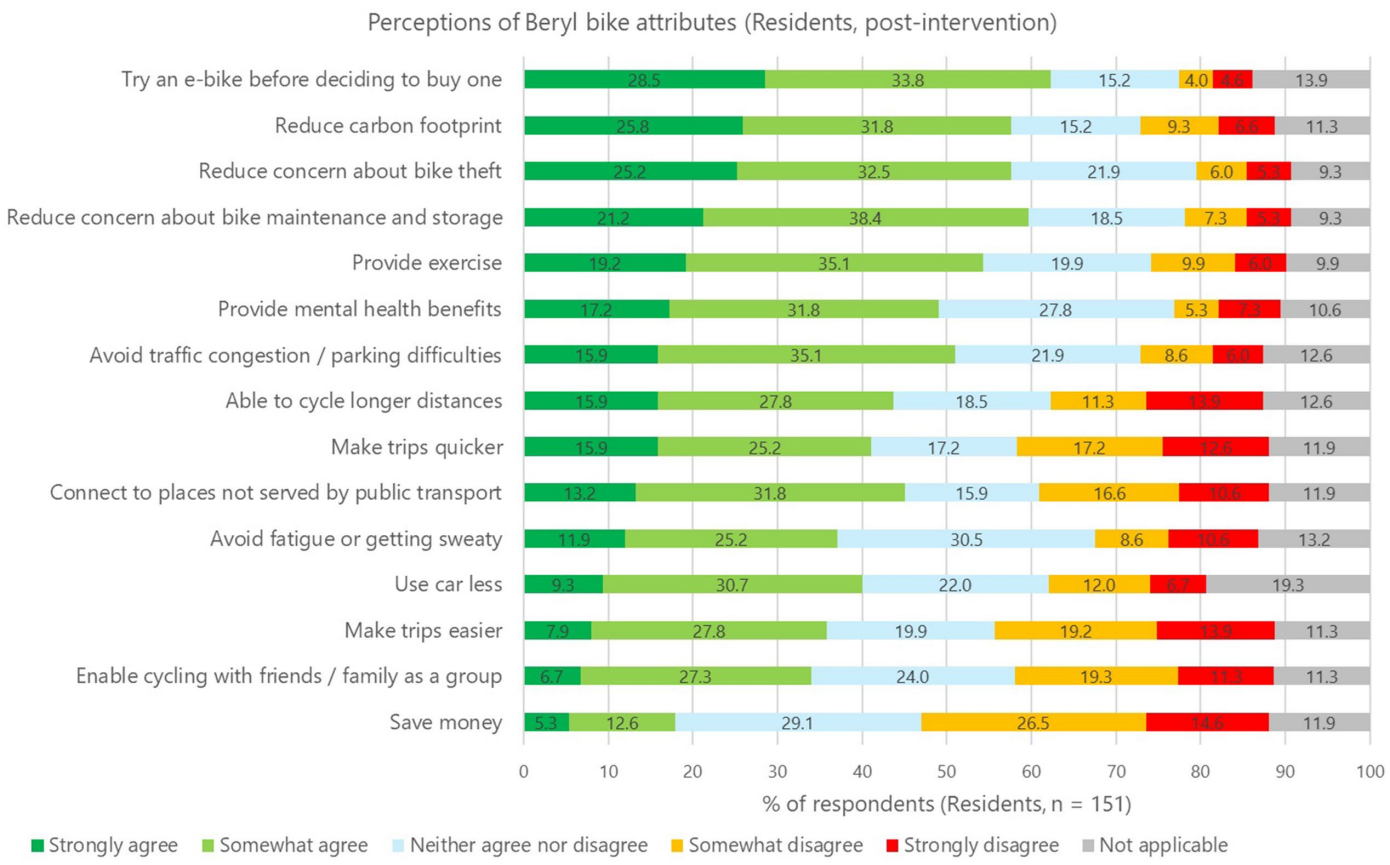
Residents’ perceptions of Beryl bike attributes (post-intervention).

This study measured participants’ perceptions of 15 attributes of Beryl bikes to understand which aspects of e-bike share may appeal to them. Although residents tended to rank the attributes slightly higher in the post-intervention survey, none of the differences are statistically significant (paired samples t-tests, see Supplementary material) and so there is no clear evidence of a change in their perceptions during the study period. Post-intervention survey findings for residents (*n* = 151) are presented in Figure 2, with the highest ranked attributes located at the top of the chart. Figure 2 shows practical attributes are important, for example, *trying an e-bike before buying one* is the highest ranked attribute, with 62.3% of residents stating they ‘somewhat agree’ or ‘strongly agree’ that Beryl bikes provides this benefit (i.e., the green segments in each bar). Moreover, there is broad agreement that using Beryl bikes *reduces concerns around maintaining a bike* (59.6%) and *bike theft* (57.7%), and *avoids traffic congestion and parking difficulties* (51.0%). Co-benefits are also important; 57.6% of residents believe that using Beryl bikes will *reduce their carbon footprint,* and there is strong agreement that Beryl bikes provide *exercise* (54.3%) and *mental health benefits* (49.0%). The lowest ranked attribute is *cost* (17.9%), as Beryl bikes are considered expensive to use. Council staff reported stronger agreement than residents for all attributes, although the small sample size for Council staff limits our ability to make comparisons between the two groups. The post-intervention findings for Council staff can be found in Supplementary material.

There are personal factors which may influence the adoption of e-bike share, even if participants have a positive perception of the scheme. As discussed earlier, the participants reported high levels of vehicle and bicycle ownership and so have multiple existing travel options. Interestingly, a high proportion of residents (60.9%) and Council staff (78.6%) indicated that they would like to reduce their vehicle use. Moreover, the majority of residents (70.2%) and Council staff (92.9%) are confident in their ability to ride an e-bike, although one in seven residents (13.9%) stated that walking or cycling as a main mode of travel is not feasible due to a longstanding health condition or disability. E-bikes are relatively expensive and two-thirds of residents (66.2%) expressed concerns that if they owned one, they would worry about it getting stolen. This may explain the high ranking of *reduce concern of bike theft* as one important attribute of e-bike share. Overall, these personal factors support the uptake of e-bike share.

Table 4 presents the findings of between-group analyses of residents’ perceptions of Beryl bikes. Those who used a Beryl bike during the study tended to rate the attributes higher than non-users, although only one difference is statistically significant; Beryl bike users reported stronger agreement that using the bikes would make their trips easier. People living in rural areas and those on lower incomes reported stronger agreement that using Beryl bikes would enable them to use their car less, and avoid traffic congestion and parking difficulties. People living in rural areas also reported stronger agreement that using Beryl bikes would connect them to places not served by public transport, enable them to try an e-bike before deciding whether to buy one, and provide exercise and mental health benefits. People on lower incomes reported stronger agreement that Beryl bikes would make their trips easier and avoid fatigue before work or socializing. There were no statistically significant differences in the level of agreement based on other grouping variables such as gender, education level, owning a bike, or driving intention (independent samples t-tests).

**Table 4:** Between-group analyses of residents’ perceptions of Beryl bikes (independent samples t-test).

Attribute of Beryl bikes	Grouping variable	M	SD	MD	df	*t*	*p*	95% CI	
								Low	High
Make trips easier	Beryl bike user	3.35	1.25	0.55	132	2.411	0.017	0.10	1.01
	Non-Beryl bike user	2.80	1.20						
Use car less^*^	Rural resident^a^	3.60	0.90	0.65	98	3.270	0.001	0.26	1.05
	Urban resident	2.95	1.24						
Avoid traffic congestion/parking difficulties	Rural resident	3.74	1.04	0.47	130	2.461	0.015	0.09	0.85
	Urban resident	3.27	1.14						
Try an e-bike before deciding to buy one	Rural resident	4.10	0.97	0.44	128	2.330	0.021	0.07	0.81
	Urban resident	3.66	1.17						
Provide exercise^*^	Rural resident	3.78	0.98	0.45	115	2.277	0.025	0.06	0.84
	Urban resident	3.33	1.27						
Provide mental health benefits	Rural resident	3.81	1.00	0.62	133	3.303	0.001	0.25	0.98
	Urban resident	3.19	1.16						
Connect to places not served by public transport	Rural resident	3.45	1.16	0.47	131	2.160	0.033	0.04	0.89
	Urban resident	2.98	1.34						
Use car less^*^	Low-income household^b^	3.88	0.77	0.77	64	3.876	0.001	0.37	1.16
	Higher income household	3.12	1.18						
Avoid traffic congestion/parking difficulties^*^	Low-income household	3.86	0.76	0.45	73	2.326	0.023	0.06	0.83
	Higher income household	3.41	1.22						
Make trips easier	Low-income household	3.41	1.21	0.55	121	2.106	0.037	0.03	1.07
	Higher income household	2.86	1.24						
Avoid fatigue or getting sweaty before work or socializing	Low-income household	3.64	1.16	0.53	118	2.130	0.035	0.04	1.03
	Higher income household	3.11	1.16						

a UK Office for National Statistics definitions of ‘urban’ and ‘rural’ area were used, based on Census 2021 data.

b A combined household income of less than £26,000 per year, before tax deductions.

* Levene’s test revealed unequal variances and so Welch t-test findings are reported, rather than the independent samples t-test findings.

#### 3.2.2 Uptake of Beryl bikes during the study

During the study, uptake of Beryl bikes increased from 6.6 to 30.5% for residents, and from 28.6 to 71.4% for Council employees.^2^ This relatively large increase suggests that participation in this study motivated some participants to adopt Beryl bikes, particularly Council staff. For residents, a higher proportion of bike owners (33.3%) than non-bike owners (27.1%) used a Beryl bike during this study, but the difference is not statistically significant (Fisher’s exact test, difference in proportions = 0.062, *p* = 0.479). Similarly, a higher proportion of car owners (32.3%) than non-car owners (19.0%) used a Beryl bike during this study, but the difference is not statistically significant (Fisher’s exact test, difference in proportions = 0.133, *p* = 0.308). Considering whether driving intention might be relevant to the uptake of Beryl bikes, a higher proportion of residents who are interested in reducing their car use rode a Beryl bike during this study (38.0%) than those who are not interested in reducing their car use (22.2%). However, this difference is not statistically significant (Fisher’s exact test, difference in proportions = 0.158, *p* = 0.168). The sociodemographic characteristics of Beryl bike users resemble the broader study sample: a relatively young mean age, although 13.0% of Beryl users are aged 60 or over; a minority have a longstanding health condition; and most own a bicycle and a car and so have multiple travel options. In summary, the adoption of e-bike share in Cornwall cannot be explained by bicycle or vehicle ownership, driving intention, nor by sociodemographic profile.

Beryl bikes were effective at re-engaging non-cyclists; hiring a bike encouraged one in six residents (14.7%) to try cycling again after a break of 5 years or more. In addition, approximately one in five residents (17.6%) and Council staff (20.0%) started cycling after a shorter break. This suggests that e-bike share schemes encourage the uptake of cycling among people who may have a lower propensity to use active modes.

#### 3.2.3 Journey purpose

**Figure 3:**
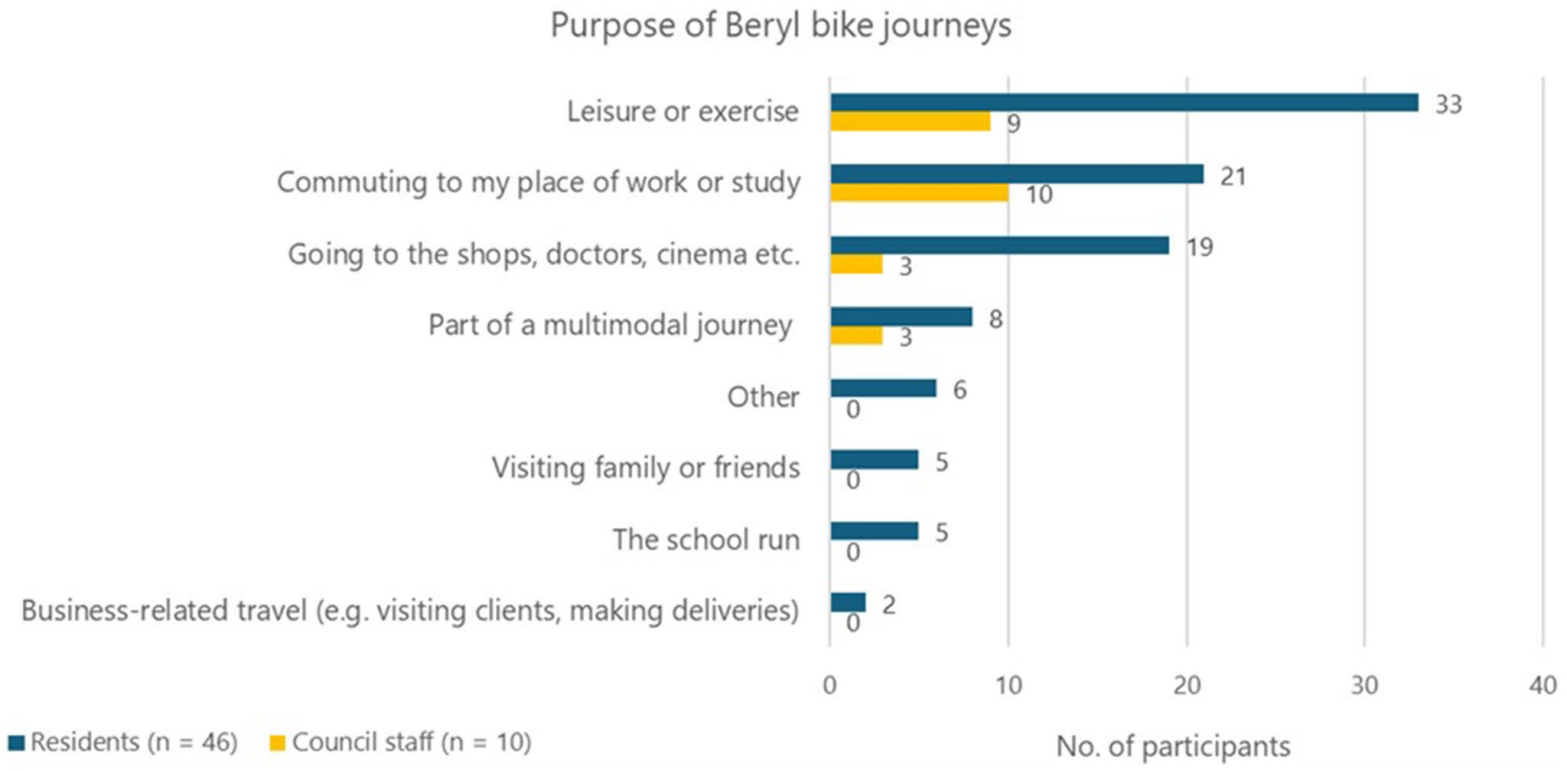
Purpose of Beryl bike journeys.

The purpose of the participants’ Beryl bike journeys was investigated to understand how using shared e-bikes may fit with their daily activities and travel needs. Anyone who used a Beryl bike was asked to indicate the purpose(s) of their Beryl bike journeys during that week (participants could select multiple options). Figure 3 shows that *leisure or exercise* and *commuting* were the most commonly reported journey purposes for both residents and Council staff. *Going to the shops* or accessing other local services was another common journey purpose, particularly among residents. In contrast to the frequency of commute journeys, *business-related travel (e.g., visiting clients)* was the least reported journey purpose. The data for the proximity of Beryl bike parking bays to the participants’ home or their place of work can be found in Supplementary material.

The focus groups revealed that many people in Cornwall live in rural areas and so require either a car or public transport to access towns and local services, and this is a clear barrier to shifting to active modes of travel. Cornwall Council were interested in whether e-bike share could be combined with other travel modes, whereby a Beryl bike is used for the segment of the journey within the town (i.e., the first or last mile). Figure 3 shows 11 participants used Beryl bikes as a component of multimodal travel. These participants were asked which travel modes they combined with Beryl bikes; *car* (as a driver and as a passenger) and *bus* were the most common responses (Table 5).

**Table 5:** Travel modes combined with Beryl bikes in multimodal journeys.

Mode of travel combined with Beryl bike	Residents’ (*n* = 8) no. of multimodal journeys	Council staff’s (*n* = 3) no. of multimodal journeys
Car/van as a driver	5	3
Bus	5	0
Car/van as a passenger	4	2
Train	2	2
Other	3	0
Taxi	0	0
E-scooter/scooter/motorcycle	0	0

#### 3.2.4 Journey distance, frequency and mode shift

This study explored the extent of Beryl bike use by asking participants to record their journey distances, frequency and mode shift in the travel dairies. Aggregated, observed data on journey frequency and distance was also available from Beryl, the service provider. Table 6 presents the mean distances for the two samples and two data sets, resulting in a range of 2.06–3.21 km per journey (or 1.28–1.99 miles per journey).^3^ Beryl bikes are therefore used primarily for short journeys, although longer journeys of 4 or 5 km were not uncommon. For residents and Council staff, the reported distances are slightly longer than the observed distances from Beryl bike data, although the two data sets are not directly comparable because they comprise different sample sizes and study period durations. Nevertheless, the range of 2.06–3.21 km per journey provides a useful indication, and is consistent with shared e-bike journey distances identified in previous studies (Fukushige et al., 2021; Zhou et al., 2023). A further finding is the relatively infrequent use of Beryl bikes during study period, with the mean number of journeys ranging from 0.8 to 1.5 per rider per week. This suggests many participants were trialing Beryl bikes and that using e-bike share was not yet embedded in their daily travel routine.

**Table 6:** Mean number of journeys and mean distance per journey.

Group	Mean no. journeys per rider over the study period	Mean no. journeys per rider per week	Mean distance (km) per journey
Residents (reported data, *n* = 41, over 4 weeks)	3.2	0.8	3.21^a^
Residents (observed data, *n* = 34, over 10 weeks)	8.5	0.8	2.99
Council (reported data, *n* = 10, over 4 weeks)	6.0	1.5	2.43
Council (observed data, *n* = 9, over 10 weeks)	9.1	0.9	2.06

a Five outliers were removed, because they reported very long journey distances, relative to the rest of the resident sample and to the Council staff sample. Thus, the residents’ reported journey distances in Table 6 may be an underestimate.

**Figure 4:**
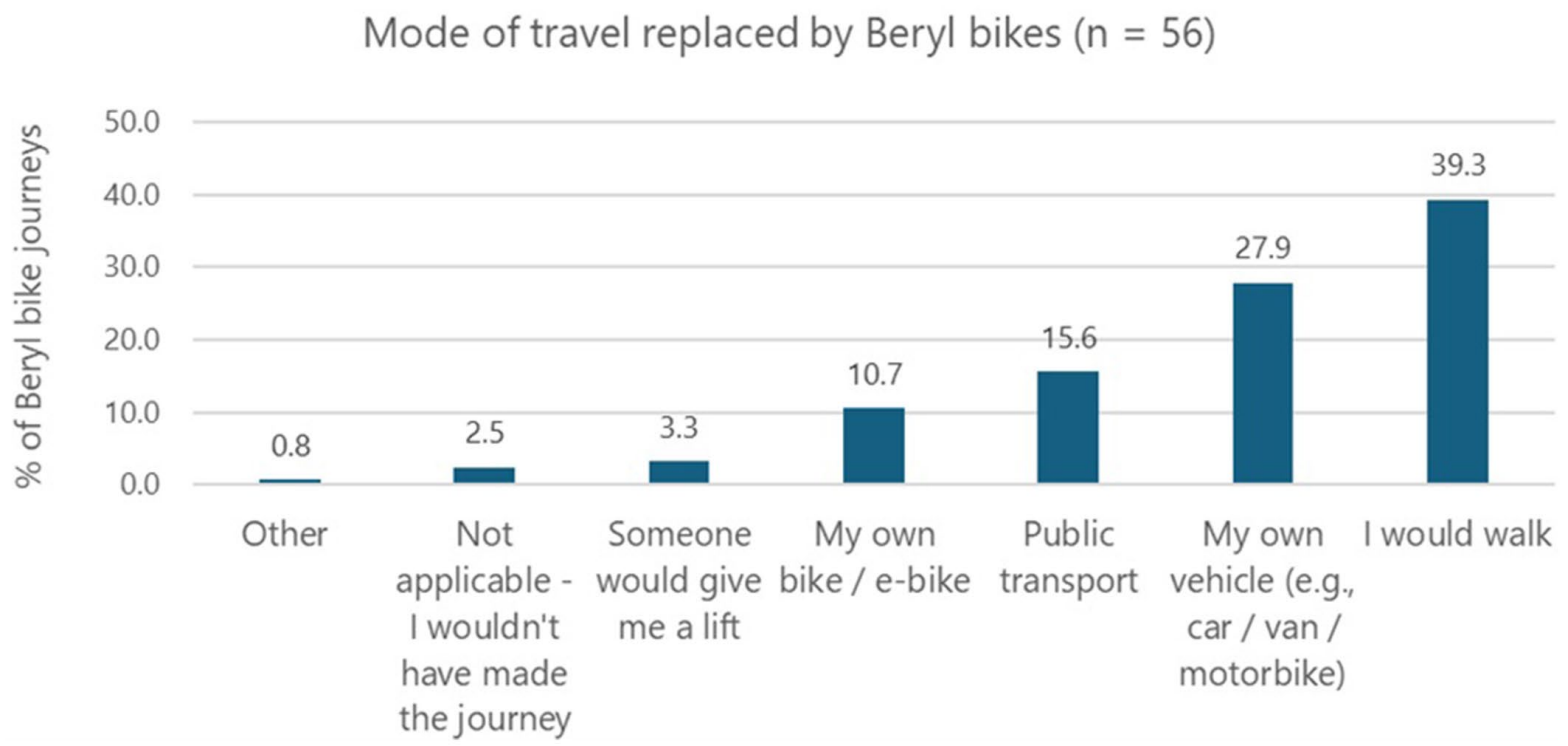
Mode of travel replaced by Beryl bike.

Participants were asked which mode of transport they would have typically used for their journey(s) before they started using the e-bike share scheme. Figure 4 reveals *walking* (39.3%) and *using my own vehicle* (27.9%) were the most commonly substituted travel modes (for residents and Council staff combined). However, the adoption of Beryl bikes did not reduce the overall time spent walking or wheeling each week (see Supplementary material).

#### 3.2.5 Carbon emission reduction

A Life Cycle Assessment synthesis was conducted to quantify the carbon emission reduction of e-bike share substitution of car journeys. Emission reduction ranges are provided below, rather than a single emission reduction figure, due to variation in the quantitative estimates and system boundaries of the Life Cycle Assessments, as well as variation in how residents and Council staff used Beryl bikes. For the participants in this study, the estimated emission reduction was 96–626 g CO_2_e per journey. This is a significant emission saving for a single journey. The annual carbon emission reduction due to mode shift from cars to shared e-bikes is 1.1–13.6 kg CO_2_e per person per year. For context, 13.6 kg CO_2_e per person per year is equivalent to 1.2% of the annual road and rail travel carbon footprint of a Cornwall resident (Cornwall Council, 2022). Thus, the annual emission reduction is modest and this is due to the relative infrequency of Beryl bike journeys made by the participants in this study, and that a significant proportion of e-bike share journeys substitute for walking rather than car use. The complete emission reduction calculations are presented in Supplementary material.

#### 3.2.6 Barriers to the uptake of Beryl bikes

Table 7 shows road safety concerns was the most important barrier, reflecting the findings of Study 1. Other key barriers are cost and the location of the parking bays.

**Table 7:** Barriers to the uptake of Beryl bikes.

Barrier	Residents (*n* = 150)		Council staff (*n* = 13)	
	Frequency	%	Frequency	%
Personal safety/busy roads/lack of safe cycling routes	111	74.0	8	61.5
Cost of using Beryl bikes	82	54.7	8	61.5
Location of parking bays	56	37.3	7	53.8
Lack of cycling confidence or competence	56	37.3	3	23.1
Long distances/steep hills	51	34.0	2	15.4
Lack of availability of bikes in parking bays	37	24.7	6	46.2
Lack of awareness about Beryl bikes	35	23.3	2	15.4
Beryl bike reliability/battery charge	16	10.7	1	7.7
Beryl bike design/comfort	6	4.0	2	15.4

Participants’ qualitative feedback revealed other reasons for not using a Beryl bike. For instance, a Beryl bike may not be suitable for particular journey purposes such as when people need to transport passengers or bulky items (*n* = 5), or do a big food shop (*n* = 7): *“I needed more storage space to carry my groceries home.”* For work-related travel, some participants were uncertain if they could cycle to their destination on time (*n* = 3): “*All required journeys this week were…time sensitive.”* Suggestions for improving the scheme included allowing a short pause in the journey without being required to park the bike in a bay (*n* = 5), providing a helmet with the bike (*n* = 7), and catering for families by adding child seats or providing smaller e-bikes (*n* = 9). These insights reveal that specific daily activities and responsibilities can play an important role in people’s choice of travel mode, even if they support using e-bike share.

#### 3.2.7 Behavior change intervention results

**Figure 5:**
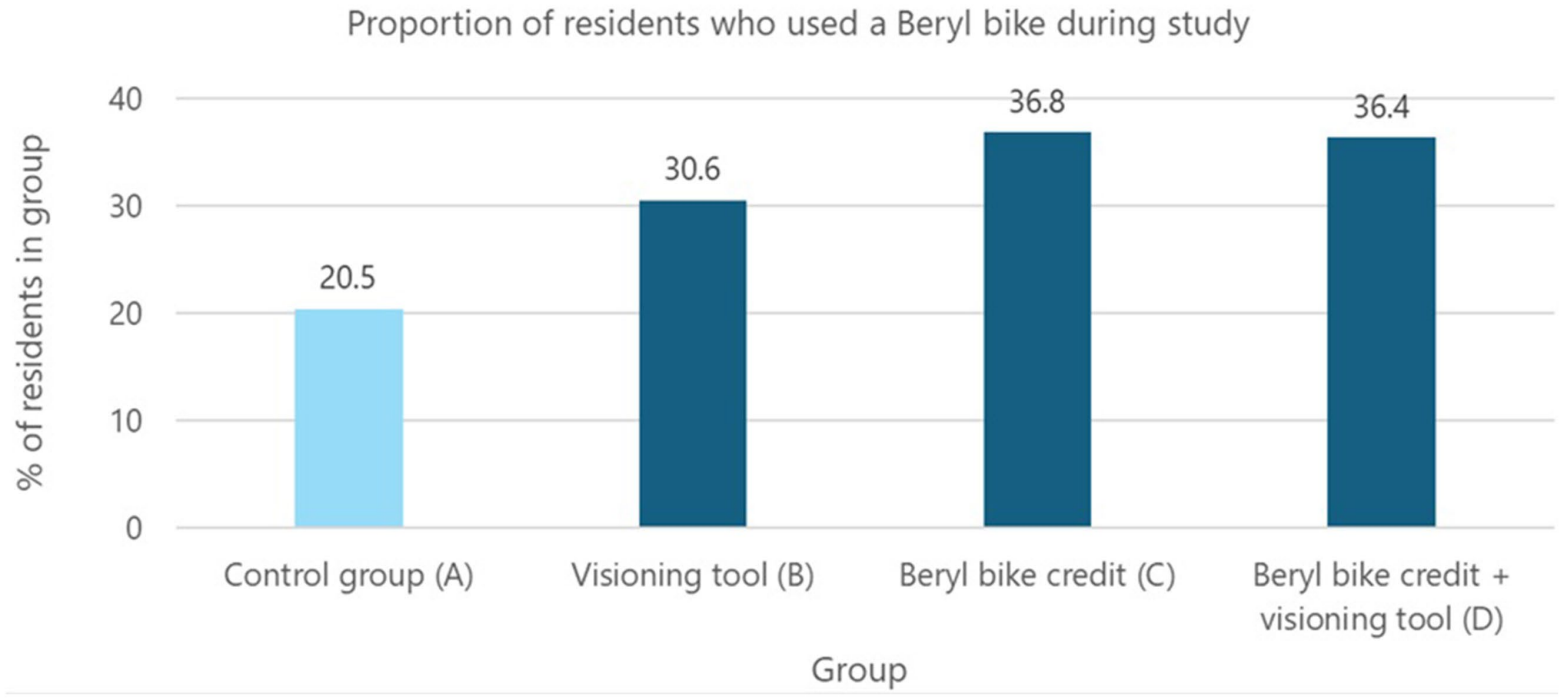
Proportion of each group that used a Beryl bike during the study.

Figure 5 indicates that the first intervention, free credits to use Beryl bikes for 1 month, was more effective than the second intervention, the pen portraits visioning tool, for encouraging uptake of e-bike share. Relative to the control group, a greater proportion of participants in each of the three intervention groups used a Beryl bike during the study period. Figure 5 shows the differences in the proportions are quite large, ranging from 10.1 to 16.3% greater than the control group. However, a Chi-square test of homogeneity revealed no statistically significant difference in the uptake of Beryl bikes between the control group and the three intervention groups: *X^2^*(df = 3, *N* = 151) = 3.353, *p* = 0.340. The data was further explored by combining intervention groups in different configurations to boost sample sizes (e.g., combining groups C and D to compare with the control group), but again, no statistically significant differences were found (Fisher’s exact tests^4^). This lack of statistical significance is likely due to the low statistical power of relatively small sample sizes in each intervention group. The implications of small sample sizes when conducting behavior change intervention studies is considered in the Discussion.

The visioning tool intervention had a broader focus than providing free Beryl bike credits, as it encouraged participants to consider multiple ways they could travel more sustainably, not only shared e-bikes. The visioning tool was presented to residents that were allocated to Groups B and D and they were asked, “Please consider your personal situation…where you live in Cornwall, your job, your family commitments, your transport needs. Then choose the character which you think might be the closest to your situation.” Table 8 shows which characters the participants selected; *older couple living in a rural area* and *middle-income parents* were the most common.

**Table 8:** ‘Pen portrait’ characters selected by recipients of the visioning tool.

Pen portrait characters	Residents (from Groups B & D)	
	Frequency^a^	%
An older couple living in a rural area	21	33.9
Middle-income parents	20	32.3
A young adult living in an urban area	15	24.2
A small business owner	4	6.5
A single parent on lower income	2	3.2
A young adult who uses a wheelchair	0	0.0

a Due to missing data for the questions which evaluated the pen portraits tool, the number of participants is slightly lower: *n* = 62 (rather than the 69 participants in Groups B & D that completed the remaining data collection activities in this study).

**Figure 6:**
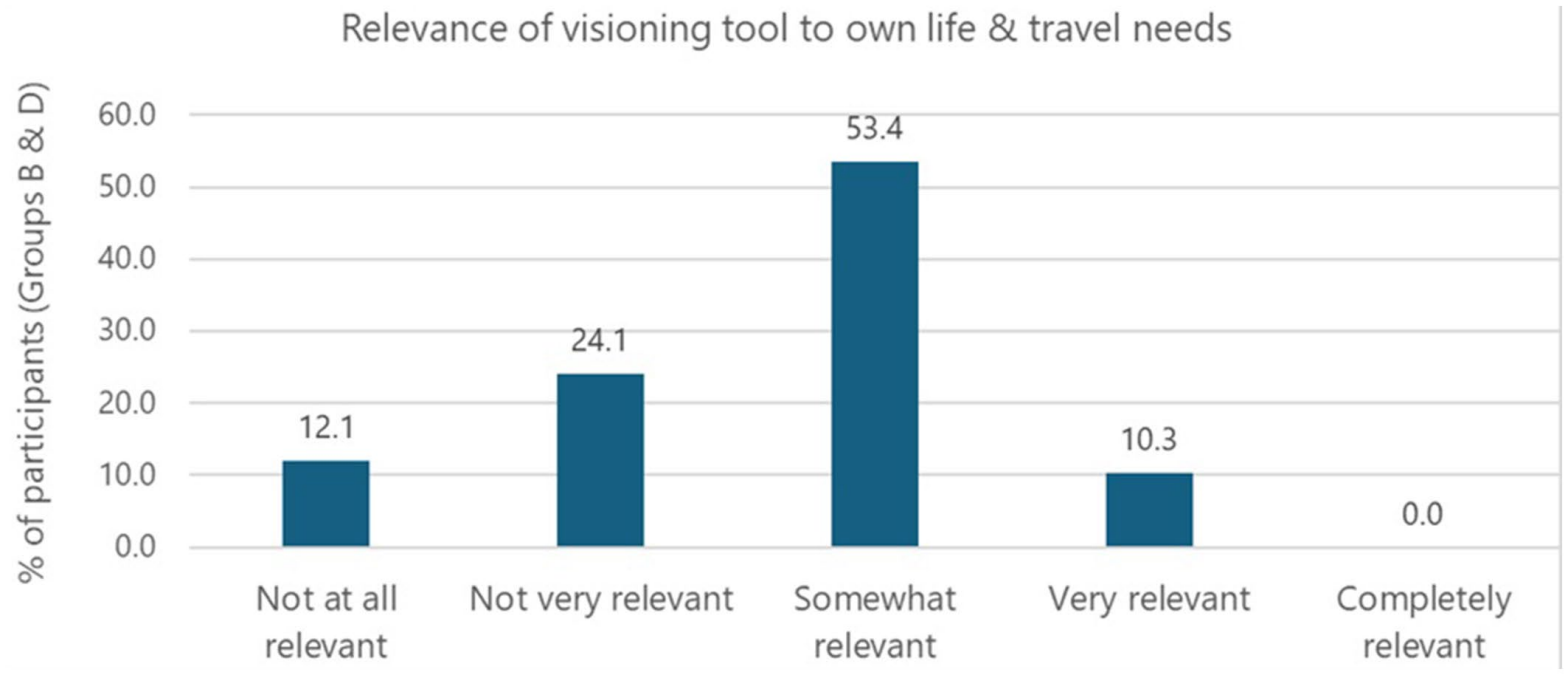
Perceived relevance of the visioning tool to participants’ lives and travel needs.

After reading the character’s story, participants were asked to reflect on whether they found the story relevant to their own lives and travel needs. Figure 6 shows over half (53.4%) believe the story is *‘somewhat relevant’*. The mean score for perceived relevance was 2.62 which, for comparison, is lower than the study of Scottish residents using these pen portraits, where mean scores ranged from 3.03 to 3.73 (Prosser et al., 2022). A higher mean score represents greater perceived relevance.

**Figure 7:**
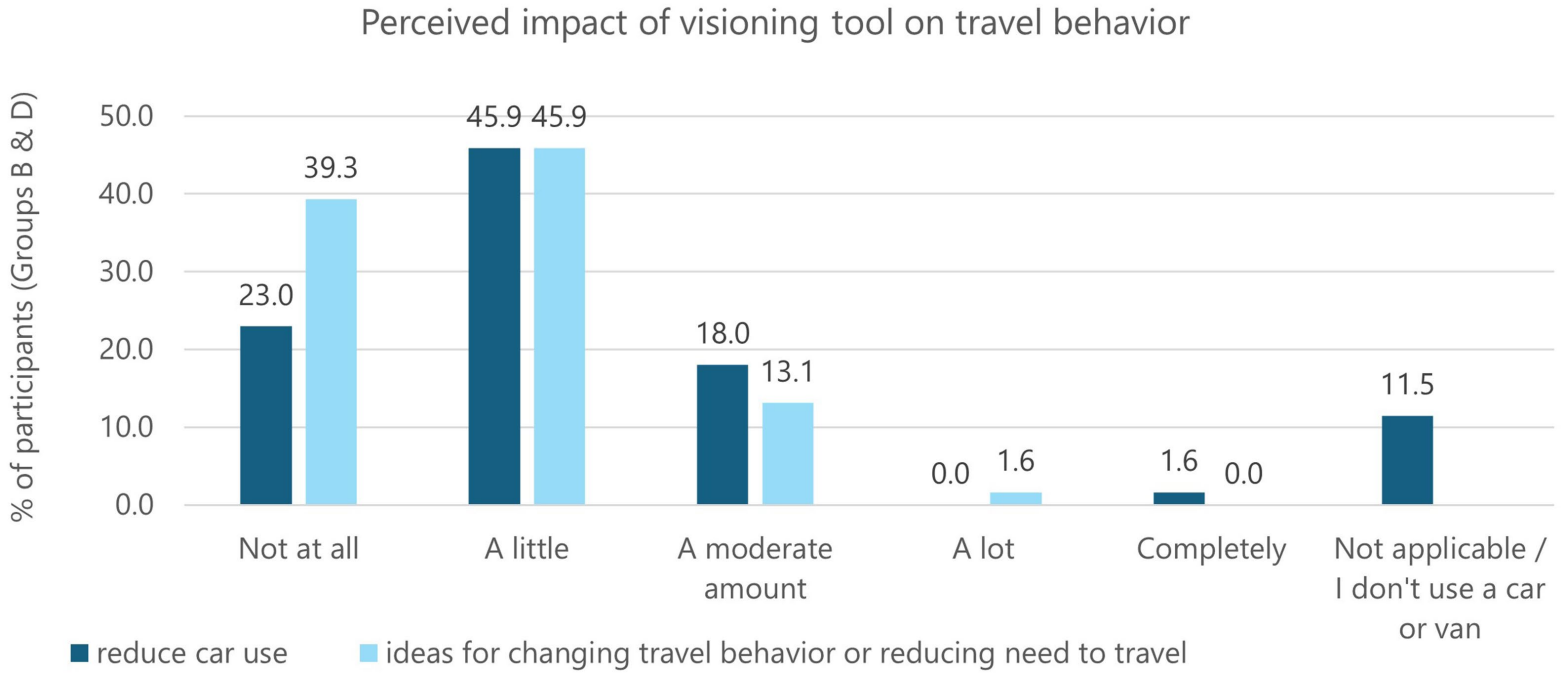
Perceived impact of the visioning tool on participants’ travel behaviors.

Figure 7 shows participants’ responses for the extent to which the scenario made them feel they could reduce their car use (the dark blue bars), and whether the story gave them ideas for how they might change how they travel in Cornwall or reduce their need to travel (the light blue bars). The most common response for both questions was *‘a little’* and this indicates the visioning tool did not have a significant impact on changing travel behaviors. The mean score for reducing car use was 2.00 which is somewhat lower than the study of Scottish residents, where mean scores ranged from 2.50 to 3.13 (Prosser et al., 2022). The mean score for generating ideas about changing travel behavior or reducing the need to travel was 1.77 (this question was not presented to Scottish residents). This low perceived impact could be because the participants did not find the visioning tool useful in terms of presenting novel ideas or highlighting possible lifestyle benefits of reducing car use. However, it could also reflect the structural barriers identified in Study 1, such as limited active travel infrastructure and public transport services, which constrain participants’ capacity to reduce their car use irrespective of whether they wish to.

## 4 Discussion

The discussion consists of two sections; the first considers the use of e-bike share in rural areas and the efficacy of the two interventions in encouraging mode shift, and the second examines some practical and analytical challenges for conducting behavior change interventions in real-world settings.

### 4.1 Use of e-bike share in rural settings

#### 4.1.1 Perceptions of e-bike share—research aim 1

The participants in this study were broadly positive about Beryl bikes. They perceive a range of practical benefits such as reducing concern around bike theft and avoiding parking difficulties, and this is consistent with previous research (Teixeira et al., 2023; Bartling, 2023). Co-benefits such as providing an opportunity for physical exercise, improving mental health, and reducing personal carbon footprint have received less attention in previous studies, aside from the CoMoUK annual survey (2022). Interestingly, participants in this Cornwall study and the CoMoUK survey ranked these co-benefits almost as highly as the practical benefits. This suggests e-bike share is viewed not only as a functional or convenient mode of travel, but also as a way of improving personal health and supporting the societal goal of tackling climate change. This ‘stacking’ of benefits can potentially tip the balance when one attribute, in this case the perceived high cost of using the shared e-bikes, is viewed less favorably.

Another interesting finding was that people living in rural areas and those on lower incomes tend to rank e-bike share attributes higher than people living in urban areas or on higher incomes. For example, rural residents believe that e-bike share would enable them to avoid traffic congestion and parking difficulties, which could reflect negative experiences of driving their car into busy town centers in Cornwall. Those on low incomes see Beryl bikes as an opportunity to use their car less, which could indicate concern about the rising cost of fuel. This variation in perceptions highlights that individual factors can affect the adoption of e-bike share (Barbour et al., 2019; Fukushige et al., 2021). Viewing these findings through a technology adoption theoretical lens, individual factors (e.g., income) and contextual factors (e.g., living in a rural area) are two key determinants that can influence the uptake of an innovation such as a new mode of travel (Straub, 2009; Whittle and Whitmarsh, 2022). Overall, these results indicate a positive potential for the roll out of e-bike share in rural areas.

#### 4.1.2 How e-bike share is used in Cornwall—research aim 2

The participants’ journey distances of 2.06–3.21 km per journey are comparable with those identified in previous e-bike share studies (Fukushige et al., 2021; Zhou et al., 2023). This affirms that e-bike share is primarily used for short journeys, irrespective of whether in an urban or rural context. For journey purposes, leisure or exercise and commuting were the most commonly reported journey purposes in this study. This is broadly similar to previous studies (Guidon et al., 2019; CoMoUK, 2022), although participants in this Cornwall study reported higher levels of leisure or exercise journeys. This could be because many were trying e-bike share for the first time and so chose to use it for casual or informal activities initially, as opposed to a necessary journey to work. In terms of mode shift, walking and using a car were the most commonly substituted travel modes. These findings are comparable with evidence of mode shift identified in other studies, although shared e-bike substitution for public transport is more prominent in areas with high population densities (Fukushige et al., 2021; Bieliński et al., 2021). Some participants combined e-bike share with other travel modes, whereby a Beryl bike is used for the segment of the journey within the town (i.e., the first or last mile). This finding is of particular interest to Cornwall Council, as reducing traffic congestion in towns has societal benefits such as reduced journey times and lower levels of air pollution. Beryl had positioned some of their docking stations near existing park and (bus) ride hubs, in the hope of enabling ‘park and e-bike ride’ as a multimodal travel option.

#### 4.1.3 Mode shift emission reduction—research aim 3

This study found shifting from single occupancy car to a shared e-bike results in a considerable emission saving of 96–626 g CO_2_e per journey, and this reflects the much lower emission intensity of e-bike share compared to ICE vehicles (Brand et al., 2022; Zhou et al., 2023; Li et al., 2023). However, significant emission reduction at the individual level is contingent on how frequently a pro-environmental behavior is performed (Nielsen et al., 2021). For daily travel behaviors, this means regularly choosing e-bike share (or another sustainable mode) over car for work, shopping, and leisure journeys.

The annual emission reduction due to mode shift in this study was modest, only 1.1–13.6 kg CO_2_e per person per year. Comparing this finding with other studies, CoMoUK (2022) estimates mode shift emission savings to be 71 kg CO_2_e per person per year (for bike share and e-bike share users combined), whereas a study of private e-bike users found a much higher reduction of 272–394 kg CO_2_e per person per year (Winslott Hiselius and Svensson, 2017). Although the e-bike journey distances in these two studies are comparable with this Cornwall study, the combined distance of multiple e-bike journeys over a week far exceeds the combined distance of only one or two e-bike journeys a week for the Cornwall participants. These large differences in annual emission reduction are therefore attributed to the relative infrequency of Beryl bike journeys made by the participants in this study, and that many journeys substituted for walking rather than car use. This infrequency of journeys should be viewed in the context that most participants were trying Beryl bikes for the first time and so using e-bike share was not yet embedded in their daily travel behavior. The uptake of bike/e-bike share in the UK and elsewhere would indicate that, for many people, using e-bike share does become habitual and a regularly used travel option (Galatoulas et al., 2020; CoMoUK, 2024).

The challenge is therefore twofold: how to design interventions which ensure the choice of a sustainable travel mode becomes routine or habitual, and how to target these innovations at frequent car users who have the capacity to shift modes for some of their journeys. Habitual behavior is elicited by specific cues in stable and recurrent performance contexts, such as commuting to work or going food shopping (Verplanken and Whitmarsh, 2021). Interventions which aim to break existing habits (e.g., car use) should be associated with a repeated activity, such as traveling to work, and be continued for a sufficiently long duration to allow the formation of new habits (e.g., using e-bike share). Moreover, the sustainable mode should be reliable, attractive and flexible so that it may become the default choice (Powell and James, 2023). A co-benefits framing could be used to target particular social groups, such as frequent car users, and for specific journey purposes (e.g., commuting).

#### 4.1.4 Effectiveness of the behavior change interventions—research aim 4

There was a relatively large increase in the uptake of Beryl bikes during this study, particularly among Cornwall Council employees. Residents and Council staff indicated a strong interest in reducing car travel and both groups reported positive perceptions of e-bike share attributes, which suggests the participants are interested in exploring more sustainable ways to travel. A particularly encouraging finding was that Beryl bikes were effective at re-engaging non-cyclists; hiring an e-bike encouraged one in three residents to try cycling again after a break. Moreover, the opportunity to try an e-bike before deciding whether to buy one was the highest ranked attribute in this study, and this may be especially important for those who have not cycled for a long period but are interested in trying it again. These findings align with key insights from technology adoption theory, that the compatibility of an innovation with an individual’s needs and/or lifestyle is a strong predictor of adoption (Straub, 2009; Whittle and Whitmarsh, 2022). The focus groups revealed another dimension of compatibility, that the assisted power of e-bikes could counteract personal barriers to using active modes such as health difficulties or low fitness levels, as well as some of the physical barriers such as the steep hills and long journey distances that typify travel in many rural areas. While some people will readily choose active modes for the perceived health and wellbeing benefits described in Study 1, others may be deterred by these personal and physical barriers. Engaging these ‘harder to reach’ social groups and providing mode shift options that are feasible and accessible to them is crucial for achieving equitable, low-carbon travel goals. This study indicates that e-bike share has an important role in making active travel more inclusive, in addition to the health and climate benefits (Fukushige et al., 2021; CoMoUK, 2022).

Despite these positive findings, the results on the efficacy of the two behavior change interventions are mixed. The primary measure of success of the interventions was the adoption of Beryl bikes. The observed differences between the control group and the intervention groups are relatively large, but not sufficiently large enough to produce statistically significant results. This would suggest a potential Type II error (i.e., not detecting a difference when one actually exists), although this is very difficult to ascertain. We can cautiously infer the interventions had a role in encouraging *some* participants to try e-bike share. A more definite finding is that the ‘free Beryl bike credits’ intervention was more effective in influencing the behavior of Council employees than residents, albeit with a much smaller sample size. This suggests the allocation of free credits may be more effective at motivating adoption of e-bike share among groups of individuals who make similar, frequent journeys (i.e., commuting or work-related travel), than for groups with more heterogenous journey destinations. This finding aligns with previous studies on e-bike adoption (Ton and Duives, 2021; Julio and Monzon, 2022). Moreover, large organizations have existing communication channels and managers who can influence behaviors and lead by example.

The second intervention was the pen portraits tool for encouraging people to consider how they could reduce car use in their daily lives. Over half of the participants believed the narrative was ‘somewhat relevant’ to their lives and travel needs. This finding indicates that presenting narratives of low-carbon travel and the potential co-benefits of reducing car use resonates with some people, but not all. The pen portraits visioning tool was developed by Prosser et al. (2022) and, to date, this is the only other study to have applied it. The lower mean scores for perceived relevance and reducing car use would suggest the tool was less effective at motivating behavior change among Cornwall residents than Scottish residents. This might be explained by the structural barriers to active travel in Cornwall identified in the focus groups. Although Scotland and Cornwall are comparable in that a large proportion of residents live in rural areas, Scotland also has large cities with well-developed active travel and public transport infrastructures. This highlights that the local context of where an intervention is implemented can be a significant determinant of its effectiveness. The importance of context in shaping perceptions was identified in other studies which used a personas-based approach (Cherry et al., 2022, 2024).

### 4.2 Conducting interventions in real-world settings

The second part of the discussion focuses on the core question of this collection, ‘How Do Behavior Science Interventions to Reduce Environmental Impacts Work in The Real World?’. Intervention studies conducted in real-world settings are much needed in the sustainable travel context (Graham-Rowe et al., 2011; Okraszewska et al., 2024), and are important for several reasons. They aim to demonstrate what works, but also what does not, potentially saving expenditure of public money on ineffective large-scale programs. Interventions applied in the context of daily lives can reveal unanticipated barriers to changing behaviors, and whether those barriers primarily relate to personal or structural constraints, which may become apparent only from practical experience (Grilli and Curtis, 2021). They can also reveal specific situations where the behavior change is easier or more difficult to implement, or which social groups found the intervention most useful or compatible with their existing routines. Variation among individuals in terms of their habits or their ability to adopt a particular low-carbon behavior can significantly affect the efficacy of the intervention (Nielsen et al., 2021; Whitmarsh et al., 2021). Finally, the study findings can be used as a tool for engaging people in future behavior change initiatives, by highlighting tangible benefits which the participants experienced that others may be able to relate to their own lives. Experiential findings are therefore more informative than reported perceptions of how an intervention *could* change behaviors in a hypothetical scenario.

#### 4.2.1 The importance of partnerships and co-design

One observation from this study and previous collaborative projects is that forming partnerships with key stakeholders and target groups is an important enabler for effective behavior change interventions (Mitev et al., 2023). This project entailed a collaboration with Cornwall Council, a unitary local authority, and Beryl, the shared e-bike provider. These organizations supported various aspects of the design and implementation of the study. For instance, they used their communication channels to recruit participants, they supplied the credits to use the Beryl bikes, and they provided access to anonymized travel data for the participants’ shared e-bike journeys. This partnership therefore considerably increased the scope and ambition of this intervention study, which mutually benefits all partners. Moreover, engaging in a co-design process ensures the intervention aligns with the partners’ priorities and provides the empirical data they need to create effective policies or programs for motivating behavior change. This study was also grounded in Cornwall residents’ needs and concerns, as elicited through the initial focus group stage. This ensured the intervention design targeted key drivers and barriers of modal shift, tailored to the unique (rural) context of Cornwall. Stakeholders such as local authorities and private sector service providers have the capacity to rapidly scale up successful interventions, with the potential to significantly reduce environmental impacts, as well as provide individual and societal co-benefits such as improved health. Strong partnerships and shared learnings from previous pilots are more likely to lead to scaling up, or indeed the trialing of other behavior change interventions. Finally, interventions delivered through partnerships provide replicable examples which can be adapted and implemented in comparable settings and locations elsewhere.

#### 4.2.2 Dropout rates and the cost of conducting intervention studies

One challenge of conducting longitudinal intervention studies in real-world settings is the risk of a high participant dropout rate. In this study, the dropout rate was 24% for residents and 48% for Council staff, despite generous incentivization and weekly email contact from the researcher to encourage completion of data collection activities (see Supplementary material—attrition rate over the intervention period). This high dropout rate is by no means unusual; attrition of 30–70% has been identified in previous longitudinal studies (de Leeuw and Lugtig, 2014; Verfuerth and Sanderson Bellamy, 2022). The main consequence of a high dropout rate is a small sample size, which can lead to low statistical power despite relatively large observed differences between the intervention groups, as in this study. Thus, interventions may appear to be effective, but the lack of statistical significance does not support making strong assertions of their efficacy to project partners such as local authorities. Moreover, negative results are less likely to be published in academic journals (known as ‘positive publication bias’) and this can lead to unnecessary duplication of research, or a distortion of meta-analysis findings (Mlinarić et al., 2017; Andrews and Kasy, 2019).

One approach to mitigating the impact of participant dropout is to use statistical methods to handle missing data, such as imputation (Yang and Maxwell, 2014; de Leeuw and Lugtig, 2014). However, substituting missing values can introduce additional sources of bias and therefore requires clear justification regarding the choice of imputation method (Ren et al., 2023). Another solution is to increase the number of participants to provide a larger sample size, accounting for the anticipated dropout. This solution would also allow us to control for variables known to influence outcome variables (e.g., income) in our analysis, something we were unable to do due to the small sample size. However, this solution encounters a further challenge, because longitudinal behavior change interventions can be expensive to conduct. This intervention study (excluding the initial focus group study and the intervention study with Council staff^5^) cost £3,775 in research participation incentives and £4,000 for the Beryl bike credits. This total of £7,775 does not include researcher or project partner time for co-designing and promoting the study, collecting and analyzing the data, and producing research outputs. Extrapolating from the observed differences between the intervention groups in this study, and assuming an identical dropout rate of 24%, an initial sample of *n* = 577 would be required to produce a statistically significant difference between the control group and intervention groups C and D.^6^ For this increased sample size, the intervention study would cost £26,196 (excluding researcher time). This cost would be prohibitive for many research projects, except those that are particularly well-funded. This ultimately raises a question of which measures should be used to determine if an intervention has proven successful, given that many local authorities, third sector climate organizations, and universities face funding constraints. A greater reliance on qualitative findings which report participants’ and service providers’ views on the positive and negative impacts of the intervention is one possible solution.

### 4.3 Limitations

This study had several limitations. The implications of participant dropout have been described above. Further limitations include a potential self-selection bias in the samples of residents and Council workforce. For residents, one of the inclusion criteria specified on the recruitment material was to have an interest in active modes of travel, and so this sample does not include those who have no such interest. Similarly, the Council employee sample may not be representative of the wider Council workforce, as these individuals chose to register for the promotion to receive free Beryl bike credits and so have a discernible interest in active travel. There is no claim that the two samples are representative of the wider populations of all Cornwall residents and Council staff, but it is hoped these samples would reflect the views and travel behaviors of people in Cornwall who are interested in using active modes. There is also a potential self-selection bias in terms of who chooses to participate in a research project, which often leads to an overrepresentation of women in the sample, as in this study. An additional potential bias relates to the use of Council communication channels for recruitment, as not all residents will have signed up to receive Council newsletters or regularly check the Council’s website and social media. Those who are less engaged in Council activities or less familiar with using digital platforms would therefore be unaware of the opportunity to take part. We also cannot rule out the possibility of social desirability bias or demand characteristics in the pen portrait intervention (i.e., reporting a more favorable impact of the intervention on their behavior to align with the study aims). Moreover, it was not possible to link observed data on participants’ use of Beryl bikes with the reported data from the surveys and travel diaries, due to Beryl’s data protection protocols. Beryl data was therefore aggregated and anonymized which, although still useful, increases the uncertainty range of the emission reduction calculations. The timing of the intervention study, conducted in May–July, may have affected the findings. The interventions would likely be less successful during winter months, when inclement weather would deter some people from using active modes. Suggestions for improving the study design include repeating the intervention during the winter and conducting a follow-up data collection activity to ascertain if the altered travel behaviors have continued after the intervention has finished.

## Data Availability

The raw data supporting the conclusions of this article will be made available by the authors without undue reservation.

## Footnotes

^1^See Census 2021: https://www.ons.gov.uk/visualisations/censusareachanges/E06000052/; https://www.ons.gov.uk/peoplepopulationandcommunity/educationandchildcare/bulletins/educationenglandandwales/census2021; https://assets.publishing.service.gov.uk/government/uploads/system/uploads/attachment_data/file/88141/cornwall-and-isles-of-scilly.pdf.

^2^A Fisher’s exact test revealed this difference in the proportions (0.409) of Council employees and residents who used a Beryl bike during this study is statistically significant, *p* = 0.005. However, one of the expected cell counts was less than 5 and this low sample size invalidates this finding (Hollander and Wolfe, 1999).

^3^A range is provided, rather than a single mean distance, because: (a) there are two participants groups in this study, residents and Council staff, and (b) two different data sets, the travel diaries (reported data) and Beryl data (observed data).

^4^Fisher’s exact tests revealed no statistically significant differences in the uptake of Beryl bikes between: (1) those who received free Beryl bike credits (groups C + D; 36.6%) and the control group (20.5%), difference in proportions = 0.161, *p* = 0.095; (2) those who received the visioning tool (groups B + D; 33.3%) and the control group (20.5%), difference in proportions = 0.128, *p* = 0.199; (3) those who received an intervention of any kind (groups B + C + D; 34.6%) and the control group (20.5%), difference in proportions = 0.141, *p* = 0.119.

^5^The Council staff Beryl bike intervention study cost a further £740, but this amount was not included in this calculation as the staff intervention differs from the resident intervention in terms of its research design.

^6^These calculations were made using the ClinCalc (https://clincalc.com/stats/samplesize.aspx), assuming (1) a dropout rate of 24%, (2) identical proportions between the control group and each intervention group, and (3) a dichotomous dependent variable (i.e., Beryl bike adoption or not). The probability of a Type I error was set at 0.05 (i.e., finding a difference when a difference does not exist) and the probability of a Type II error was set at 0.2 (i.e., not detecting a difference when one actually exists). Given the pen portraits only intervention was less effective at encouraging uptake of Beryl bikes, an even larger initial sample would be required to produce a statistically significant difference between the control group and intervention group B.
