# Supplementary material for: Behavior change interventions to promote adoption of e-bike shared mobility in a rural area: evidence from a mixed-method field trial
Source: Front Psychol. 2025 May 20;16:1569176. doi: 10.3389/fpsyg.2025.1569176 (PMC12131865; doi:10.3389/fpsyg.2025.1569176)
Supplement: Supplementary file 1 [file Data_Sheet_1.pdf]

M. Wilson & L. Whitmarsh. Supplementary material - adoption of e-bike shared mobility

There are eleven appendices in the supplementary materials:

1. Intervention 2: Pen portraits visioning tool 2
   - An older couple living in a rural area 3
   - A young adult living in an urban area 5
   - Middle-income parents 7
   - A single parent on lower income 9
   - A small business owner 11
   - A young adult who uses a wheelchair 13
2. Survey 1 protocol (pre-intervention) 15
3. Travel diary protocol 43
4. Survey 2 protocol (post-intervention) 48
5. Mode shift emission reduction calculations 65
6. Residents' perceptions of Beryl bikes, pre- and post-intervention 68
7. Council staff perceptions of Beryl bikes, post-intervention 70
8. Attrition rate over the intervention period 71
9. Access to Beryl bikes 72
10. Measuring change in physical activity 74
11. Study 1 - focus group protocol 75

1. Intervention 2: Pen portraits visioning tool

The first appendix is the pen portraits visioning tool that was adapted from Prosser et al. (2022) for the Cornwall context. This intervention was presented to intervention groups B and D in this study.

Instructions

For the next stage, you will be presented with a scenario which describes how someone has changed their travel behaviour in some way. There are six short stories of people who have successfully reduced their car use (takes approx. 3 minutes to read).

Please consider your personal situation…where you live in Cornwall, your job, your family commitments, your transport needs. Then choose the character which you think might be the closest to your situation.

If you decide their story isn't relevant for your situation, you can choose a different story to read. These stories do not capture the experiences of everyone living in Cornwall, but there may be some aspects which you find relatable.

Please select one of the characters to read their story, then click the 'next' button:

- ○ an older couple living in a rural area (1)
- ○ a young adult living in an urban area (2)
- ○ middle-income parents (3)
- ○ a single parent on lower income (4)
- ○ a small business owner (5)
- ○ a young adult who uses a wheelchair (6)

An older couple living in a rural area

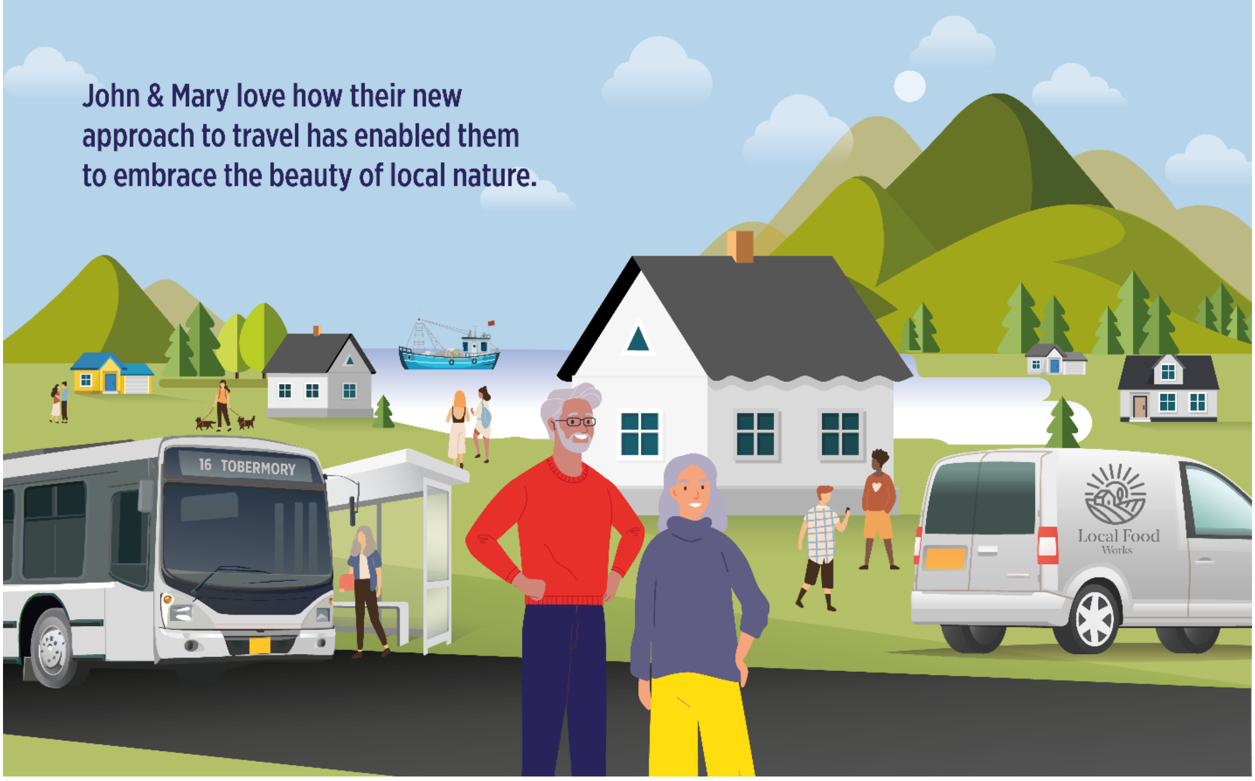

Mary and John, an older couple living in a rural area

Mary and her husband John have lived in Cornwall all their lives and a lot has changed over that time. Now in their 60s, one thing they've noticed is how reliant they are on their cars to get around, compared to when they were younger. They love living in Cornwall and they think rural life should be protected as it's such an important part of Cornwall's tradition and identity. There's a strong sense of community in their village and a slower pace of life, which is really nice compared to the hustle and bustle of the towns.

Previous travel behaviour

Living in a rural area has made it very difficult to manage without a car. For John, a retired engineer, cars are a hobby, as well as a bit of a status symbol. He takes pride in his car and it reminds him that his hard work has paid off. For Mary, driving has always felt necessary, but a bit stressful and exhausting. The roads are really narrow and windy in their area, so she has to be very focused to make sure she can see horse riders or any oncoming cars, especially on the corners. Even John finds it a bit tiring to do long drives on the country lanes, but they'd never really considered any other way of getting around.

New travel behaviour with less car use

During the pandemic, they found out about different food delivery options and decided to

get a delivery once a week, which reduced their need to drive to the supermarket. Their neighbours, who they are friendly with, offered to give John and Mary lifts whenever they were driving into town. John and Mary have also started organising trips with friends who live in the village, travelling together on the bus, which allows them to get into town for the day without the stress of dealing with traffic or finding somewhere to park. They don't go into town very often, but it makes for a nice day out. Mary, who is over 66, gets to travel free with her bus pass and she likes poking fun at John, who still has to pay.

Benefits they've experienced as a result of changing their travel behaviour
Mary and John remembered how much they enjoyed walking and exploring Cornwall when they were younger, so they decided to make walking a bigger part of their lives again. Some friends recommended the 'iWalkCornwall' app and the South West Coast Path Association website, which gives them lots of ideas for different walks to do in their area. They've rediscovered a sense of pride, getting back into the beautiful nature and coastline on their doorstep, and they are feeling much fitter and healthier. Another real change is that they are seeing more of their neighbours and friends now. It takes a bit of planning to use the buses and trains, but not driving means they've got more time when they travel to chat, read a book, or just look out the window and take in the lovely scenery.

Click the left arrow to return to the list of characters.
Click the right arrow to move onto the next task.

A young adult living in an urban area

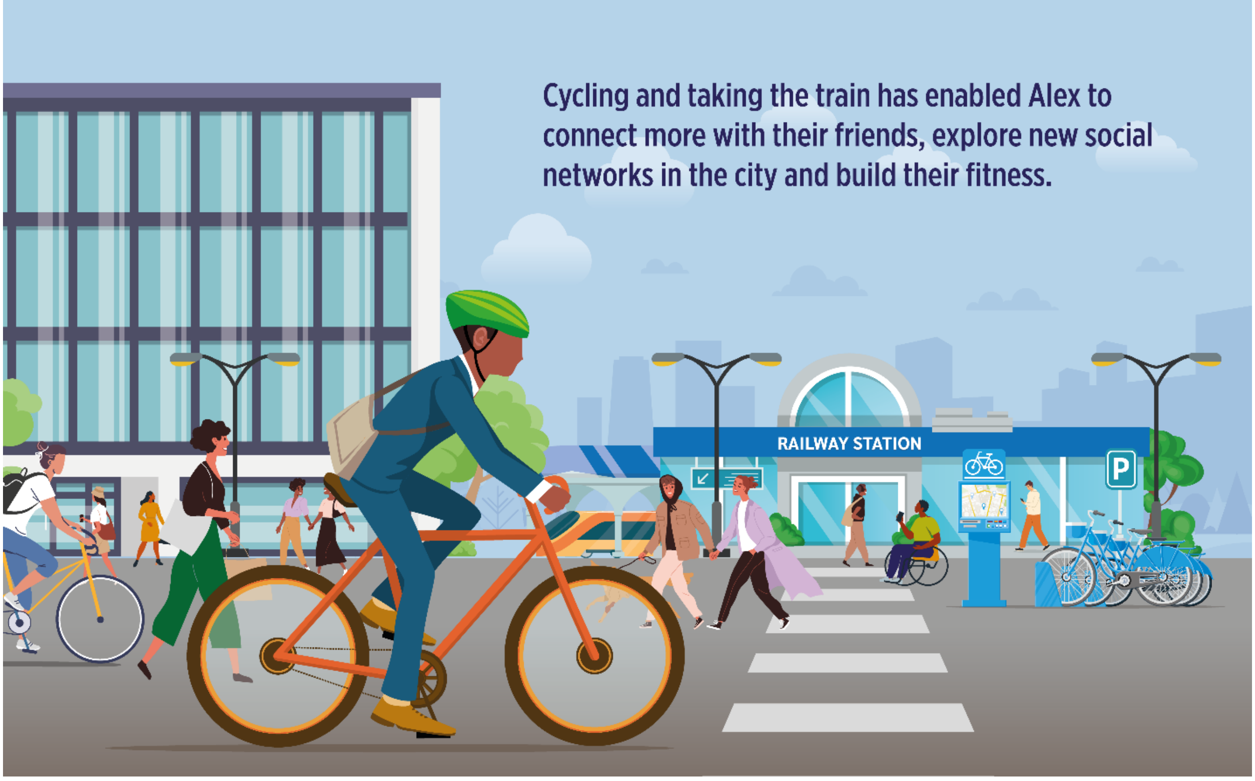

Alex, a young adult living in an urban area

Alex left the family home to go to university and has just started a graduate role in the large town they studied in. They lived in a village in rural Cornwall with their parents until starting their degree. Alex was keen to move to a more urban and diverse area, and they were excited to explore all that the town has to offer.

Previous travel behaviour

They'd always used their parents' old car when living at home and so they brought the car to university with them. They thought this would be the most economical option as trains in Cornwall are quite expensive. Having the car also meant they could return home more often. Since Alex moved to the town, the car has become a burden. It took a bit of time to get the parking permit sorted, but even after that, parking is becoming more and more difficult to find. Sometimes they have to park quite far away and then walk the rest of the way home. The parking permit was expensive and so is petrol, and this puts a strain on their disposable income.

New travel behaviour with less car use

Alex started thinking about what life might be like without their own car. They've been feeling bad about having a car, given the environmental impact, but it was difficult to figure

out other options that they could afford. The car was pretty old and they'd started having problems with it which were expensive to fix. Taking the car to the garage was really time consuming as well, so they started trying other ways to travel. The cost of public transport seemed a bit steep at first, about £60 a month, but this worked out cheaper than running a car once you added up the insurance, MOT, repairs, road tax, and petrol. They were also able to get a Young Persons Railcard, which means they can more easily afford the train home. They did a bit of research online and realised how many places in Cornwall you can explore without a car, to get into nature, or to go to the beaches. They also heard about a 'Cycle to Work' scheme offered by their employer. They've just bought a brand new commuter bike that they pay for monthly and save a bit of money on tax.

Benefits they've experienced as a result of changing their travel behaviour
Getting rid of the car has been a game changer for Alex. Parking in the town was stressful and expensive, and catching the train home is a lot more enjoyable than having to concentrate on driving. They try to plan ahead to buy the cheapest tickets and they've managed to find a return ticket for only £25. Cycling has been really great for Alex's fitness and wellbeing, and joining a local cycle group on Facebook means they've made some new friends and gained a new hobby too! Alex isn't worried about bike theft as they bring the bike inside at home and can use the bike lock-up unit at work. It took some time to build up their confidence riding the bike, but they've really enjoyed not having the responsibility of a car. They realised that, without any real burden on their finances, they've found a way to reduce their carbon footprint and see more of the countryside.

Click the left arrow to return to the list of characters.
Click the right arrow to move onto the next task.

Middle-income parents

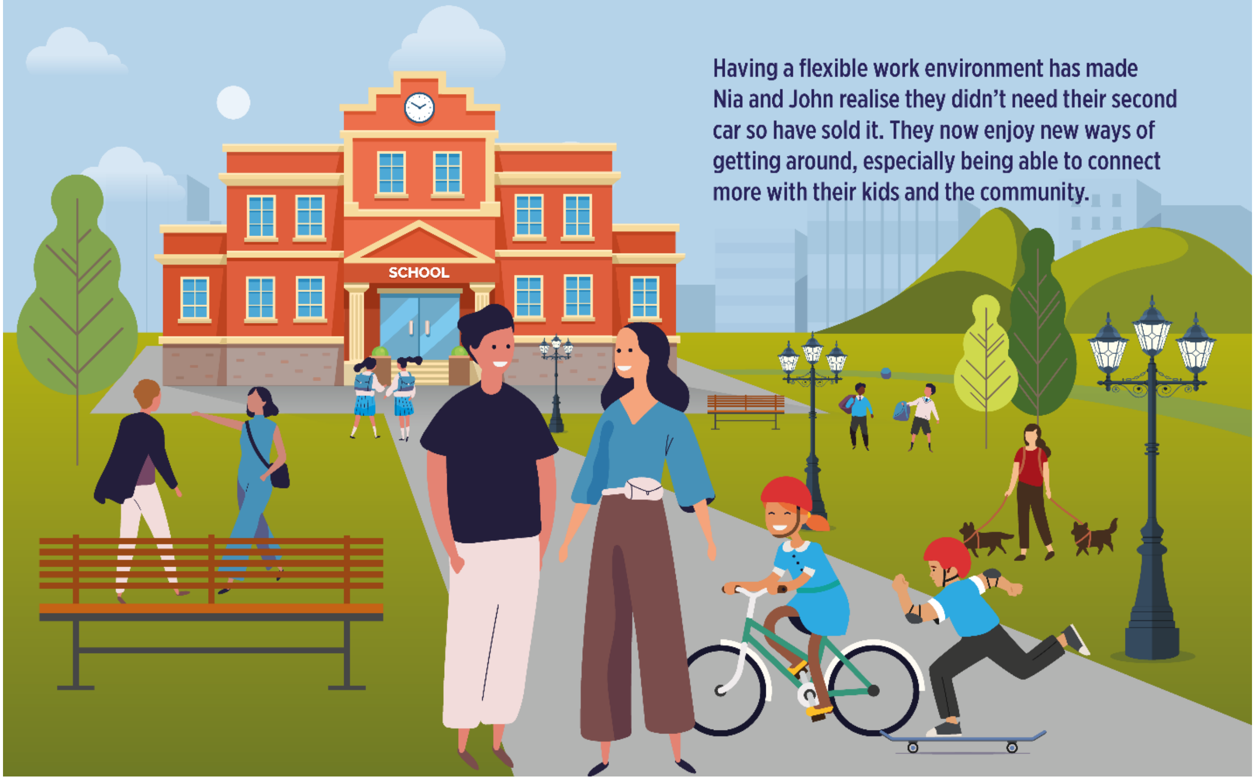

Nia and John, middle-income parents

Nia and John are in their mid-30s and have two children who are at primary school. Nia works as a software developer and John works at a marketing firm. They live in a large town, in a desirable area, with a park and lots of local shops and cafés. Their house is within walking distance of the school their children attend. Their children like going to the park on the way home from school.

Previous travel behaviour
Nia and John didn't have much time to spare in the morning before work, so they would take it in turns to drive their children to school. The traffic was usually busy, and the exhaust fumes and noise near the school put them off walking. They each had their own car to drive to work. They also used the car for the big weekly food shop every Saturday, at the large supermarket on the outskirts of town.

New travel behaviour with less car use
Nia and John's workplaces introduced flexible working policies during the pandemic and so now they often work from home, which means less driving. They noticed that quite a few families had started walking their kids to school. Their own children had been asking them to walk more because they learned about climate change and the environment in one of their

classes. Although it took a while to establish a new routine, they've started to really enjoy walking the kids to school. It gives them some quality family time in the morning and some fresh air before work. Now they are exploring their neighbourhood on foot and they've realised they can buy most of the groceries they need locally. They only go to the big supermarket when there's something they can’t find very easily in the local shops, and so now they have more free time on the weekends. They decided they probably only need one car, so they agreed to sell one and see how it goes. They use the other car a lot less than before, mainly for day trips or visiting family and friends.

Benefits they’ve experienced as a result of changing their travel behaviour

They realised the air pollution around the school was so bad because everyone would drive, but after a public consultation organised by the school and the local community, a 'school streets' initiative was set up which reduces traffic near the school. They’ve found it inspiring to see how the kids from the school have encouraged and motivated the adults to try something new, which is better for the environment and for their health! Nia used the money from selling her car to buy new bikes for the family and put the rest of the money in their savings. It did take a while to build their confidence on the bikes, but over time they've realised they are actually quite good at it and they've started to feel much fitter. The new 20 mph speed limits made things feel safer and more people seem to be out cycling. The high street has now been pedestrianised, so it’s become a pleasure to get around by bike or walking. They often bump into people they know, and this has helped Nia and John to feel more connected to their community.

Click the left arrow to return to the list of characters.
Click the right arrow to move onto the next task.

A single parent on lower income

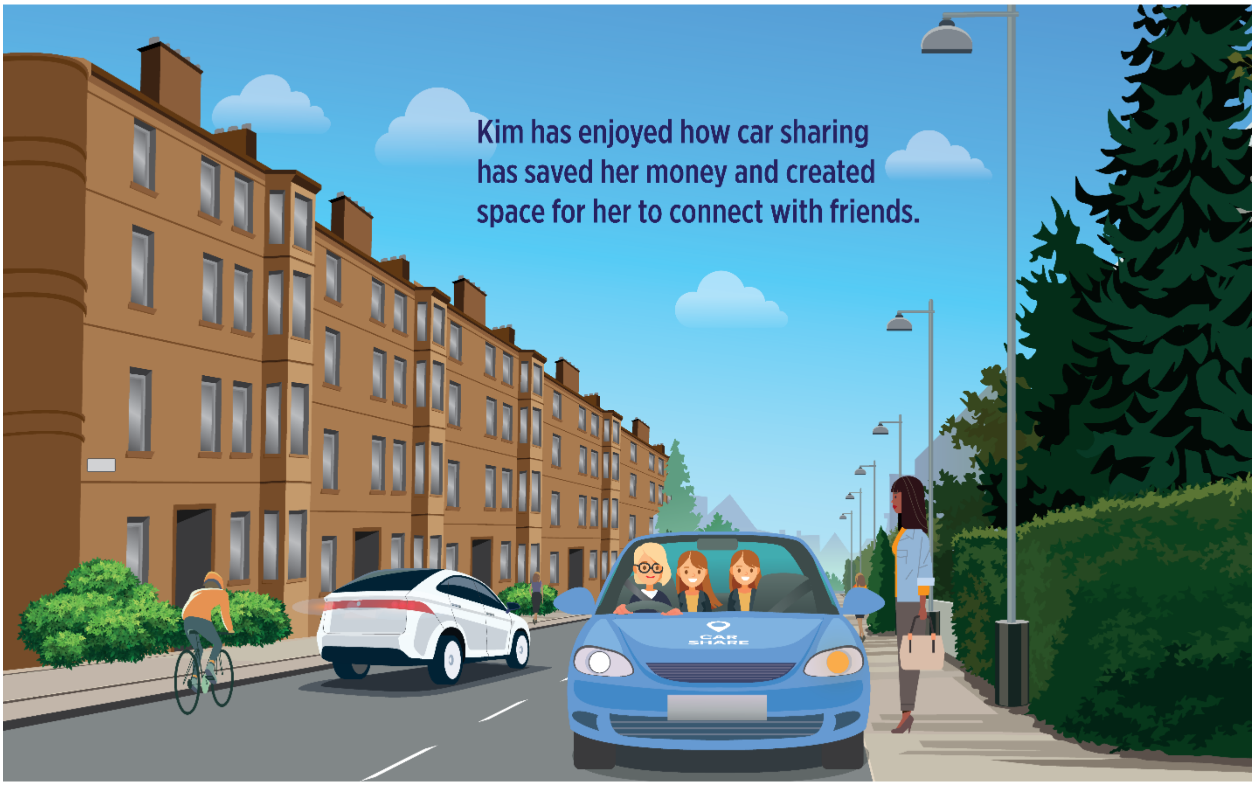

Kim, a single parent on a low income

Kim and her children live in a small town in Cornwall. Her children are twins and they are fourteen years old. After the twins got settled in secondary school and became more independent, Kim decided to take on some extra work to support her family's increasing living costs. She now works two jobs, one at a factory on the industrial estate, and one as a receptionist in the evenings at a hotel in the neighbouring town, which is about 7 miles away. She doesn't get much free time nowadays, but she enjoys spending time with the kids, and meeting up with her friends on her days off.

Previous travel behaviour

Kim has a car, but she found it difficult to afford the running costs. The local bus service finishes early and so public transport wasn't really a viable option to get home after her hotel shift. She doesn't enjoy driving, but having the car means Kim could get to work, pick up the kids after school, and go into town to meet her friends. She'd heard about people being more environmentally friendly, but she was sceptical that other transport options were affordable and convenient enough, and so she stuck with what she knew worked best for her and her family.

New travel behaviour with less car use

Kim finds that driving everywhere is expensive and tiring, and she wanted to find another way to commute to the hotel. After chatting with her friend, Jessie, she found out that Jessie had started a new job in the hospice, which is quite close to the hotel. Jessie was struggling a bit with living costs and so they decided to take turns in giving each other lifts to work, when their shifts align. Kim also saw an advert about reduced prices for bus tickets, which now cost only £2 for all buses in Cornwall. She's started using the bus when she needs to go into town and this also saves on parking fees.

Benefits she's experienced as a result of changing her travel behaviour

Lift sharing means Kim can spend more time with Jessie. Chatting with her friend is a much nicer way to start and end her shift, and she saves a bit of money each week on petrol. She mentioned lift sharing to her manager, who thought it was a good idea and so the factory is trialling a scheme which encourages everyone to share lifts to and from work. So far, it seems to be working well.

Click the left arrow to return to the list of characters.
Click the right arrow to move onto the next task.

A small business owner

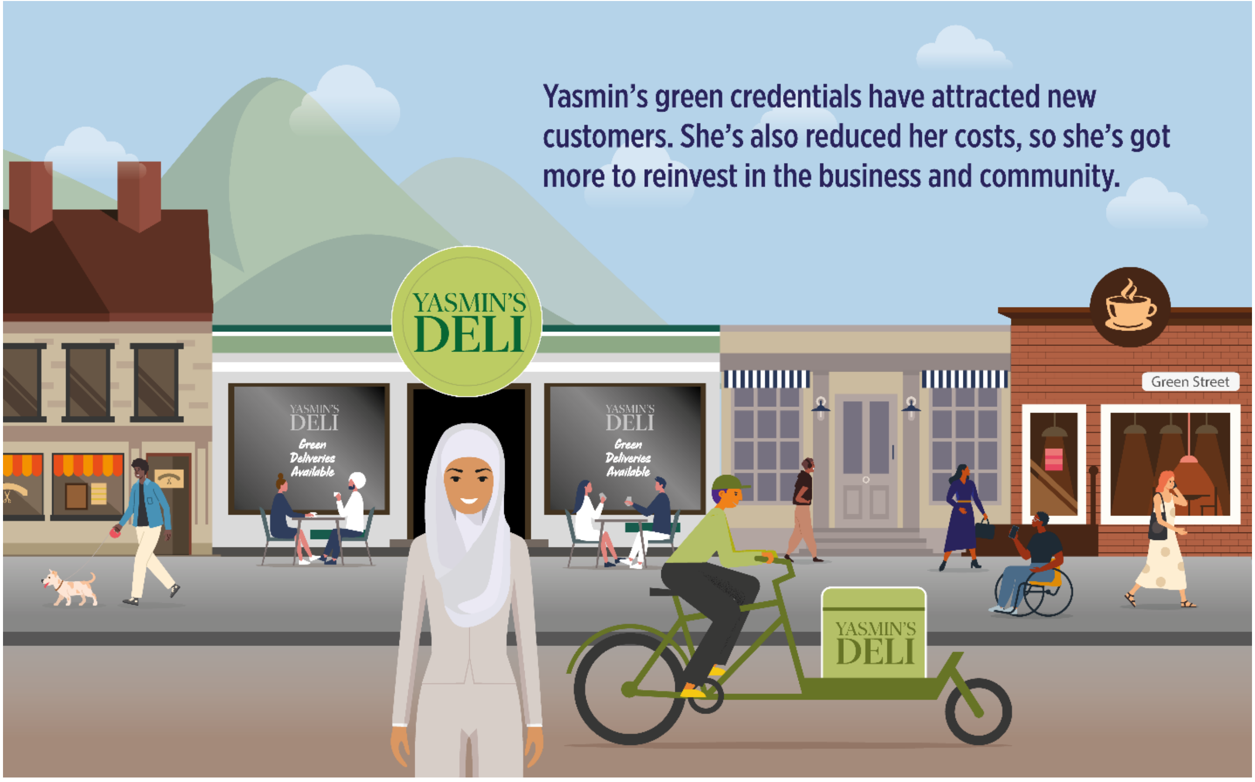

Yasmin, a small business owner

Yasmin owns a popular restaurant and deli supermarket in the centre of a rural town in Cornwall. She is recognised across the community because she organises a number of fun events throughout the year, like cooking classes and pop up street food stalls.

Previous travel behaviour

Running the business keeps Yasmin very busy and she found it difficult to spare the time for walking or getting public transport around town. She often needs a car to get to the restaurant for the early morning deliveries. She began offering a home delivery service to her customers, so her business started to depend on cars and motorbikes much more than it used to. She was concerned about the environmental impact, but she felt she didn't have the time or any alternative options to compromise on the swift delivery and high-quality customer service she was known for.

New travel behaviour with less car use

Yasmin realised that some of her competitors in the area were starting to use more environmentally friendly methods for home delivery. With growing public concern for the environment, and the rising cost of using cars for home delivery, she decided to change her business model. For local deliveries, she now employs delivery riders who use e-bikes, which

reduces her overheads as well as the carbon footprint of her business. When the traffic is bad, the delivery riders are actually quicker than delivering by car. She's installed a charging point at the back of the shop, so they can recharge the e-bikes easily. Initially, she wasn’t sure how this change would go, but overall it’s really helped the efficiency of the deliveries, especially during spring, summer and autumn when the weather is ok. After chatting to one of the delivery riders, she felt inspired to look into e-bikes as an option for herself to get around. She was pleasantly surprised to hear that a bike share scheme, Beryl bikes, has just started in her town. She is using Beryl bikes for a couple of months while she decides whether to buy her own e-bike.

Benefits she’s experienced as a result of changing her travel behaviour
Yasmin is relieved that she no longer has to choose between offering good customer service and reducing her environmental impact. She’s started promoting her business based on its green credentials and has since attracted new customers who had heard about her deliveries by e-bike. Reducing costs, and the increase in business that she’s seen, means she can invest more money into growing her business.

Click the left arrow to return to the list of characters.
Click the right arrow to move onto the next task.

A young adult who uses a wheelchair

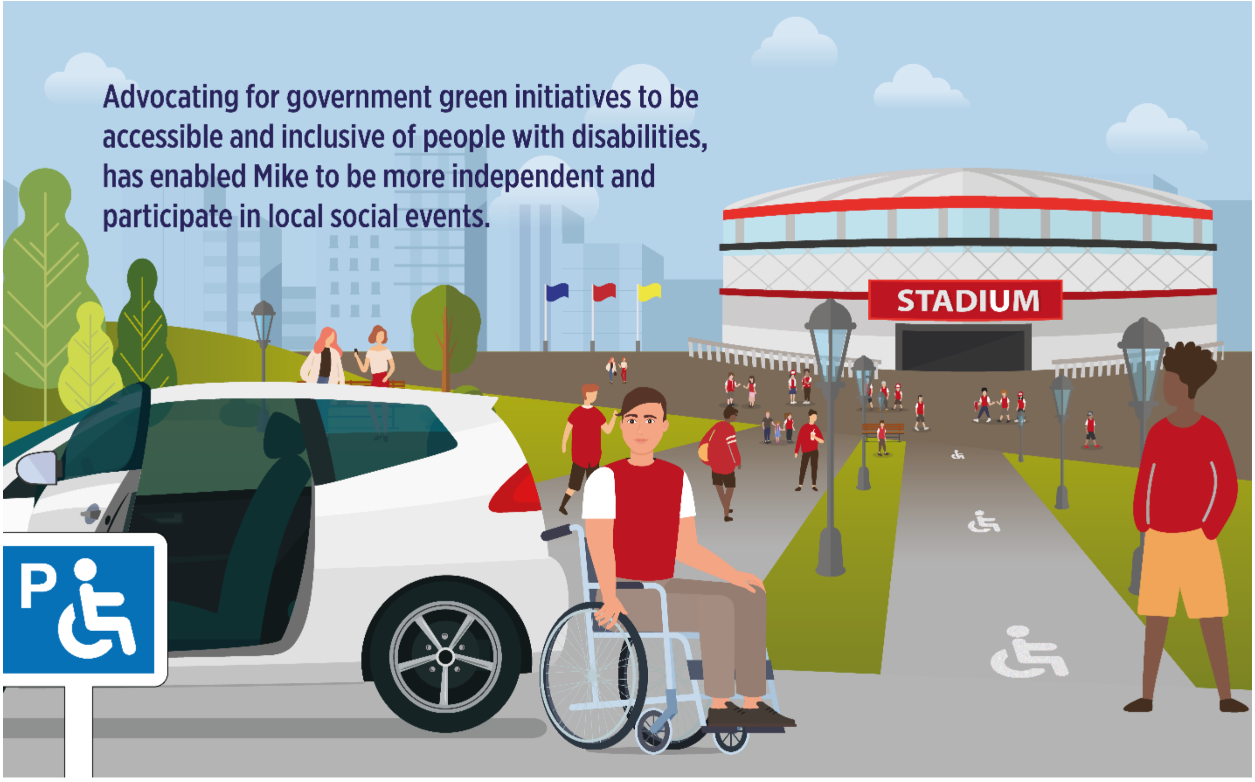

Mike, a young adult who uses a wheelchair

Mike lives on the outskirts of town with his girlfriend Niamh. He works as an IT specialist for a large sales company based in the town centre. Mike lives with a physical impairment which seriously affects his mobility and so he is assisted by a wheelchair all of the time. This can make it quite challenging and tiring, as not all public spaces are designed to be inclusive of people like Mike. In his spare time, he loves to watch football matches at his club and pop into his local pub for a drink afterwards.

Previous travel behaviour

Mike is able to live independently because he has a car. Driving into work was really his only option – even that took careful planning and would sometimes leave him pretty exhausted. When he joined the company, he worked with them to build their awareness about how important the option of working from home is for people living with disabilities. They learned a lot from him and agreed for him to work from home most of the time, but come into the office once a week because he really enjoys and values seeing the team, and some clients, in person. Thankfully, the building is disability inclusive, with ramps and automatic doors, but there were still a few improvements which he suggested.

New travel behaviour with less car use

Since the pandemic, everyone has been working from home some of the time, but Mike's team now have scheduled days when they agree to come into the office. It's a good way to make sure people get face-to-face contact and keep up to date on the issues that aren't really discussed in their online meetings. His positive experience with his employer empowered him to write to his local football club about the limited number of disability parking spots at the stadium, and other challenges for wheelchair users such as the lack of dropped kerbs or wide pavements. Although the club did take a while to respond, they agreed to increase the disability parking spots by three and install wheelchair accessible infrastructure.

Benefits he's experienced as a result of changing his travel behaviour

Mike has often found it difficult to engage with the environmental movement, so knowing that he's already reducing his carbon footprint by not driving into work every day, but still maintaining his independence, feels good. Mike heard on local radio about some actions the Council were taking to create pedestrianised areas and encourage Cornwall residents to use active ways to travel. He is a passionate advocate of improving public spaces to ensure they are accessible and inclusive, and he wanted to make sure that any new travel infrastructure or initiatives to reduce carbon emissions didn't overlook being disability inclusive. Having already worked with his company and his football club, he feels he can offer some valuable input in this process of improving his local town that he loves.

Click the left arrow to return to the list of characters.
Click the right arrow to move onto the next task.

2. Survey 1 protocol (pre-intervention)

The second appendix is the pre-intervention survey.

Block 1: Intro and consent

Travel behaviour survey 1

Information about this study

What is this study about?

We are researchers at the University of Bath working with Cornwall Council to understand what is important to people living in Cornwall and what influences their lifestyles and travel habits.

What does the study involve?

You will receive a behaviour change intervention that relates to your travel options. We will ask you to complete three data collection activities:

1. an online survey (1) that will take 10 - 15 minutes
2. an online travel diary once a week, for four weeks. The diary will take approximately 3 minutes to complete each week
3. an online survey (2) that will take about 10 minutes

You will be asked questions about your current travel behaviours and your views on different ways to travel. We estimate your total time commitment for all activities to be one hour.

Who can take part?

Anyone (aged 16+) who currently lives or works in one of these towns/city: Truro, Falmouth, Penryn, Newquay, Penzance and St Austell. You must also have an interest in active ways to travel (e.g. walking, cycling, wheeling). You will need to own a smartphone.

What are the benefits and risks of taking part?

The information you provide will be very useful for the research team and Cornwall Council to understand what people living in Cornwall think about different travel options. You will receive **a £25 gift voucher when you complete survey 2**. There are no risks associated with participating.

This research has been reviewed and approved by the University's Department of Psychology Research Ethics Committee (reference: 23 - 079).

Do I have to take part?
Taking part in this study is entirely voluntary. You are free to withdraw at any time. You can withdraw by simply closing your browser during any of the data collection activities described above. When you complete the final data collection activity (survey 2), we will anonymise your responses. Your data would then be anonymous and cannot be traced back to you, and so we would be unable to identify and remove your data.

What happens to all the information?
All the information you provide is confidential and will be stored on a secure drive at the University of Bath (encrypted and password-protected). Anonymised data will be archived indefinitely in the UK Data Archive and may be used by the Council or other researchers in future studies. The University of Bath privacy notice can be found here. If you choose to withdraw from the study, we will remove all of your data.

We will ask for your email address. Your contact details will be stored separately from your survey data, so your responses cannot be identified. We will use your email address for two purposes: 1) to send you links for the online travel diaries, and 2) to send you the gift voucher. Your email address will be permanently deleted within 14 days of the study completion.

What do I do if I have any questions?
Please contact the research team at the University of Bath for further information: Mark Wilson (mw2640@bath.ac.uk) or Lorraine Whitmarsh (lw2253@bath.ac.uk).

Or if you have any concerns about this study, please contact the Department of Psychology Research Ethics Committee: (psychology-ethics@bath.ac.uk; +44 (0)1225 384714).

Department of Psychology
University of Bath
Claverton Down
Bath, BA2 7AY

How can I take part?
Please click the arrow below

Consent Form
Please indicate that you have read and understood the following statements:

1. I understand the nature and purpose of the procedures involved in this study. These have been communicated to me on the information sheet on the previous page.

2. I understand that my participation in this study is entirely voluntary. I can withdraw from the study by closing the browser during any of the data collection activities. Once I complete the final data collection activity (survey 2), my data is anonymised and can no longer be withdrawn from the study.

3. I understand and acknowledge that this study is designed to promote scientific knowledge and may be used by Cornwall Council to inform policy and delivery.

4. I understand that I will be asked to provide my email address. My email address will be permanently deleted within 14 days of the study completion. My data will be anonymised when I complete the final data collection activity, so I cannot be identified in any research outputs.

5. I understand that the University of Bath may use the data collected for this project in a future research project but that the conditions on this form under which I have provided the data will still apply. Anonymised data stored on the UK Data Archive may be used by the Council or other researchers in future studies.

6. I understand that the personal data will be processed in accordance with current UK data protection legislation. The University of Bath privacy notice can be found here.

7. I understand that I am free to discuss any concerns I may have with the research team: Mark Wilson (mw2640@bath.ac.uk) or Lorraine Whitmarsh (lw2253@bath.ac.uk).
If they are unable to resolve your concern or you wish to make a complaint, please contact the Department of Psychology Research Ethics Committee: (psychology-ethics@bath.ac.uk; +44 (0)1225 384714). The PREC reference number for this study is: 23 079.

I have read the above statements and consent to take part:

- ◯ I **CONSENT** to take part in the study (1)
- ◯ I **DO NOT CONSENT** to take part in the study (2)

Q247 I confirm I am aged 16 years or older, I own a smartphone, and I currently **live or work** in Truro, Falmouth, Penryn, Newquay, Penzance or St Austell:

- ◯ Yes (1)
- ◯ No (2)

Please click the right arrow to continue.

Block 2: Baseline travel behaviours

About how you travel

These questions are about how you commute to work and other travel you do (e.g. for shopping, visiting friends etc.).

Q1 Do you own or have regular access to a vehicle (e.g. a car, van or motorbike)?
(Please select all options that apply)

- ▢ Yes - a petrol or diesel vehicle (1)
- ▢ Yes - a hybrid vehicle (2)
- ▢ Yes - an electric vehicle (3)
- ▢ Yes - a car share scheme (e.g. Co-cars) (4)
- ▢ No (5)

Q111 In a typical week, how many journeys per week do you make to / from your place of work or education (i.e. **commuting**) using the following modes of transport?

	Number of commute journeys per week *(travelling there and back would count as two journeys)* (1)
N/A - I don't work or I work entirely from home *(just write 1 in the box)* (Q111_11)	

Walking,<br>or Wheeling (i.e. use a wheelchair) (Q111_1)	
Bicycle (including electric bike) (Q111_2)	
Scooter (including electric scooter)<br>(Q111_3)	
Motorbike (Q111_4)	
Car / van (travelling alone) (Q111_5)	
Car / van (sharing lifts with others) (Q111_6)	
Car club (e.g. Co Cars) (Q111_7)	
Bus (Q111_8)	
Train (Q111_9)	
Other (please specify) (Q111_10)	

Q213 If you commute, approximately how far (in miles) is your home to your place of work / study?

____

Q112 In a typical week, how many journeys per week do you make to / from other destinations (e.g. to the shops, visiting friends) using the following modes of transport?

	Number of non-work journeys per week *(travelling there and back would count as two journeys)* (1)
N/A - I tend to stay at home *(just write 1 in the box)* (Q112_11)	
Walking,<br>or Wheeling (i.e. use a wheelchair) (Q112_1)	
Bicycle (including electric bike) (Q112_2)	
Scooter (including electric scooter) (Q112_3)	
Motorbike (Q112_4)	
Car / van (travelling alone) (Q112_5)	

Car / van (sharing lifts with others) (Q112_6)	
Car club (e.g. Co Cars) (Q112_7)	
Bus (Q112_8)	
Train (Q112_9)	
Other (please specify) (Q112_10)	

Q304 Which statement best describes your current car driving?

- ○ I drive, and am not interested in reducing my car use (1)
- ○ I drive, but would like to reduce my car use (2)
- ○ I do not drive, but would like to start doing so (3)
- ○ I do not drive, and have no interest in doing so (4)
- ○ Don't know / None of the above (5)

Display Logic: present Q331

If Q1. Do you own or have regular access to a vehicle (e.g. a car, van or motorbike)? = Yes - a petrol or diesel vehicle OR Yes - a hybrid vehicle OR Yes - an electric vehicle OR Yes - a car share scheme (e.g. Co-cars)

Q331 In a typical week, how often do you use a car for short journeys (i.e. less than 3 miles)?

	0 (8)	1 (1)	2 (2)	3 (3)	4 (4)	5 (5)	6 (6)	7 (7)
Number of days	○	○	○	○	○	○	○	○

per week: (6)

Block 3: Active travel

About Active Travel

These questions are about active ways to travel (e.g. walking, cycling, wheeling) and physical activity.

Q245 Please tell us about your current bike ownership:

- ▢ I own a conventional pedal bike (1)
- ▢ I own an e-bike (i.e. an electric bike) (2)
- ▢ I own a bike but it is in disrepair (3)
- ▢ I used to own a bike but I got rid of it (4)
- ▢ I have never owned a bike (5)
- ▢ Not applicable / I can't ride a bike (6)
- ▢ Other (please specify) (7) ____

(Note – routing question for intervention group allocation in Block 7 – Travel intervention)

Q186 To what extent have the following prevented you from walking or cycling as a main mode of travel?

	Not at all (1)	A little (2)	Somewhat (3)	A lot (4)	Not applicable (99)
Lack of cycle lanes or walking paths (Q186_1)	○	○	○	○	○
Feeling unsafe cycling on	○	○	○	○	○

roads (Q186_2)					
Lack of cycling confidence or competence (Q186_3)	○	○	○	○	○
The distance is too far (Q186_4)	○	○	○	○	○
Not feasible due to long-standing illness, injury or disability (Q186_5)	○	○	○	○	○

Q381 During the last week, how many hours did you spend on each of the following activities?
(Please move the slider into the correct position)

Number of hours

0 1 2 3 4 5 6 7 8 9 10

Physical exercise such as swimming, jogging, aerobics, football, tennis, gym workout etc. ()

Cycling, including cycling to work and during leisure time ()

Walking, or **Wheeling** (i.e. use a wheelchair), including walking / wheeling to work, shopping, for pleasure etc. ()

Block 4: e-bikes

About e-bikes and bike share

You're doing great! These questions are about your views on e-bikes and bike share schemes (e.g. Beryl bikes).

Q285 How likely is your household to buy an e-bike (or another e-bike) in the next 12 months?

- ◯ Very unlikely (1)
- ◯ Somewhat unlikely (2)
- ◯ Neither likely nor unlikely (3)
- ◯ Somewhat likely (4)
- ◯ Very likely (5)
- ◯ Don't know (77)

Display logic: present Q292

If Q245, Please tell us about your current bike ownership: = I own an e-bike IS NOT SELECTED

Q292 What are your opinions on owning an e-bike?

	Strongly disagree (1)	Somewhat disagree (2)	Neither agree nor disagree (3)	Somewhat agree (4)	Strongly agree (5)
My household could easily afford to buy an e-bike (Q292_1)	◯	◯	◯	◯	◯
Storing an e-bike at my home would be difficult (Q292_2)	◯	◯	◯	◯	◯

If I owned an e-bike, I would worry about it getting stolen (at home or when out) (Q292_3)	○	○	○	○	○

Display logic: present Q293

If Q245, Please tell us about your current bike ownership: = I own an e-bike IS SELECTED

Q293 What are your opinions on owning an e-bike?

	Strongly disagree (1)	Somewhat disagree (2)	Neither agree nor disagree (3)	Somewhat agree (4)	Strongly agree (5)
My household could easily afford to buy the e-bike(s) we own (Q293_1)	○	○	○	○	○
Storing an e-bike at my home is difficult (Q293_2)	○	○	○	○	○
I worry about my/our e-bike getting stolen (at home or when out) (Q293_3)	○	○	○	○	○

Q289 Please give your opinions about regularly using an e-bike yourself:

	Strongly disagree (1)	Somewhat disagree (2)	Neither agree nor disagree (3)	Somewhat agree (4)	Strongly agree (5)	Don't know / Not applicable (77)
I would find it easy to ride an e-bike if I wanted to (Q289_1)	○	○	○	○	○	○
People who are important to me would support me using an e-bike (Q289_2)	○	○	○	○	○	○
I see myself as the kind of person who might regularly ride an e-bike (Q289_3)	○	○	○	○	○	○

Q287 Apart from yourself, do you know anyone personally (e.g. friends, family, neighbours, work colleagues) who regularly uses an e-bike?

○ Yes (1)

○ No (2)

○ Don't know (77)

Q223 Bike share schemes (e.g. Beryl bikes) are relatively new in Cornwall.
Have you already used a bike share scheme?

☐ Yes - I've used Beryl bikes in Cornwall (1)

☐ Yes - I've used a bike share scheme in another city / location (2)

☐ No (3)

Q295 How did you first hear about Beryl bikes in Cornwall?

○ Taking part in this study (1)

○ Council website or newsletters (2)

○ Coverage on TV / radio / newspapers / news websites (3)

○ A friend, family member, or colleague told me (4)

○ I saw a Beryl bike docking station / someone riding a Beryl bike (5)

○ Advert on social media / the internet (6)

○ A local notice or flyer promoting Beryl bikes (7)

○ Other (please specify) (8) ____

○ I can't remember (9)

Q267 To what extent do you agree with the following statements about Beryl bikes?

	Strongly disagree (1)	Somewhat disagree (2)	Neither agree nor disagree (3)	Somewhat agree (4)	Strongly agree (5)
It's becoming more common for people in my town to use	○	○	○	○	○

Beryl bikes (Q267_1)					
I'm aware that people where I live and work use Beryl bikes (Q267_2)	○	○	○	○	○

Q302 To what extent do you agree with the following statements about how using Beryl bikes may help you personally? (Part A)

	Strongly disagree (1)	Somewhat disagree (2)	Neither agree nor disagree (3)	Somewhat agree (4)	Strongly agree (5)	Not applicable (99)
It will make my trips quicker (Q302_1)	○	○	○	○	○	○
It will make my trips easier (Q302_2)	○	○	○	○	○	○
I will be able to cycle longer distances (Q302_3)	○	○	○	○	○	○
I can avoid fatigue or getting sweaty before	○	○	○	○	○	○

work or socialising (Q302_4)						
It will help me reduce my carbon footprint (Q302_5)	○	○	○	○	○	○

Q301 To what extent do you agree with the following statements about how using Beryl bikes may help you personally? (Part B)

	Strongly disagree (1)	Somewhat disagree (2)	Neither agree nor disagree (3)	Somewhat agree (4)	Strongly agree (5)	Not applicable (99)
It will provide me with exercise (Q301_6)	○	○	○	○	○	○
It will provide me with mental health benefits (Q301_7)	○	○	○	○	○	○
It will enable me to cycle with friends / family as a group (Q301_8)	○	○	○	○	○	○
It will take away concerns around bike maintenance	○	○	○	○	○	○

and storage (Q301_9)						
It will take away the worry of bike theft (Q301_10)	○	○	○	○	○	○

Q300 To what extent do you agree with the following statements about how using Beryl bikes may help you personally? (Part C)

	Strongly disagree (1)	Somewhat disagree (2)	Neither agree nor disagree (3)	Somewhat agree (4)	Strongly agree (5)	Not applicable (99)
It will save me money (Q300_11)	○	○	○	○	○	○
It will connect me to places not served by public transport (Q300_12)	○	○	○	○	○	○
I will be able to use my car less (Q300_13)	○	○	○	○	○	○
I will avoid traffic congestion / parking difficulties (Q300_14)	○	○	○	○	○	○

I can try an e-bike before I decide whether to buy one (Q300_15)	○	○	○	○	○	○

Q252 In which town(s)/city are you most likely to use Beryl bikes?
(Please select all that apply)

- ▢ Falmouth (1)
- ▢ Penryn (2)
- ▢ Truro (3)
- ▢ St Austell (4)
- ▢ Newquay (5)
- ▢ Penzance (6)
- ▢ Not applicable (77)

Q286 Is there a Beryl bike parking bay within walking distance of your home?

- ○ Yes (1)
- ○ No (2)
- ○ Don't know (77)

Q290 Is there a Beryl bike parking bay within walking distance of your place of work or education?

- ○ Yes (1)
- ○ No (2)
- ○ Don't know / Not applicable (77)

Q316 If you use public transport, is there a Beryl bike parking bay within walking distance of key public transport hubs (e.g. train station, bus station)?

- Yes (1)
- No (2)
- Don't know / Not applicable (77)

Block 5: Concern about climate change

Climate change

Almost there! The next few questions are about your views on climate change and the environment.

Q136 How worried are you about the following issues?

	Not at all worried (1)	Not very worried (2)	Somewhat worried (3)	Very worried (4)	Extremely worried (5)
Climate change (Q136_1)	○	○	○	○	○
Air pollution (in my local area) (Q136_2)	○	○	○	○	○

Q139 Which of these statements best describes your views?
Addressing climate change requires...

- little or no urgency (1)
- a low level of urgency (2)
- a moderate level of urgency (3)
- a high level of urgency (4)
- an extremely high level of urgency (5)

Q150 To what extent would you support or oppose the following policy measures to help tackle climate change?

	Strongly oppose (1)	Tend to oppose (2)	Neither oppose nor support (3)	Tend to support (4)	Strongly support (5)
Low Traffic Neighbourhoods (a small residential area closed off to traffic, for use by pedestrians and cyclists) (Q150_1)	○	○	○	○	○
Low Emission Zones (a zone within a city that polluting vehicles must pay to enter) (Q150_2)	○	○	○	○	○
20 mph speed zones (to protect non-motorised road users and encourage walking and cycling) (Q150_3)	○	○	○	○	○
Restricted car parking in workplaces and town centres (Q150_4)	○	○	○	○	○

Q137 What in your view are the most important actions Cornwall Council should take to reduce carbon emissions **related to travel**?

____

____

Block 6: Demographics

About you

Finally, please tell us a bit more about yourself.

Q77 What kind of property do you live in?

- ◯ Detached house (1)
- ◯ Semi-detached house (2)
- ◯ Terraced house (3)
- ◯ Flat or bedsit (4)

Q83 How many bedrooms does your home have?

- ◯ 1 (1)
- ◯ 2 (2)
- ◯ 3 (3)
- ◯ 4 or more (4)

Q273 What best describes the area where you live?

- ◯ Countryside or small village (1)
- ◯ Large village or small town (2)
- ◯ Suburbs of large town or city (3)
- ◯ Centre of large town or city (4)

Q304 What is the first half of your postcode (e.g. TR1, PL14)?

____

Q210 How do you self-identify?

- ◯ Female (1)
- ◯ Male (2)
- ◯ Non-binary (3)

○ None of the above (if you wish, please specify) (4)

____

○ Prefer not to say (88)

Q301 What is your age (in years)?

____

Q138 What is your ethnic group?
Please choose one option that best describes your ethnic group or background

○ White British / White Cornish (1)

○ Mixed / Multiple ethnic groups (2)

○ Asian / Asian British (3)

○ Black / African / Caribbean / Black British (4)

○ Minority Ethnic / Roma / Gypsy / Traveller (5)

○ Other ethnic group (6)

○ Prefer not to say (88)

Q142 Do you have a long-standing illness, injury or disability that limits your normal day-to-day activities?

By 'long-standing' we mean anything that has troubled you over a period of time. 'Normal day-to-day activities' includes things like eating, washing, walking and going shopping.

○ Yes (1)

○ No (2)

○ Prefer not to say (88)

Q302 How many adults (aged 18 or older), including you, live in your home?

____

Q144 How many children (under 18) live in your home?

____

○ Prefer not to say (88)

Q178 What is the highest level of education you have achieved so far?

- No formal qualifications (1)
- GCSE or O-level (2)
- A-level (3)
- Undergraduate degree (e.g. Bachelor's) (4)
- Postgraduate degree (e.g. Master's, PhD) (5)
- Vocational qualification (6)
- Other (7)
- Prefer not to say (88)

Q165 Which option best describes your employment status?

- Employed full time (30+ hrs/wk) (1)
- Employed part time (less than 30 hrs/wk) (2)
- Self-employed (3)
- Unemployed (4)
- Looking after home / family (5)
- Studying (6)
- Retired (7)
- Other (8)
- Prefer not to say (88)

Q145 Please indicate the approximate combined income of your **household** (per year, before tax deductions):

- Less than £6,000 (1)
- £6,000 - £12,999 (2)
- £13,000 - £18,999 (3)
- £19,000 - £25,999 (4)

- ◯ £26,000 - £31,999 (5)
- ◯ £32,000 - £47,999 (6)
- ◯ £48,000 - £63,999 (7)
- ◯ £64,000 - £95,999 (8)
- ◯ More than £96,000 (9)
- ◯ Prefer not to say (88)

Block 7 – Travel intervention. Random allocation to one of four groups: A, B, C or D

Group A - Control group
Q360 Thank you for completing the first survey. The next stage of this study is to complete a weekly travel diary - I'll send you the first travel diary in a week's time.

We asked you about your views on bike share schemes. Beryl bikes are supported by Cornwall Council and are available in Truro, Falmouth, Penryn, Newquay, Penzance and St Austell. You can hire Beryl bikes for single journeys (pay as you ride), or for multiple journeys (you buy 'minute bundles', to use when you need).

More information about Beryl bikes can be found here.

Q323 What is your email address?

(We will only use your email address to: 1. send you the weekly travel diaries, and 2. send you the £25 gift voucher at the end of the study)

Debrief

Group B - Pen portraits only
Thank you for completing the first survey.

We asked you about your views on bike share schemes. Beryl bikes are supported by Cornwall Council and are available in Truro, Falmouth, Penryn, Newquay, Penzance and St Austell. You can hire Beryl bikes for single journeys (pay as you ride), or for multiple journeys (you buy 'minute bundles', to use when you need).

More information about Beryl bikes can be found here.

Q249 What is your email address?
(We will only use your email address to: 1. send you the weekly travel diaries, and 2. send you the £25 gift voucher at the end of the study)

Survey logic: present pen portraits tool

Please reflect on their story and answer the following questions:

Q410 How relevant did you find the scenario to your own life and travel needs?

- ○ Not at all relevant (1)
- ○ Not very relevant (2)
- ○ Somewhat relevant (3)
- ○ Very relevant (4)
- ○ Completely relevant (5)

Q411 To what extent did the scenario make you feel you could reduce your car use?

- ○ Not at all (1)
- ○ A little (2)
- ○ A moderate amount (3)
- ○ A lot (4)
- ○ Completely (5)
- ○ Not applicable / I don't use a car (6)

Q413 Did their story give you any ideas for how you might change how you travel in Cornwall, or reduce your need to travel?

- ○ Not at all (1)
- ○ A little (2)
- ○ A moderate amount (3)

- ○ A lot (4)
- ○ Completely (5)

Q415 Thank you! The next stage of this study is to complete a weekly travel diary - I'll send you the first travel diary in a week's time.

During the next four weeks, think about how you could reduce your car use. Please also consider what benefits or drawbacks you experience from making these changes.

Debrief

Group C - Beryl bikes only
Q322 Thank you for completing the first survey. I'll send you the first travel diary in a week's time.

For the next stage, you will receive free credits to use Beryl bikes. Beryl bikes are e-bikes for public use that you can hire for single journeys (pay as you ride), or for multiple journeys (you buy 'minute bundles', to use when you need). For this study, you will receive a free bundle of 400 minutes to use over the next four weeks.

Beryl bikes are supported by Cornwall Council and are available in Truro, Falmouth, Penryn, Newquay, Penzance and St Austell.

To use Beryl bikes, you will need to download the 'Beryl' app from 'App Store' or 'Google Play' and then create an account, using the same email address you provide below.

If you have already registered with Beryl bikes - great! Please provide the same email address below that you used to register with Beryl bikes.

More information about Beryl bikes can be found here.

Q324 What is your email address?

(We will only use your email address to: 1. send you a code to claim your free bundle of Beryl bike minutes, 2. send you the weekly travel diaries, and 3. send you the £25 gift voucher at the end of the study)

Q325 We would like to use anonymised travel data from Beryl bikes about the study participants' journeys (e.g. average journey distance using a Beryl bike, average journey duration etc.). This data will not identify you in any way.

To collect this data, we will need to share your email address with Beryl bikes. If you do not consent to the University of Bath researchers sharing your email address with Beryl bikes, please opt out below. You can still participate in this study even if you opt out.

○ I **OPT OUT** to sharing my email address with Beryl bikes (1)

Debrief

Group D - Beryl bikes + Pen portraits
Thank you for completing the first survey.

For the next stage, you will receive free credits to use Beryl bikes. Beryl bikes are e-bikes for public use that you can hire for single journeys (pay as you ride), or for multiple journeys (you buy 'minute bundles', to use when you need). For this study, you will receive a free bundle of 400 minutes to use over the next four weeks.

Beryl bikes are supported by Cornwall Council and are available in Truro, Falmouth, Penryn, Newquay, Penzance and St Austell.

To use Beryl bikes, you will need to download the 'Beryl' app from 'App Store' or 'Google Play' and then create an account, using the same email address you provide below.

If you have already registered with Beryl bikes - great! Please provide the same email address below that you used to register with Beryl bikes.

More information about Beryl bikes can be found here.

Q358 What is your email address?
(We will only use your email address to: 1. send you a code to claim your free bundle of Beryl bike minutes, 2. send you the weekly travel diaries, and 3. send you the £25 gift voucher at the end of the study)

Q380 We would like to use anonymised travel data from Beryl bikes about the study participants' journeys (e.g. average journey distance using a Beryl bike, average journey duration etc.). This data will not identify you in any way.

To collect this data, we will need to share your email address with Beryl bikes. If you do not consent to the University of Bath researchers sharing your email address with Beryl bikes, please opt out below. You can still participate in this study even if you opt out.

- ○ I **OPT OUT** to sharing my email address with Beryl bikes (1)

Survey logic: present pen portraits tool

Please reflect on their story and answer the following questions:

Q410 How relevant did you find the scenario to your own life and travel needs?

- ○ Not at all relevant (1)
- ○ Not very relevant (2)
- ○ Somewhat relevant (3)
- ○ Very relevant (4)
- ○ Completely relevant (5)

Q411 To what extent did the scenario make you feel you could reduce your car use?

- ○ Not at all (1)
- ○ A little (2)
- ○ A moderate amount (3)
- ○ A lot (4)
- ○ Completely (5)
- ○ Not applicable / I don't use a car (6)

Q413 Did their story give you any ideas for how you might change how you travel in Cornwall, or reduce your need to travel?

- ○ Not at all (1)
- ○ A little (2)
- ○ A moderate amount (3)
- ○ A lot (4)

○ Completely (5)

Q415 Thank you! The next stage of this study is to complete a weekly travel diary - I'll send you the first travel diary in a week's time.

During the next four weeks, think about how you could reduce your car use. Please also consider what benefits or drawbacks you experience from making these changes.

Debrief

3. Travel diary protocol

The third appendix is the weekly travel diary. Research participants completed the travel diary for four consecutive weeks.

Block 1: Introduction

Travel diary - Week 1

Thank you for taking part in this study!

This travel diary will take approximately 3 minutes to complete.

Please click the arrow to start

Q15 What is your email address?
(We ask this to match your responses across all four travel diaries)

____

Q13 Did you use a Beryl bike this week?

- ○ Yes (1)
- ○ No (2)

Display logic: IF Q13 = NO, present block 2

Block 2: Reasons for non-use

Q18 Please tell us the reason(s) why you chose not to use a Beryl bike this week (please select all options that apply).

We ask this question to understand any barriers people living in Cornwall may experience to cycling or using a bike share scheme.

- ▢ I didn't need / want to travel by bike this week (1)
- ▢ I prefer to use my own bike, or walk (2)
- ▢ The weather was bad (3)
- ▢ There are no Beryl bike parking bays near where I live / work (4)
- ▢ There were no Beryl bikes available in the parking bays (5)

- [ ] I have a health condition or disability which prevents me from cycling (6)
- [ ] I don't feel safe cycling on roads with traffic (7)
- [ ] I don't feel confident in my cycling ability (8)
- [ ] I don't own any safety equipment (e.g. a bike helmet, hi-vis clothing) (9)
- [ ] I don't know how to use the Beryl bike scheme (10)
- [ ] I can't afford to use Beryl bikes (11)
- [ ] I've had a negative experience using Beryl bikes (please specify) (12) ____
- [ ] Other (please specify) (13) ____

Q32 Compared to a typical week, have you travelled more or less **this week** using the following ways to travel?

	A lot more (1)	A bit more (2)	About the same / no change (3)	A bit less (4)	A lot less (5)	Don't know / Not applicable (77)
Active travel (e.g. walking, cycling, wheeling) (Q32_1)	○	○	○	○	○	○
Public transport (Q32_2)	○	○	○	○	○	○
Sharing lifts with others (as the driver or as a passenger) (Q32_3)	○	○	○	○	○	○

Q20 Do you have any comments or suggestions about using Beryl bikes, or how the Council could support active ways to travel in Cornwall (e.g. walking, cycling, wheeling)?

____

____

Debrief

END OF SURVEY

Display logic: IF Q13 = YES, present block 3
Block 3: Journeys, purpose, mode shift

Q1 How many journeys did you make by Beryl bike on each day?

(There and back would count as **two** journeys)

	0 (6)	1 (1)	2 (2)	3 (3)	4 (4)	5 or more (5)
Monday (1)	○	○	○	○	○	○
Tuesday (2)	○	○	○	○	○	○
Wednesday (3)	○	○	○	○	○	○
Thursday (4)	○	○	○	○	○	○
Friday (5)	○	○	○	○	○	○
Saturday (6)	○	○	○	○	○	○
Sunday (7)	○	○	○	○	○	○

Display logic: days presented in Q5 based on the responses to Q1

Q5 In total, how far did you travel by Beryl bike each day?
(i.e. the **combined distance** travelled for **ALL** Beryl bike journeys on that day)

Distance in miles – 0 - 20

Monday ()

Tuesday ()

Wednesday ()

Thursday ()

Friday ()

Saturday ()

Sunday ()

Q29 What purpose(s) were your Beryl bike journeys for?
(Please select all options that apply for your Beryl bike journeys this week)

- ☐ Commuting to my place of work or study (1)
- ☐ The school run (2)
- ☐ Business-related travel (e.g. visiting clients, making deliveries) (3)
- ☐ Going to the shops, doctors, cinema etc. (4)
- ☐ Visiting family or friends (5)
- ☐ Leisure or exercise (6)
- ☐ Part of a journey using different travel modes (e.g. Beryl bike + public transport) (7)
- ☐ Other (8)

Q30 Before you started using Beryl bikes, which mode of transport would you have typically used for your journey(s)?
(Please select all options that apply for your Beryl bike journeys this week)

- ☐ Not applicable - I wouldn't have made the journey (1)

- [ ] My own vehicle (e.g. car / van / motorbike) (2)
- [ ] Public transport (3)
- [ ] My own bike / e-bike (4)
- [ ] I would walk (5)
- [ ] Someone would give me a lift (6)
- [ ] Other (7)

Q20 Do you have any comments or suggestions about using Beryl bikes, or how the Council could support active ways to travel in Cornwall (e.g. walking, cycling, wheeling)?

____

____

Debrief

END OF SURVEY

4. Survey 2 protocol (post-intervention)

The fourth appendix is the post-intervention survey.

This is Survey 2. Some questions may be similar to questions you previously answered in Survey 1 - this is intentional.

Please click the arrow to start

Block 1: Baseline travel behaviours

About how you travel

These questions are about how you commute to work and other travel you do (e.g. for shopping, visiting friends etc.).

Q111B In a typical week, how many journeys per week do you make to / from your place of work or education (i.e. **commuting**) using the following modes of transport?

	Number of commute journeys per week *(travelling there and back would count as two journeys)* (1)
N/A - I don't work or I work entirely from home *(just write 1 in the box)* (Q111_11)	
Walking,<br>or Wheeling (i.e. use a wheelchair) (Q111_1)	
Bicycle (including electric bike) (Q111_2)	

Scooter (including electric scooter) (Q111_3)	
Motorbike (Q111_4)	
Car / van (travelling alone) (Q111_5)	
Car / van (sharing lifts with others) (Q111_6)	
Car club (e.g. Co Cars) (Q111_7)	
Bus (Q111_8)	
Train (Q111_9)	
Other (please specify) (Q111_10)	

Q112B In a typical week, how many journeys per week do you make to / from other destinations (e.g. to the shops, visiting friends) using the following modes of transport?

	Number of non-work journeys per week *(travelling there and back would count as two journeys)* (1)
N/A - I tend to stay at home *(just write 1 in the box)* (Q112_11)	
Walking,<br>or Wheeling (i.e. use a wheelchair) (Q112_1)	
Bicycle (including electric bike) (Q112_2)	
Scooter (including electric scooter) (Q112_3)	
Motorbike (Q112_4)	
Car / van (travelling alone) (Q112_5)	
Car / van (sharing lifts with others) (Q112_6)	
Car club (e.g. Co Cars) (Q112_7)	

Bus (Q112_8)	
Train (Q112_9)	
Other (please specify) (Q112_10)	

Q331 B During the last month, how often did you use a car or van for short journeys (i.e. less than 3 miles)?

	N/A - I don't drive / own a car or van (99)	0 (8)	1 (1)	2 (2)	3 (3)	4 (4)	5 (5)	6 (6)	7 (7)
Number of days per week:	○	○	○	○	○	○	○	○	○

Q340 Does your household own **two or more** cars / vans?

○ Yes (1)

○ No - my household owns **one** car or van (2)

○ No - my household does not own a car or van (3)

Display logic: present Q341 if Q340 = Yes

Q341 To what extent would the following encourage your household to reduce your vehicle ownership to only one car / van?

	Not at all (1)	Not very much (2)	A little (3)	A lot (4)	Don't know / Not applicable (77)

Greater availability of car club vehicles (e.g. Co Cars) (Q341_1)	○	○	○	○	○
Improved cycle lane network (Q341_2)	○	○	○	○	○
Improved public transport (Q341_3)	○	○	○	○	○
Greater availability of Beryl bikes (Q341_4)	○	○	○	○	○
Greater availability of pool cars at work (i.e. use of company cars or vans by employees) (Q341_5)	○	○	○	○	○

Q342 To what extent would you support a 'workplace parking levy'?

A Workplace Parking Levy is a Council charge on employers who provide workplace car parking, with the money to be invested in improving public transport and the cycle lane network in Cornwall.

Employers can decide whether to absorb the cost or pass it on to their employees who use the parking spaces.

○ Strongly oppose (1)

◯ Tend to oppose (2)

◯ Neither oppose nor support (3)

◯ Tend to support (4)

◯ Strongly support (5)

Block 2: Active travel

Q381 During the last week, how many hours did you spend on each of the following activities?
(Please move the slider into the correct position)

Number of hours

0 1 2 3 4 5 6 7 8 9 10

Physical exercise such as swimming, jogging, aerobics, football, tennis, gym workout etc. ()

Cycling, including cycling to work and during leisure time ()

Walking, or **Wheeling** (i.e. use a wheelchair), including walking / wheeling to work, shopping, for pleasure etc. ()

Block 3: e-bikes

About e-bikes and bike share

You're doing great! These questions are about your views on e-bikes and bike share schemes (e.g. Beryl bikes).

Q285 B How likely is your household to buy an e-bike (or another e-bike) in the next 12 months?

◯ Very unlikely (1)

◯ Somewhat unlikely (2)

◯ Neither likely nor unlikely (3)

◯ Somewhat likely (4)

○ Very likely (5)

○ Don't know (77)

Q592 To what extent do you agree with the following statement?
My household could easily afford to buy e-bikes for everyone in the household

○ Strongly disagree (1)

○ Somewhat disagree (2)

○ Neither agree nor disagree (3)

○ Somewhat agree (4)

○ Strongly agree (5)

○ Don't know / Not applicable (77)

Q267 B To what extent do you agree with the following statements about Beryl bikes?

	Strongly disagree (1)	Somewhat disagree (2)	Neither agree nor disagree (3)	Somewhat agree (4)	Strongly agree (5)
It's becoming more common for people in my town to use Beryl bikes (Q267 B_1)	○	○	○	○	○
I'm aware that people where I live and work use Beryl bikes (Q267 B_2)	○	○	○	○	○

Q266 Since hearing about the Beryl bike scheme in Cornwall, have you recommended Beryl bikes to anyone else?

○ Yes (please indicate how many people) (1) ____

○ No (2)

Q268 Has anyone recommended Beryl bikes to you?

○ Yes (please indicate how many people) (1) ____

○ No (2)

Q302 B To what extent do you agree with the following statements about how using Beryl bikes may help you personally? (Part A)

	Strongly disagree (1)	Somewhat disagree (2)	Neither agree nor disagree (3)	Somewhat agree (4)	Strongly agree (5)	Not applicable (99)
It will make my trips quicker (Q302 B_1)	○	○	○	○	○	○
It will make my trips easier (Q302 B_2)	○	○	○	○	○	○
I will be able to cycle longer distances (Q302 B_3)	○	○	○	○	○	○
I can avoid fatigue or getting sweaty before work or socialising (Q302 B_4)	○	○	○	○	○	○

It will help me reduce my carbon footprint (Q302 B_5)	○	○	○	○	○	○

Q301 B To what extent do you agree with the following statements about how using Beryl bikes may help you personally? (Part B)

	Strongly disagree (1)	Somewhat disagree (2)	Neither agree nor disagree (3)	Somewhat agree (4)	Strongly agree (5)	Not applicable (99)
It will provide me with exercise (Q301 B_6)	○	○	○	○	○	○
It will provide me with mental health benefits (Q301 B_7)	○	○	○	○	○	○
It will enable me to cycle with friends / family as a group (Q301 B_8)	○	○	○	○	○	○
It will take away concerns around bike maintenance and storage (Q301 B_9)	○	○	○	○	○	○
It will take away the worry of	○	○	○	○	○	○

bike theft (Q301 B_10)

Q300 B To what extent do you agree with the following statements about how using Beryl bikes may help you personally? (Part C)

	Strongly disagree (1)	Somewhat disagree (2)	Neither agree nor disagree (3)	Somewhat agree (4)	Strongly agree (5)	Not applicable (99)
It will save me money (Q300 B_11)	○	○	○	○	○	○
It will connect me to places not served by public transport (Q300 B_12)	○	○	○	○	○	○
I will be able to use my car less (Q300 B_13)	○	○	○	○	○	○
I will avoid traffic congestion / parking difficulties (Q300 B_14)	○	○	○	○	○	○
I can try an e-bike before I decide whether to	○	○	○	○	○	○

buy one
(Q300
B_15)

Routing question – block 4 or block 5

Q556 Did you use a Beryl bike during the last **month**?

- ◯ Yes (1)
- ◯ No (2)

Block 4: Evaluation of Beryl bikes - not used this week (IF Q556 = Yes)

Q555 Did hiring a Beryl bike encourage you to...

- ◯ cycle for the first time ever? (1)
- ◯ cycle for the first time after a long break (5 years or more)? (2)
- ◯ cycle for the first time after a shorter break (less than 5 years)? (3)
- ◯ No change, I was already cycling (4)
- ◯ Don't know / Not applicable (77)

Q505 During the last month, did you combine Beryl bikes with any other mode of transport for a journey? (e.g. after a train or bus trip)

Please select all that apply

- ▢ ⊗No, I did not combine Beryl bikes with another mode of transport (1)
- ▢ Bus (2)
- ▢ Train (3)
- ▢ Taxi (4)
- ▢ Car / van as a driver (5)
- ▢ Car / van as a passenger (6)
- ▢ E-scooter / scooter / motorcycle (7)

☐ Other (please specify) (8) ____

Q519 Please rate your experience of the following aspects of using Beryl bikes (Part A):

	Very dissatisfied (1)	Somewhat dissatisfied (2)	Neither satisfied nor dissatisfied (3)	Somewhat satisfied (4)	Very satisfied (5)	Don't know / Not applicable (77)
Bike comfort (Q519_1)	o	o	o	o	o	o
Lights (Q519_2)	o	o	o	o	o	o
Brakes (Q519_3)	o	o	o	o	o	o
Gears (Q519_4)	o	o	o	o	o	o
Basket carrying capacity (if applicable) (Q519_5)	o	o	o	o	o	o

Q537 Please rate your experience of the following aspects of using Beryl bikes (Part B):

	Very dissatisfied (1)	Somewhat dissatisfied (2)	Neither satisfied nor dissatisfied (3)	Somewhat satisfied (4)	Very satisfied (5)	Don't know / Not applicable (77)
Bike battery charge (Q537_6)	o	o	o	o	o	o
Bike locking / unlocking process (Q537_7)	o	o	o	o	o	o

Availability of bikes in the parking bays (Q537_8)	◯	◯	◯	◯	◯	◯
Location of the parking bays (Q537_9)	◯	◯	◯	◯	◯	◯
Price (Q537_10)	◯	◯	◯	◯	◯	◯

Q528 Please rate your experience of the following aspects of using Beryl bikes (Part C):

	Very dissatisfied (1)	Somewhat dissatisfied (2)	Neither satisfied nor dissatisfied (3)	Somewhat satisfied (4)	Very satisfied (5)	Don't know / Not applicable (77)
Customer service (Q528_11)	◯	◯	◯	◯	◯	◯
Registration process (Q528_12)	◯	◯	◯	◯	◯	◯
Ease of use - Beryl app (Q528_13)	◯	◯	◯	◯	◯	◯
Communications (e.g. emails, social media) (Q528_14)	◯	◯	◯	◯	◯	◯

Block 5: Evaluation of Beryl bikes - used this week (IF Q556 = No)

Q62 Did hiring a Beryl bike encourage you to...

◯ cycle for the first time ever? (1)

◯ cycle for the first time after a long break (5 years or more)? (2)

- cycle for the first time after a shorter break (less than 5 years)? (3)
- No change, I was already cycling (4)
- Don't know / Not applicable (77)

Q63 During the last month, did you combine Beryl bikes with any other mode of transport for a journey? (e.g. after a train or bus trip)

Please select all that apply

- ⊗No, I did not combine Beryl bikes with another mode of transport (1)
- Bus (2)
- Train (3)
- Taxi (4)
- Car / van as a driver (5)
- Car / van as a passenger (6)
- E-scooter / scooter / motorcycle (7)
- Other (please specify) (8) ____

Q64 Please rate your experience of the following aspects of using Beryl bikes (Part A):

	Very dissatisfied (1)	Somewhat dissatisfied (2)	Neither satisfied nor dissatisfied (3)	Somewhat satisfied (4)	Very satisfied (5)	Don't know / Not applicable (77)
Bike comfort (Q64_1)	○	○	○	○	○	○
Lights (Q64_2)	○	○	○	○	○	○
Brakes (Q64_3)	○	○	○	○	○	○

Gears (Q64_4)	○	○	○	○	○	○
Basket carrying capacity (if applicable) (Q64_5)	○	○	○	○	○	○

Q65 Please rate your experience of the following aspects of using Beryl bikes (Part B):

	Very dissatisfied (1)	Somewhat dissatisfied (2)	Neither satisfied nor dissatisfied (3)	Somewhat satisfied (4)	Very satisfied (5)	Don't know / Not applicable (77)
Bike battery charge (Q65_6)	○	○	○	○	○	○
Bike locking / unlocking process (Q65_7)	○	○	○	○	○	○
Availability of bikes in the parking bays (Q65_8)	○	○	○	○	○	○
Location of the parking bays (Q65_9)	○	○	○	○	○	○
Price (Q65_10)	○	○	○	○	○	○

Q66 Please rate your experience of the following aspects of using Beryl bikes (Part C):

	Very dissatisfied (1)	Somewhat dissatisfied (2)	Neither satisfied nor dissatisfied (3)	Somewhat satisfied (4)	Very satisfied (5)	Don't know / Not applicable (77)
Customer service (Q66_11)	○	○	○	○	○	○
Registration process (Q66_12)	○	○	○	○	○	○
Ease of use - Beryl app (Q66_13)	○	○	○	○	○	○
Communications (e.g. emails, social media) (Q66_14)	○	○	○	○	○	○

Block 6: Barriers to Beryl bike use

Q506 What do you think are **the three main barriers** to people using Beryl bikes in Cornwall?
(Please select **three** options from the list below)

- ☐ Lack of awareness about Beryl bikes (1)
- ☐ Lack of availability of bikes in parking bays (2)
- ☐ Location of parking bays (3)
- ☐ Cost of using Beryl bikes (4)
- ☐ Beryl bike design / comfort (5)
- ☐ Beryl bike reliability / battery charge (6)
- ☐ Personal safety / busy roads / lack of safe cycling routes (7)
- ☐ Lack of cycling confidence or competence (8)
- ☐ Long distances / steep hills (9)

Q317 Thank you for your responses.

Would you like to receive a £25 Love2Shop e-giftcard for participating in this study?

- ◯ Yes (1)
- ◯ No (2)

Debrief

Thank you very much for taking part in this study!

Further information

This study is a collaboration between Cornwall Council and researchers at the University of Bath. The aim of the study is to explore the travel behaviours and perceptions of people living in Cornwall. This information will be used to inform Cornwall Council travel policies or interventions to reduce carbon emissions.

If you have any questions about the study, please contact the research team: Mark Wilson (mw2640@bath.ac.uk) or Lorraine Whitmarsh (lw2253@bath.ac.uk).

If you have concerns about your participation in this study or you wish to make a complaint, please contact the Department of Psychology Research Ethics Committee: (psychology-ethics@bath.ac.uk; +44 (0)1225 384714). The PREC reference number for this study is: 23 - 079.

Privacy Notice: Your data will be used only for the purposes set out in the information sheet. Your consent is conditional upon the University complying with its duties and obligations under current UK data protection legislation. The University of Bath privacy notice can be found here.

Please CLICK THE ARROW BELOW to submit your responses

5. Mode shift emission reduction calculations

The fifth appendix presents the calculations of the emission reduction due to mode shift, from single occupancy car to Beryl bike.

Quantifying emission reduction is complex for several reasons: different vehicles emit different levels of CO_2 per km (depending on the model, fuel type, age, and maintenance of the vehicle), road conditions and topography vary, and driver behaviour in terms of fuel efficiency also varies. Furthermore, different studies apply different system boundaries when conducting their Life Cycle Analysis, such as including or excluding the emissions from vehicle manufacture. It is therefore very difficult to provide a precise figure for reduction of CO_2 equivalent per km (CO_2e per km), which can be attributed to mode shift, in an intervention study in real world settings. The calculation below combines empirical data collected in this study with existing Life Cycle Analysis literature to provide an emission reduction *range*[1], rather than a single emission reduction *figure*. The actual emission reduction due to mode shift in this study likely falls within this range or uncertainty space.

The following assumptions were applied in this quantification:

- Most households in Cornwall own a petrol or diesel car powered only by an internal combustion engine (ICE), rather than a hybrid or electric vehicle[2]. The carbon emissions from a petrol or diesel ICE car are therefore used as the baseline in this calculation.
- The emissions from using an ICE car in Cornwall are assumed to be comparable with the emissions from ICE vehicles used in the studies and government sources referenced below.
- The emissions from using a Beryl bike in Cornwall are assumed to be comparable with the emissions from using e-bikes or e-bike shared mobility in the studies referenced below.
- Beryl bike journeys are assumed to substitute a single occupancy car journey. There were few cases of Beryl bikes substituting for lift share (see Figure 4 in the article).

Quantification of mode shift emission reduction, single occupancy car to Beryl bike:

- Petrol/diesel cars have an emission intensity between **122.1 – 210.5** g CO_2 per km[3].

---

[1] This methodology to provide a range for emission reduction/increase has been used in other studies, for example: Wilson et al. (2020). Potential Climate Benefits of Digital Consumer Innovations. *Annual Review of Environment and Resources*, 45, 113-144.

[2] In this study, 79.5% of residents and 85.7% of Council staff own a petrol or diesel car. This is comparable with a previous study of Cornwall residents, which found 80.0% own a petrol or diesel car. See: Wilson and Whitmarsh (2023). Cornwall Council behaviour change and engagement programme – survey of residents. CAST report for Cornwall Council.

[3] These articles were used to provide the petrol/diesel car emission intensity range:

— E-bikes and e-bike share have an emission intensity between **14.8 – 74.0** g CO_2e per km[4].

— If e-bike substitutes a car journey, the difference in emission intensity between petrol/diesel cars and e-bikes provides an emission reduction range of **48.1 – 195.7** g CO_2e *per km*[5].

— In this study, the average distance of a Beryl bike journey was between 2.0 – 3.2 km. Multiplying these distances by the emission reduction per km provides an emission reduction range of **96.2 – 626.2** g CO_2e *per journey*[6].

— In this study, the participants made, on average, between 0.8 – 1.5 Beryl bike journeys per week. Multiplying the weekly journey frequency by 52 weeks equals 41.6 – 78.0 Beryl bike journeys per person per year. Multiplying the number of annual journeys by the emission

---

1) European Parliament (2019). https://www.europarl.europa.eu/news/en/headlines/society/20190313STO31218/co2-emissions-from-cars-facts-and-figures-infographics. They estimate **122.1** g CO_2 per km for passenger cars.
2) Department for Transport (2023). Journey emission comparisons: October 2023: interactive dashboard (dft.gov.uk). Emissions vary depending on the journey, but a short journey from Sunderland to Newcastle (14.3 miles, or 23.0 km) would equate to **139.6** g CO_2 per km for an average petrol car and **140.9** g CO_2 per km for an average diesel car.
3) NimbleFins (2023). Average CO2 Emissions per Car in the UK | NimbleFins. They estimate **138.4** g CO_2 per km for the average car.
4) O'Driscoll et al. (2018). Real world CO_2 and NOx emissions from 149 Euro 5 and 6 diesel, gasoline and hybrid passenger cars. *Science of The Total Environment*, 621, 282-290, https://doi.org/10.1016/j.scitotenv.2017.11.271. They found, for urban driving, **210.5** g CO_2 per km for an average petrol car and **170.2** g CO_2 per km for an average diesel car.

[4] These articles were used to provide the e-bike emission intensity range:
1) Stot (2020). How green is cycling? Riding, walking, ebikes and driving ranked - BikeRadar. *Bike Radar* [online]. He estimates **14.8** g CO_2e per km for private e-bikes.
2) Zhou et al. (2023). Mode substitution and carbon emission impacts of electric bike sharing systems. *Sustainable Cities and Society,* 89, 104312, https://doi.org/10.1016/j.scs.2022.104312. They estimate **19.47** g CO_2e per km for e-bike shared mobility.
3) Philips et al. (2022). E-bikes and their capability to reduce car CO_2 emissions. *Transport Policy*, 116, 11-23, https://doi.org/10.1016/j.tranpol.2021.11.019. They estimate **22.0** g CO_2e per km for private e-bikes.
4) Brand et al. (2022). Chapter Eleven - Cycling, climate change and air pollution. In: E. Heinen & T. Götschi (eds.), *Advances in Transport Policy and Planning*. Academic Press, 10, 235-264, https://doi.org/10.1016/bs.atpp.2022.04.010. They estimate between **15.0 – 25.0** g CO_2e per km for private e-bikes (excluding the emissions associated with providing infrastructure such as roads and cycle paths). They estimate the emissions from a shared e-bike system can be as high as **74.0** g CO_2e per km (excluding the infrastructure component), although this will vary depending on whether or not the bikes require collection and redistribution. This is based on analysis by OECD/ITF 2020. Good to Go? Assessing the Environmental Performance of New Mobility, https://www.itf-oecd.org/good-go-assessing-environmental-performance-new-mobility. Paris: International Transport Forum, OECD Publishing.

[5] The *per km* emission reduction range is calculated by: 1) subtracting the highest emission per km of e-bikes/e-bike share from the lowest emission per km for petrol/diesel cars (i.e., low estimate = 122.1 - 74.0 = **48.1** g CO_2e per km), and 2) subtracting the lowest emission per km of e-bikes/e-bike share from the highest emission per km for petrol/diesel cars (i.e., high estimate = 210.5 - 14.8 = **195.7** g CO_2e per km).
This is broadly similar to Li et al. (2023), who found an emission reduction range of **108 – 120** g CO_2e per km for e-bike shared mobility, compared to car journeys. See: Li et el. (2023). Do shared E-bikes reduce urban carbon emissions? *Journal of Transport Geography*, 112, 103697, https://doi.org/10.1016/j.jtrangeo.2023.103697.

[6] The *per journey* emission reduction range is calculated by: 1) multiplying the low estimate emission reduction by the short average distance (i.e., 48.1 g CO_2e per km * 2.0 km = **96.2** g CO_2e per journey), and 2) the high estimate emission reduction by the long average distance (i.e., 195.7 g CO_2e per km * 3.2 km = **626.2** g CO_2e per journey)

reduction per journey equals 4001.9 – 48846.7 g CO_2e per person per year (for the scenario of 100% private car substitution[7]).

— In this study, 27.9% of Beryl bike journeys substituted for private car use. Multiplying the emission reduction per person per year by .279 equals 1116.5 – 13628.2 g CO_2e *per person per year*.

— Thus, for the participants in this study, the annual emission reduction due to mode shift from single occupancy car to Beryl bike is **1.1 – 13.6** kg CO_2e *per person per year*.

— For context, 13.6 kg CO_2e per person per year is equivalent to 1.2% of the annual travel carbon footprint of a Cornwall resident[8].

---

[7] The *per person per year* emission reduction range is calculated by: 1) multiplying the low annual frequency by the low emission reduction per journey (i.e., low estimate = 41.6 annual journeys * 96.20 g CO_2e per journey = 4001.9 g CO_2e per person per year), and 2) multiplying the high annual frequency by the high emission reduction per journey (i.e., high estimate = 78.0 annual journeys * 626.24 g CO_2e per journey = 48846.7 g CO_2e per person per year). This maximum range would reflect 100% Beryl bike substitution for car.

[8] 13.6 kg CO_2e per person per year is 1.2% of the combined annual road and rail transport emissions of a Cornwall resident (road transport emissions is 23% of the total carbon footprint of a Cornwall resident, whereas rail transport accounts for 0.75%; see Cornwall Sector emissions: The Carbon Neutral Challenge - Cornwall Council). The carbon emissions from aviation (1.5% of the total carbon footprint) and marine navigation (1.75%) are excluded from this calculation, as e-bike shared mobility cannot substitute for these travel modes. This calculation assumes the carbon footprint of the average Cornwall resident is identical to the UK per capita carbon footprint of 4.7 tonnes of CO_2 per year, see: United Kingdom: CO2 Country Profile - Our World in Data.

6. Residents' perceptions of Beryl bikes, pre- and post-intervention

The sixth appendix is the results of paired samples t-tests to measure changes in residents' perceptions of Beryl bikes over the study period. These findings are not statistically significant.

Table 1, Within-group analyses of residents' perceptions of Beryl bikes (paired samples t-test)

Attribute of Beryl bikes	Data coll. time point	M	SD	MD	df	t	*p*	95% CI Low	95% CI High
Make trips quicker	Pre-intervention	3.01	1.324	.11	118	.670	.504	-.214	.432
	Post-intervention	3.12	1.348						
Make trips easier	Pre-intervention	2.94	1.242	.03	121	.213	.832	-.272	.338
	Post-intervention	2.98	1.256						
Able to cycle longer distances	Pre-intervention	3.23	1.330	-.05	117	-.284	.777	-.405	.303
	Post-intervention	3.18	1.344						
Avoid fatigue or getting sweaty before work or socializing	Pre-intervention	3.14	1.213	.06	114	.393	.695	-.246	.368
	Post-intervention	3.20	1.171						
Reduce carbon footprint	Pre-intervention	3.58	1.310	.12	121	.704	.483	-.208	.438
	Post-intervention	3.70	1.212						
Provide exercise	Pre-intervention	3.62	1.199	-.04	120	-.283	.777	-.330	.247
	Post-intervention	3.58	1.153						
Provide mental health benefits	Pre-intervention	3.56	1.148	-.01	116	-.121	.904	-.297	.263
	Post-intervention	3.55	1.148						
Enable cycling with friends / family as a group	Pre-intervention	2.84	1.206	.18	115	1.122	.264	-.139	.501
	Post-intervention	3.03	1.161						
Reduce concern about bike	Pre-intervention	3.59	1.149	.11	119	.738	.462	-.182	.399

Attribute of Beryl bikes	Data coll. time point	M	SD	MD	df	t	*p*	95% CI Low	High
maintenance and storage	Post-intervention	3.70	1.097						
Reduce concern about bike theft	Pre-intervention	3.49	1.167	.21	119	1.428	.156	-.080	.497
	Post-intervention	3.70	1.120						
Save money	Pre-intervention	2.52	1.151	.13	121	.816	.416	-.175	.421
	Post-intervention	2.65	1.105						
Connect to places not served by public transport	Pre-intervention	3.45	1.162	-.15	118	-.899	.370	-.458	.172
	Post-intervention	3.30	1.252						
Use car less	Pre-intervention	3.00	1.299	.25	96	1.352	.180	-.116	.611
	Post-intervention	3.25	1.118						
Avoid traffic congestion / parking difficulties	Pre-intervention	3.40	1.119	.11	113	.682	.497	-.200	.411
	Post-intervention	3.51	1.131						
Try an e-bike before deciding to buy one	Pre-intervention	3.85	1.207	.18	104	1.212	.228	-.115	.477
	Post-intervention	4.03	1.023						

7. Council staff perceptions of Beryl bikes, post-intervention

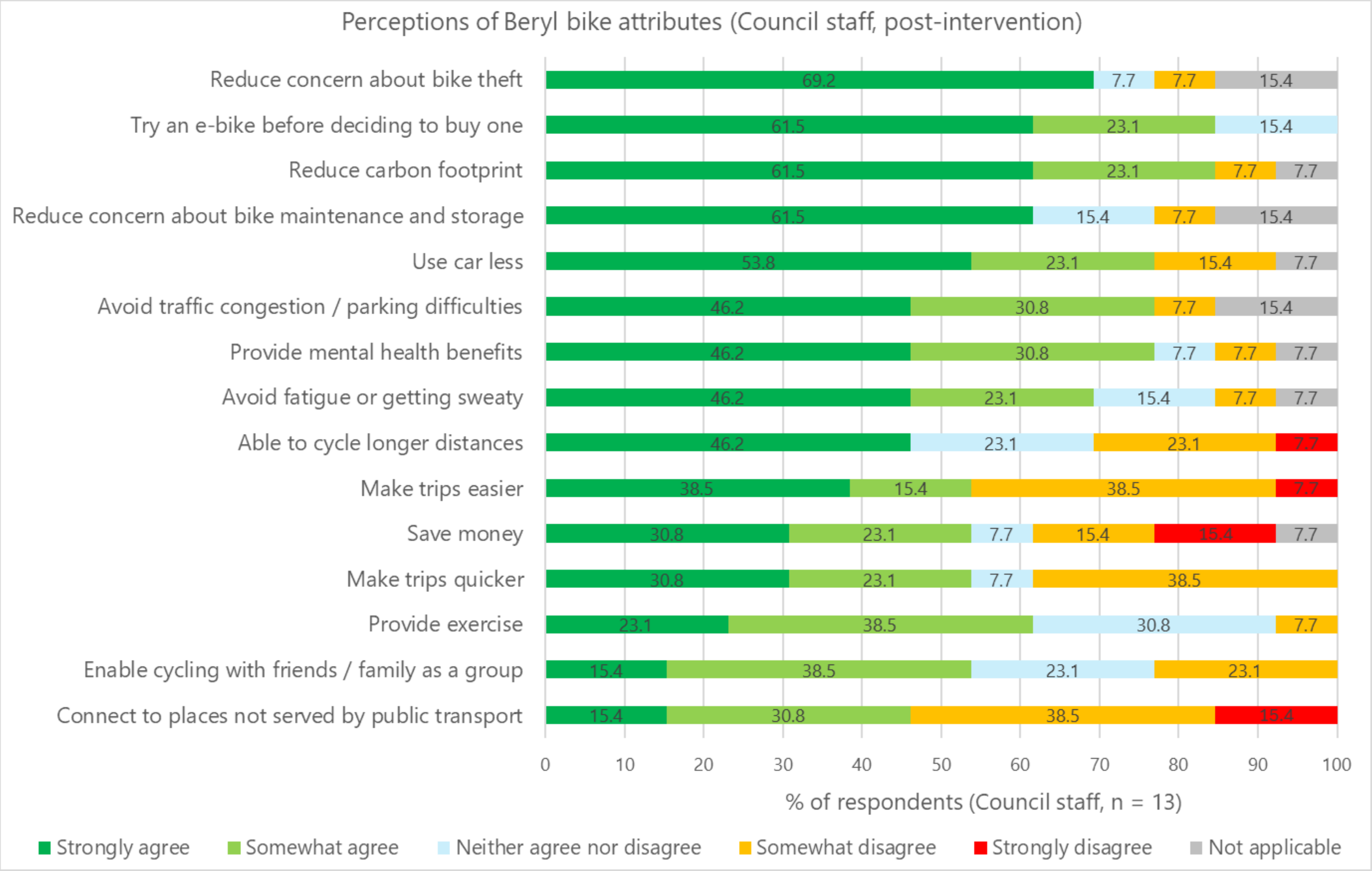

Figure 1, Council staff's perceptions of Beryl bike attributes (post-intervention)

8. Attrition rate over the intervention period

The eighth appendix shows the number of participants at each data collection time point during the intervention. For both residents and Council staff, the highest rate of drop-out occurred when we asked the participants to complete the first weekly travel diary.

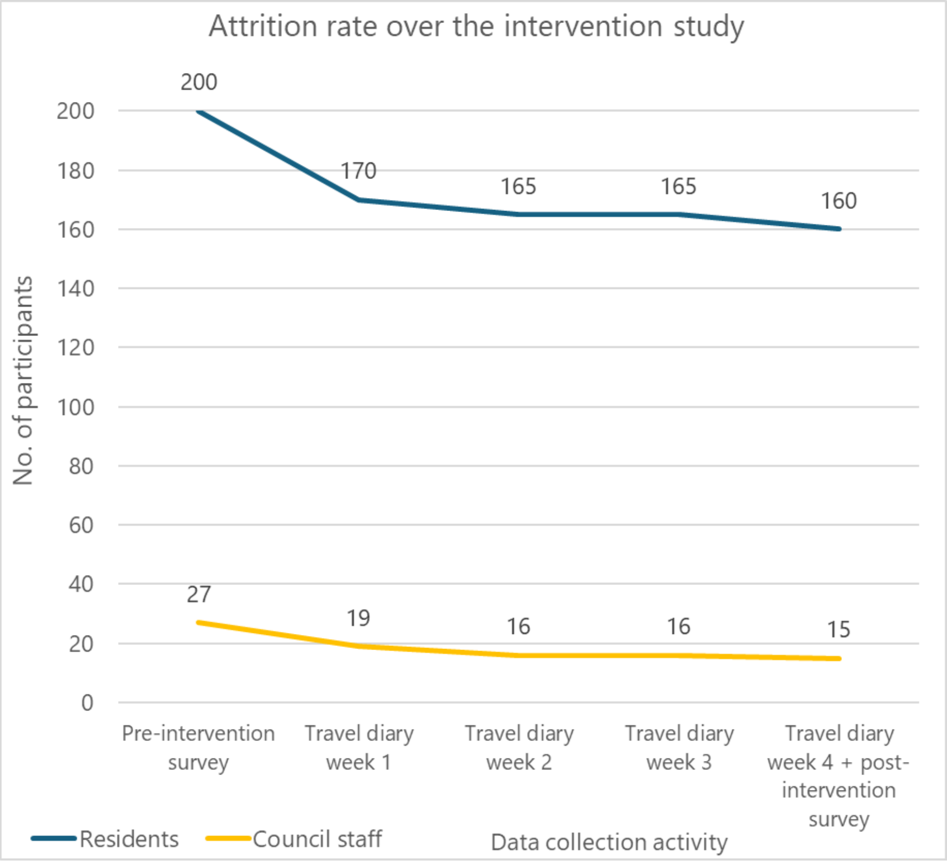

Figure 2, Attrition rate over time

Please note, one Council staff response and nine resident responses were removed from the final data set due to data quality issues (i.e., incomplete responses, straight-lining). Thus, the final number of participants included in the study were: Council staff n=14; Residents n=151.

9. Access to Beryl bikes

The ninth appendix presents data for the participants' access to Beryl bikes. Participants were asked in which towns or city they are most likely to use Beryl bikes (they could select multiple locations). Truro and Falmouth were the most frequently selected locations (Table 3).

Table 2, Locations where participants are most likely to use Beryl bikes

	Residents* (%)	Council staff (%)
Truro	41.7	64.3
Falmouth	28.5	21.4
Newquay	19.2	28.6
Penryn	15.2	21.4
St Austell	15.2	7.1
Penzance	12.6	28.6
Not applicable	15.9	0.0

* Participants could select multiple options and so the columns do not total 100%.

Participants were then asked about the proximity of Beryl bike parking bays to their home, place of work or education, and public transport hubs such as a train station or bus station (Table 4). There is good coverage of parking bays near participants' homes, with 56.3% of residents and 85.7% of Council staff reporting a parking bay within walking distance. There is currently less coverage close to public transport hubs or participants' places of work or education.

Table 3, Accessibility of Beryl bikes

Proximity of Beryl bike parking bays	Yes (%)	No (%)	Don't know / N/A (%)
Percentage of residents			
Beryl bike parking bay within walking distance of home	56.3	27.8	15.9
Beryl bike parking bay within walking distance of your place of work or education	31.1	28.5	40.4
Beryl bike parking bay within walking distance of key public transport hubs (if you use public transport)	37.7	6.0	56.3
Percentage of Council staff			
Beryl bike parking bay within walking distance of home	85.7	14.3	0.0
Beryl bike parking bay within walking distance of your place of work or education	71.4	7.1	21.5
Beryl bike parking bay within walking distance of key public transport hubs (if you use public transport)	22.2	0.0	77.8

10. Measuring change in physical activity, pre- and post-intervention

The tenth appendix presents empirical data for changes in the participants' physical activity level during the study. This was investigated to determine whether increased active travel may encourage increased physical exercise as a health spillover effect. Participants were asked how many hours per week they spend doing three types of physical activity[9]:

- Exercise (such as swimming, jogging, aerobics, football, tennis, gym workout etc.)
- Cycling (including cycling to work and during leisure time)
- Walking or wheeling (including to work, shopping, for pleasure etc.)

Figure 3 shows residents and Council staff increased the duration of all three types of physical activity over the study period[10]. The increase in Council employees' time spent walking or wheeling is statistically significant[11]. Further analysis found no statistically significant differences in physical activity before and after the intervention for the four intervention groups, nor for Beryl bike users (paired samples t-tests).

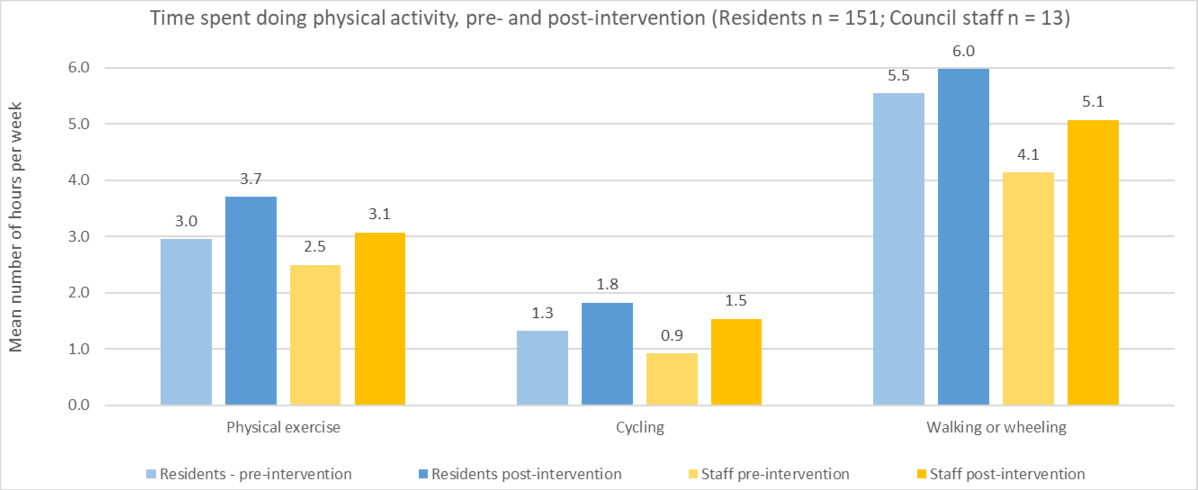

Figure 3, Weekly duration of physical activity during the study period

---

[9] This question was adapted from the General Practice Physical Activity Questionnaire, a screening tool commonly used in routine general practice to provide a simple physical activity index. See: National Health Service (2013), General practice physical activity questionnaire (GPPAQ) - GOV.UK (www.gov.uk).

[10] It is not possible to directly compare residents and Council staff in terms of the number of hours per week, as they were presented with slightly different scales. The scale used for residents was '0 – 10' hours per week, whereas the Council staff scale was '0 – 6 or more' hours per week. This may decrease the Council staff mean, relative to residents.

[11] A paired samples t-test revealed Council employees' weekly duration of walking or wheeling increased in the post-intervention survey (5.08 ± 1.80), compared to the weekly duration in the pre-intervention survey (4.00 ± 1.83), a statistically significant increase of 1.08 (95% CI, .242 to 1.91), $t(12) = 2.809$, $p = .016$.

11. Study 1 - focus group protocol

The eleventh appendix is the focus group protocol. The findings from the focus groups were used to inform the focus and design of the behaviour change intervention in Study 2.

Overview of focus group activities

Activity	Focus	Duration
Introduction	Present focus group content, emphasise confidentiality	4 mins
Activity 1 – Current travel behaviours and potential modal shift	*Scene-setter: match travel modes with typical everyday journeys*<br>Benefits/disadvantages of different modes<br>Barriers/enablers of modal shift (including group-specific questions)	20 mins
Activity 2 – Council policies	*Scene-setter: ranking exercise of policies*<br>Discussion of pull and push mechanisms	15 mins
Activity 3 – Visioning reduced car use	Agency, lifestyles & habits, social norms & values	15 mins
Wrap up and debrief		1 min
Exit Survey	8 questions: Car ownership, urban/rural area, Sociodemographic information.<br>On paper if in-person, or via a link in the chat if online	2 mins

Preamble and introductions (4 min)

Hi everyone, thank you all so much for coming, my name is Mark and I'm from the Centre for Climate Change and Social Transformations (CAST) at the University of Bath.

We are working with Cornwall Council to understand how people in Cornwall travel and what you think about different travel options. There's no right or wrong answers, so please don't feel pressured to answer in a certain way. We realise there are lots of challenges and reasons for the way we travel. We want to know how you experience getting around in Cornwall – this will inform the Council's approach to travel policies or interventions in the future.

Ground rules - respect and confidentiality

Before we start, two important requests. We ask that everyone here is treated with respect, we want to hear what everyone has to say.

We also ask that you do not discuss who participated in this focus group with anyone else, or repeat what they say. This is so everyone can freely express their views. Everything you say is

confidential and your name will not be attached to your quotes in any reports or research outputs.

Thank you for agreeing to these requests. Does anyone have any questions before we start the recording?
Start recording.

First of all, we'd like you all to get to know each other. So could you say your name, where you live, and what you like most about living in Cornwall.
Participants introduce themselves.

That's great. So, to briefly give you an idea of what is to come, we will do three activities. Each activity will last about 15-20 minutes and each one considers a different aspect of travel behaviour.

Activity 1 - Current travel behaviours & the potential for modal shift (20 mins)

Instructions
Let's start by talking about how you usually travel on a day-to-day basis.
Switch to Presentation mode – match four types of frequent journey to most common mode of travel.

On the screen you can see six blue images which indicate six different ways to travel in Cornwall. These are:

1. Your own car, or a family car if you share it
2. Public transport
3. Walking, cycling or wheeling, sometimes referred to as 'active travel'
4. Car sharing – this could be:
    a. a car club - which is a fleet of shared vehicles which you can book on a 'pay as you go basis', such as Co Cars
    b. a website where you offer or receive lifts, such as Liftshare or CarShare Cornwall
    c. or informal car sharing - basically giving your friend or colleague a lift.
5. Electric vehicle – this is different from image 1 which refers only to a petrol, diesel or hybrid car
6. Multi-modal travel – perhaps some of your frequent journeys involve more than one form of transport. 'Park and Ride' is an example of multi-modal travel

In the green box at the bottom of the screen, you can see four common types of journey: A) is commuting to your place of work or study, B) is going to the shops, and so on.

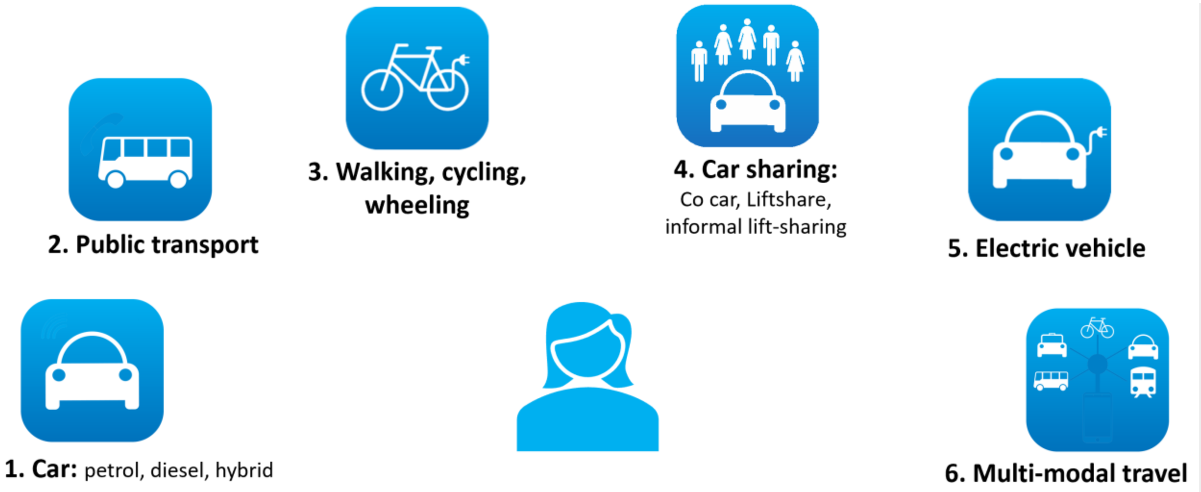

**A)** Commuting (to place of work/education)	**C)** Leisure or visiting family/friends
**B)** Shopping or access to services (e.g. supermarket, doctors, library…)	**D)** The school run (if applicable)

What I'd like you to do is for each type of journey - in the green box - think about the mode of transport you most frequently use for that journey - the blue image.

General Questions

Check if 'school run' is relevant, depending on the respondent group.

Close Presenter mode after a minute.

1. Let's start with commuting – which mode of travel do you normally use to get to work?
    a. Why do you choose this way to travel?
    b. Approximately how far is the journey to work, in miles?

2. Is there a difference between how you travel to get to work (commuting) and how you travel for these other reasons, such as going to the shops, or visiting friends?
    a. *If yes:* Why is this?
    b. Approximately how far is the journey to _______, in miles?

3. Does your choice of how you get around depend on the distance you have to travel?
    a. For example, do you use a different mode of travel for shorter journeys, less than 2 or 3 miles, compared to longer journeys?
    b. *If yes:* Why is this?

4. Do you see your choice of travel as a compromise?
    a. *Prompt:* Perhaps you save money travelling this way, but it's less convenient?

Go to questions on travel mode 1 – 6, depending on respondents' answers

Group-specific questions

1) Private car
1. What are the **advantages** of driving your own/family car, compared to other ways of getting around?

2. What are the **disadvantages** of driving your own/family car, compared to other ways of getting around?

2) Public Transport
1. What are the **advantages** of using public transport, compared to using a car?

2. What are the **disadvantages** of using public transport, compared to using a car?

3. What would **enable** you to use public transport more?
    a. *Prompt:* I would use public transport more if...

4. Do you have enough information about public transport services in your area?
    a. *If no:* Do you have any suggestions for how the Council, or Public Transport Service Providers, could support you to access this information?

5. Are there any **barriers** which prevent or deter you from using public transport more?
    a. *If yes:* What are they and how do they affect you?

Service provision

b. Are the ________ services in your area frequent enough for you to get to work, or to access local shops and services?
c. Are the _______ services in your area reliable?
d. Are there any gaps in public transport services – do the bus routes go to the places you need to get to?
e. *If very negative:* Could you tell me the bus service, where it goes from and to, so I can pass this feedback to the Council?
    i. Bus service number?

f. Do you have to change services, for example change buses or from bus to train, for your journey?
    i. *If yes:* How do you find the connectivity between the services? Are you waiting for a long time? Do you have to walk far to change services?

Bus stops

g. Are there enough bus stops on the routes you use?
h. Are the bus stops comfortable and clean? For example, is there a roof to shelter under if it rains, or gets very hot?
i. In general, do you find the electronic displays at bus stops provide accurate information?

Safety

j. Do you feel safe when using public transport? For example, do you encounter anti-social behaviour from other users?

Cost

6. What do you think of the cost of public transport fares?
    a. *If expensive:* Does this affect how often you choose to use the bus or train?

3) Walking, cycling or wheeling

1. What **benefits** of walking, cycling or wheeling do you see, compared to using a car?
    a. How about cycling? Check other form of active travel

2. What are the **disadvantages** of walking, cycling or wheeling, compared to using a car?

3. What would **enable** you to use these active ways to travel more?
    a. *Prompt:* I would use walking, cycling or wheeling more if…

4. Do you have enough information about opportunities for walking, cycling or wheeling in your area?
    a. For example, where cycle routes are located, or how to use the new public e-bike hire scheme. *Beryl bikes are available in Falmouth and Penryn. Newquay, Penzance, St Austell and Truro are coming in the spring.*
    b. *If no:* Do you have any suggestions for how the Council could support you to access this information?

5. Is active travel – e.g. walking, cycling or e-bikes – an option for getting to work, shops, etc? Why/Why not?

6. Are there any **barriers** that prevent or deter you from walking, cycling or wheeling?

    a. *If yes:* What are they and how do they affect you?

Structural barriers

a. Are there enough walking, cycling or wheeling routes for you to get around safely and easily?
b. Are these routes well-connected, so you can avoid using busy roads?
c. Is there enough space during busy times? Is the pavement or cycle path wide enough?
d. Are the routes well-lit, so you can use them when it is dark?
e. Are the routes kept in good condition so that people are unlikely to trip over, or lose balance on their bicycle, because of potholes/defects in the surface?
f. Are there sufficient secure places to lock up bikes in destinations?
g. Do the routes tend to become flooded after it rains?

Behavioural barriers

h. Does your motivation for walking, cycling or wheeling vary, depending on the weather or the season?
i. Do you feel confident in terms of your cycling ability or road awareness?

Behaviour of other users:

j. Do you feel safe when sharing roads, pavements or cycle paths with others?
    a. For example, do cars overtake you too close, or too fast?
k. Do cyclists cycle on the footpath/pavement?
l. Do motorcyclists use the cycle routes?

Congestion barriers

m. Do you often experience air pollution or vehicle fumes when walking, cycling or wheeling?
n. Do parked cars or loading vehicles often obstruct visibility or physical access?

7. Has anyone had the opportunity to try the new Beryl e-bikes?
    a. *Beryl bikes are available in Falmouth and Penryn. Newquay, Penzance, St Austell and Truro are coming in the spring.*
    b. *If yes:* How was your experience of using the bikes?
    c. Do you have any suggestions for improving the scheme?

4) Car sharing

Car clubs

1. Has anyone tried using a car club?
    a. *Car clubs are currently only available in Truro (2 cars), Bude (2 cars) and Falmouth (1 car). Maybe experience of using car clubs in other areas*

2. Even if you don't have direct experience of using a car club, what **advantages** do you see, compared to using your own car?

3. What are the **disadvantages** of using a car club, compared to using your own car??

4. What would **enable** you to use a car club?

5. Are there any **barriers** that prevent or deter you from using a car club?

Lift sharing

6. Does anyone regularly share lifts to get to work or on the school run?
    b. This could be an informal arrangement with friends or work colleagues
    c. Or using a lift-sharing platform, such as Carshare Cornwall or Liftshare
    d. Or a lift-share scheme organised by your employer

7. What **advantages** of using a liftshare scheme do you see, compared to using your own car?

8. What are the **disadvantages?**

9. What, if anything, would **enable** you to share lifts more?

10. Are there any **barriers** that prevent or deter you from sharing lifts with others?

5) Electric vehicles

1. Has anyone bought or considered buying an electric vehicle?
    a. *If yes:* why was this?
    b. *If no:* what has put you off?

2. What are the **advantages** of an electric car, compared to a petrol, diesel or hybrid car?

3. What are the **disadvantages** of an electric car, compared to a petrol, diesel or hybrid car?
    a. Are you worried about the range of electric vehicles?
    b. Do you think that it would be possible to charge an electric car easily when you need to? E.g. are there enough charge points, are they reliable?

4. What, if anything, would encourage or **enable** you to buy an electric vehicle?

5. Aside from the high cost of buying one, are there any **barriers** that prevent you buying an electric car?

6) Multi-modal travel

1. Does anyone change travel mode during a common journey, for example changing from a car to a bus, or from a train to a bicycle?
    a. *If yes:* Why do you change do a different mode of travel?

2. What are the **advantages** of multi-modal travel?

3. What are the **disadvantages** of multi-modal travel?

Group-specific questions

Young adults

1. Do you drive?
    a. *If yes:* do you have access to a car? This could be your own car, or a family member's car.
    b. *If no:* are you dependent on a family member or friend to give you lifts, or do you have other options such as public transport, walking or cycling?

2. Would you like to own a car in the future? Why; why not?

3. How important is owning or having access to a car for providing life opportunities?
    a. For example, do you need a car to get a job?
    b. Do you need a car to have a good social life, or to take part in sports clubs or hobbies?

4. For your situation, how feasible are the alternatives to owning a car - for example, public transport, or shared mobility such as car clubs or public bike hire schemes?
    a. *Go to Sections 2 (Public Transport) or 3 or 4 (Shared mobility)*

People with a health condition or disability

Some questions may already have been covered in previous discussion on Public Transport or Active Travel

Active Travel

1. Do you feel safe when using shared spaces such as cycle lanes or pavements?
    a. Is there enough space to share with other users? For example, are the pavements wide enough?
    b. Do other users give you enough room to get by?
    c. Do cyclists tend to slow down when overtaking you?
    d. Do you have any suggestions for what could improve this?

2. Have you experienced pavements, paths or cycle lanes being in a poor condition, or perhaps they are not well designed to enable you access to some places?
    a. How does this affect you?
    b. Does this happen often?
    c. Are there sufficient pedestrian crossings to allow you to cross busy roads?
    d. Is there usually step free access from the pavement when crossing roads?
    e. Do parked cars or loading vehicles sometimes obstruct visibility or physical access? For example, are you often obstructed by cars parked on pavements?
    f. Are there sufficient spaces/benches to stop and rest if you need to?
    g. Do the rest places provide shelter from the rain, or when it is very hot?
    h. Do you have any suggestions for what could improve this?

3. The Council often promotes walking, cycling or wheeling on their websites and activities, for example or the launch of Beryl e-bikes or the active travel challenge. Do you feel included in these efforts?
    a. If no: Do you have any suggestions for how the Council could improve things or make it easier for you to participate?

Public Transport

4. What is your experience of using public transport?
    a. Do buses and trains cater for your needs in terms of access? For example, low-floor entrances to get on and off the bus, or adequate space to position a wheelchair?
    b. Are there enough bus stops on the services you use to access shops, or to travel from the bus stop to your home?
    c. Is the information presented on the electronic displays at bus stops usually correct?

People on low incomes

1. Do you drive?
    a. *If yes:* do you have access to a car? This could be your own car, or a family member's car.
    b. *If no:* are you dependent on a family member or friend to give you lifts, or do you have other options such as public transport, walking or cycling?

2. How important is owning or having access to a car for providing life opportunities?
    a. For example, do you need a car to get a job?
    b. Do you need a car to have a good social life, or to take part in sports clubs or hobbies?

3. For your situation, how feasible are the alternatives to owning a car - for example, public transport, or shared mobility such as car clubs or public bike hire schemes?
    a. Go to Sections 2 (Public Transport) or 3 or 4 (Shared mobility)
    b. Do you find shared mobility schemes affordable?

4. What do you think of the cost of public transport fares?
    a. *If expensive:* Does this affect how often you choose to use the bus or train?

5. Do you depend on public transport for everyday activities, such as going to the shops or to visit the doctors?

Rural residents

Some questions may already have been covered in previous discussion on Public Transport

1. Do you regularly use public transport to get around?
    a. *If yes:* Which form of transport – bus, train, ferry?
    b. Are the ________ services in your area frequent enough for you to get to work, or to access local shops and services?
    c. Are the _______ services in your area reliable?
    d. *If no:* Does this affect your life opportunities? For example, what jobs you can apply for, whether you can go to the shops, or visit friends?

2. Are there any gaps in public transport services – do the bus routes go to the places you need to get to?

   a. Do you often have to walk a section of your journey to get to the bus stop, or ask someone for a lift?
   b. *If yes:* Does this situation affect which mode of travel you choose?

3. Do you usually have to change buses to complete your journey?
   a. Or switch between two different modes of travel, such as bike to bus, or bus to train?
   b. *If yes:* Does this situation affect which mode of travel you choose?

4. Is active travel – e.g. walking, cycling or e-bikes – an option for getting to work, shops, etc? (Why not?)

Urban residents

Some questions may already have been covered in previous discussion on Public Transport

1. Do you regularly use public transport to get around?
   a. *If yes:* Which form of transport – bus, train, ferry?
   b. Are the ________ services in your area frequent enough for you to get to work, or to access local shops and services?
   c. Are the _______ services in your area reliable?
   d. *If no:* Does this affect your life opportunities? For example, what jobs you can apply for, whether you can go to the shops, or visit friends?

2. Are the roads in your area often busy, so you end up stuck in traffic?
   a. *If yes:* Does this situation affect which mode of travel you choose?

3. Do you often experience air pollution or vehicle fumes in the area where you live?
   a. *If yes:* Does this situation affect which mode of travel you choose?

4. Do you usually have to change buses to complete your journey?
   a. Or switch between two different modes of travel, such as bike to bus, or bus to train?
   b. *If yes:* Does this situation affect which mode of travel you choose?

Home working

Check relevance depending on the respondent group.

We haven't discussed this yet, but avoiding travel altogether is one way of saving carbon emissions. The pandemic resulted in many people working from home, depending on the nature of their job.

1. Thinking about your situation now, would you say the amount of commuting you do, to and from work, has returned to pre-pandemic levels? Or is there more hybrid/home working now than before the pandemic?
    a. *If more hybrid/home working now:* Is it due to:
        i. your preference for how and where you work?
        ii. you now have a different job?
        iii. a change in how you work in your existing job / your employer requires it, or supports it?
        iv. new habits or life priorities which have developed over the past two years?

    b. *If returned to pre-pandemic work patterns:* Is this due to:
        i. your preference for how and where you work?
        ii. the nature or requirements of your job?
        iii. the expectations of your employer or manager?
        iv. something else?

2. Do you support these changes? Why/why not?

3. Do you think you will maintain them in the future? Why/why not?

4. Does your job role require you to travel – I'm not asking about commuting, but travelling for the job itself?
    a. *If yes:* Can you imagine changing how you work to reduce your need to travel? For example, scheduling all your in-person meetings on one day; or having more virtual meetings with customers.

Activity 2 - Visioning reduced car use (15 mins)

Agency

1. Do you feel you are able to reduce your car use if you wanted to?
    - Are alternatives modes of travel feasible for your situation? Why/why not?

2. Is there anything you think the Council should do which would make it easier for you to change your travel behaviour?

3. Do you have enough information about public transport services in your area?
    - *If no:* Do you have any suggestions for how the Council, or Public Transport Service Providers, could support you to access this information?

4. Do you have enough information about opportunities for walking, cycling or wheeling in your area?
    - For example, where cycle routes are located, or how to use the new public e-bike hire scheme. *Beryl bikes are available in Falmouth and Penryn. Newquay, Penzance, St Austell and Truro are coming in the spring.*
    - *If no:* Do you have any suggestions for how the Council could support you to access this information?

5. Has the cost of living crisis or rising fuel prices affected how often you drive, or how far?
    - *If yes:* In what situations have you reduced your car travel?
    - *Prompt:* For example, the reasons for travelling we discussed earlier: commuting, access to services, leisure or seeing friends, the school run – have you reduced car travel for any particular type of journey?

Lifestyles/habits

1. Does your daily or weekly routine determine how you choose to travel? For example, perhaps you do a regular activity every Tuesday evening, and therefore use a particular travel mode?
    - *If yes:* Have you ever considered alternative ways to get around, or do you tend to stick to what you know?

2. Did you notice if your travel behaviour changed after an important life event, for example moving house, changing your job, or having a child?
    - *If yes:* In what ways did it change?
    - Was this a conscious decision in response to your new circumstances, or was this change unintentional?

3. Can you imagine living without a car? Would this be possible?
    - Do you see any benefits to living without a car?
    - What would be the main drawbacks of living without a car?

4. Can you think of ways you would like to live differently, to change your lifestyle in some way, which would involve travelling less – but without giving up the car completely?

Social norms/neighbourhood effects/values

1. Do you notice people in your area regularly walking, cycling or wheeling to work, to the shops, or to school? So walking, cycling or wheeling as a mode of travel, not just for exercise or leisure?
    - *If yes:* Does this influence you in any way?

2. Do the expectations of family members, friends or work colleagues affect how you choose to travel?
    - *Rephrase:* Do you alter your travel behaviour depending on who you are travelling with, or what others might think of your travel choices?
    - *If yes:* In what ways did you change your travel behaviour, and why?
    - Have you experienced encouragement, or perhaps even pressure, to change or to not change your travel behaviour?

3. Have you ever deliberately changed how you travel to be more consistent with your values?
    - For example, have you avoided flying abroad for a holiday because of a concern about climate change?

Activity 3 - The role of policy (15 mins)

Ranking exercises as platform for discussion

3a) Pull mechanisms

Switch to Presenter Mode – Pull mechanisms. Check participants can see the question

The Council is considering a range of policy measures to reduce travel-related carbon emissions. On the screen you can see a list of some of these policies.

Selected 5 pull mechanisms

1. Investment in routes for pedestrians, cyclists, and mobility scooter/wheelchair users

2. Cheaper public transport
3. Shared mobility options, such as public bike hire (e.g. Beryl Bikes) or car clubs (e.g. Co Cars)
4. Investment in the public transport network (more frequent services/more bus routes)
5. Investment in electric vehicle infrastructure

Please could I ask you to rank these policies in order of preference. So, what would be your favourite policy, 2) what is your second most preferable, and so on.

Questions – pull mechanisms

1. Why have you put this policy at the top? Why should it be a priority for the Council?

2. What benefits do you see from policy_______?
    a. As well as benefits for individuals, can you think of any benefits for society or the environment?

3. What disadvantages do you see from policy ________?

4. Would any of these policies encourage you to use your car less? Why/why not?
    a. *If yes:* for what types of journeys?
    b. Which mode of travel would you switch to?

5. Has the reduced bus fares/integrated tickets in Cornwall encouraged anyone to use buses more frequently?
    c. Or encouraged anyone to start using buses for the first time?

6. Do any of these policies seem unrealistic to you? Why/why not?

7. Do any of them seem particularly fair or unfair? Why / why not?

3b) Push mechanisms

Here are some more policies which the Council may consider. So, the same exercise again, please rank these policies in order of preference: So, 1) would be your favourite policy, 2) your second favourite, and so on.

Selected 5 push mechanisms

1. Restrictions on car traffic – pedestrianised areas and low speed environments
2. Restrictions on car traffic in favour of public transport – bus lanes, bus gates
3. Low Emission Zones/Congestion charges

4. Increased parking charges in town centres and workplaces
5. Restricted parking in city centres and workplaces

Low emission zones would be focused on the busier urban areas where P&R facilities/ public transport alternatives are available (e.g. Truro, St Ives) rather than zones more generally applied.

Questions – push mechanisms

1. Why have you put this policy at the top? Why should it be a priority for the Council?

2. This group of policies function in one of two ways:
   a. by restricting where people can drive or park
   b. or by making it more expensive to drive a car in some areas

4. Do you think the Council should be imposing restrictions on car use in these ways? Why/why not?

3. What benefits do you see from policy_______?
   a. As well as benefits for individuals, can you think of any benefits for society or the environment?

4. What disadvantages do you see from policy ________?

5. Would any of these policies encourage you to use your car less? Why/why not?
   c. *If yes:* for what types of journeys?
   d. Which mode of travel would you switch to?

6. Do you think some individuals should be exempt from any of these policies? Why/why not?
   e. *Rephrase:* Do any of them seem particularly fair or unfair? Why/why not?

7. Do you think people in Cornwall might be opposed to policy _______?
   f. *If yes:* do you have any suggestions for how the Council should engage with people to change their travel behaviour?

8. Do any of these policies seem unrealistic to you? Why/why not?

Wrap up (5 mins)

1. Thinking about the topics we've covered, is there anything else you would like to discuss?

2. Are there any questions that I've missed? To put it another way: What is the question that I should be asking you about your experience of travel in Cornwall?
    a. The unknown unknowns

Debrief

I would like to thank you all very much for taking the time to participate in this study. The information you have provided will be used to inform Cornwall Council policies or interventions to reduce travel-related carbon emissions. All your comments will remain anonymous.

After this meeting has finished, if you have any further suggestions or comments you would like to make about travel options in Cornwall, or if you have any questions about the study, please feel free to contact me.

Later on today, you will receive two emails – the first will be your 'Love to shop' voucher code, the second will be from me. Please could I ask you to reply to my email, confirming receipt of the voucher – so I can show my accounts department that I haven't kept all the vouchers for myself.

Thanks again for taking part, enjoy the rest of your day.

Focus group exit survey

Q1. Do you own or have regular access to a car?

- Yes - an electric or hybrid car
- Yes - a petrol or diesel car
- No

Q2. What is the first half of your postcode (e.g. TR1, PL14)? _____________

Q3. Approximately how far (in miles) is your home to your place of work / study? ________

- N/A – I don't work, or I work from home

Q3. What is your age (in years)? __________

Q4. How do you self-identify?

- Female

- Male
- Non-binary
- None of the above (if you wish, please specify) ______________
- Prefer not to say

Q5. Do you have a long-standing illness, injury or disability that limits your normal day-to-day activities?

By 'long-standing' we mean anything that has troubled you over a period of time. 'Normal day-to-day activities' includes things like eating, washing, walking and going shopping.

- Yes
- No
- Prefer not to say

Q6. What is the highest level of education you have achieved so far?
- No formal qualifications
- GCSE or O-level
- A-level
- Undergraduate degree (e.g. Bachelor)
- Postgraduate degree (e.g. Master, PhD)
- Vocational qualification
- Other
- Prefer not to say

Q7. Which option best describes your employment status?
- Employed full time (30+ hrs/wk)
- Employed part time (less than 30 hrs/wk)
- Self-employed
- Unemployed
- Looking after home / family
- Studying
- Retired
- Other
- Prefer not to say

Q8. Please indicate the approximate combined income of your household (per year, before tax deductions):
- Less than £6,000

- £6,000 - £12,999
- £13,000 - £18,999
- £19,000 - £25,999
- £26,000 - £31,999
- £32,000 - £47,999
- £48,000 - £63,999
- £64,000 - £95,999
- More than £96,000
- Prefer not to say
